# Chiral Brønsted Acid‐Catalyzed Asymmetric Synthesis of *N*‐Aryl‐*cis*‐aziridine Carboxylate Esters

**DOI:** 10.1002/anie.201611990

**Published:** 2017-04-05

**Authors:** Sean P. Bew, John Liddle, David L. Hughes, Paolo Pesce, Sean M. Thurston

**Affiliations:** ^1^ School of Chemistry Norwich Research Park University of East Anglia Norwich NR4 7TJ UK; ^2^ Department of Medicinal Chemistry GlaxoSmithKline Gunnels Wood Road Stevenage Hertfordshire UK

**Keywords:** asymmetric catalysis, aziridine, Brønsted acids, multicomponent reactions

## Abstract

We report a multi‐component asymmetric Brønsted acid‐catalyzed aza‐Darzens reaction which is not limited to specific aromatic or heterocyclic aldehydes. Incorporating alkyl diazoacetates and, important for high ee's, ortho‐tert‐butoxyaniline our optimized reaction (i.e. solvent, temperature and catalyst study) affords excellent yields (61–98 %) and mostly >90 % optically active cis‐aziridines. (+)‐Chloramphenicol was generated in 4 steps from commercial starting materials. A tentative mechanism is outlined.

Such is the versatility of organocatalysis and its ability to mediate a plethora of diverse reaction types^1^ it is, now, an indispensable “tool” in the synthetic chemists “toolbox”.^2^ Indeed, improving atom‐ and reaction‐efficiency is a key driver to developing new reactions and protocols; in this context organocatalysis has demonstrated its importance by efficiently mediating many different convergent reactions or multi‐component syntheses. The work here supports these aspects by generating structure and function‐diverse motifs via fewer synthetic, isolation and purification steps.

Optically active aziridines have many diverse uses, especially as key intermediates^3^ “on route” to important “secondary” products for example, α‐/β‐amino acids, polymers, azasugars, auxilaries, oxazolidinones, imidazolidines, β‐lactams and pyrrolidines. Further applications include synthesis of non‐aziridine containing bioactive compounds for example, kainoids, (−)‐mesembrine, (−)‐platynesine, actinomycin and feldamycin, in addition to synthetic bioactive aziridines for example, NSC676892 as well as natural products for example, azinomycin and maduropeptide.^4^

Using a BINOL *N*‐triflylphosphoramide Brønsted acid a 61–98 % yielding asymmetric aza‐Darzens reaction affords *N*‐aryl‐*cis*‐aziridines in, mostly, 90–99 % *ee*. The reaction is straightforward to set up and has minimal requirements for strictly anhydrous or anaerobic conditions, furthermore it does not require organocatalyst pre‐generation or activation, or an “activated” arylglyoxal starting material. Exploiting the protocol synthesis of aziridines based on **4** uses readily generated or commercially available aldehydes (**1**), amines (**2**) and alkyl diazoacetates (**3**, Scheme 1).

**Scheme 1:**
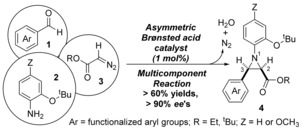
Multicomponent asymmetric synthesis of *N*‐aryl‐*cis*‐aziridines **4**.

Activating the C=N bond of an imine with a BINOL phosphoric acid^5^ lowers its LUMO energy and generates an imminium ion pair that can, but not always will, react with a nucleophile. Seminal work by Akiyama et al. established chiral BINOL phosphoric acids [p*K*_a_≈13 (CH_3_CN)^6^] activate aldimines (derived from, specifically, arylglyoxals and *p*‐anisidine) and react with ethyl diazoacetate (EDA) affording *cis*‐aziridines in 92–97 % *ee*.^7^ Similarly, other Brønsted acids^8^ and pyridinium triflate activate a diverse array of imines, including for example, 2‐pyridyl derived **5**, enabling the presumed imminium ion‐pair (not shown) to react with EDA and afford *cis*‐*rac*‐aziridine (**8**, 83 % yield) (Scheme 2).^9^ With these racemic studies complete our focus shifted to developing a substrate enhanced and diverse, multi‐component asymmetric aza‐Darzens reaction. Inspired by the work of Akiyama et al.^7^ and the Mannich reaction reported by Yamanaka et al. we considered the inclusion of **5** may generate a constrained hydrogen‐bonded and activated complex similar to **11**; we were drawn to the use of **5** to generate **11** due to similarities in the chiral non‐racemic rigid environment proposed by Yamanaka (using a *N*‐(2‐hydroxyphenyl)imine starting material).^10^ Screening chiral non‐racemic BINOL and VANOL phosphoric acids, as well as a H‐QUIN‐BAM triflate salt^11^ we were disappointed no reactions were observed. We attribute the failure using **5**, as well as other alternative imines, to the low p*K*_a_’s of the Brønsted acids and their inability to generate a sufficiently “activated” form of **10** or **11**.

**Scheme 2:**
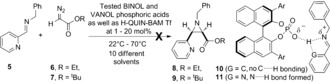
Failed attempts at synthesising *cis*‐**8** and *cis*‐**9**.

Switching to the more acidic BINOL *N*‐triflylphosphoramides for example, p*K*_a_ **14** ≈6 (CH_3_CN)^6^ (Figure 1) the synthesis of (*S*)‐3,3′‐*bis*(phenyl)‐**14**, (*S*)‐3,3′‐*bis*(4‐methylphenyl)‐**15** and sterically encumbered (*S*)‐3,3′‐*bis*(4*‐tert*‐butylphenyl)‐**16** was straightforward.^12^ By using 10 mol % all three catalysts, independently, at room temperature mediated the synthesis of *cis*‐aziridine **8** in 73 %, 85 % and 87 % yields, respectively. ^1^H‐NMR of the unpurified reactions confirmed no enamide^5^ i.e. *Z*‐**12** or Z‐**13** (Figure 1) or *trans*‐**8** (*J*_2,3_≈2 Hz, not shown) had formed. Disappointingly, chiral column HPLC analysis established *cis*‐**8** was racemic when generated using **14** or **15**; in contrast, **16** afforded non‐racemic *cis*‐**8** but in a poor 16 % *ee* (Table 1, Entries 1–3 respectively). Clearly, the bulky 4*‐tert*‐butyl group had a positive stereochemical advantage over **14** and **15**. Increasing 3,3′‐steric congestion at the 2‐ and 6‐ positions using (*S*)‐3,3′‐*bis*(2,4,6‐triisopropyl)phenyl‐**17** returned *cis*‐**8** in excellent yield and increased 23 % *ee* (entry 4).

**Figure 1:**
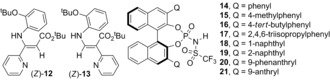
Enamides and 3,3′‐*bis*(aryl) (*S*)‐BINOL *N*‐triflylphosphoramides.

**Table 1:** Probing the asymmetric synthesis of *cis*‐**8** and *cis*‐**9** using **14**–**21**.

Entry	Catalyst	**8** (R=Et, *ee*)		Entry	Catalyst	**9** (R=^t^Bu, *ee*)
1	**14**	racemic		9	**14**	racemic
2	**15**	racemic		10	**15**	18 %
3	**16**	16 %		11	**16**	13 %
4	**17**	23 %		12	**17**	racemic
5	**18**	28 %		13	**18**	20 %
6	**19**	26 %		14	**19**	20 %
7	**20**	35 %		15	**20**	22 %
8	**21**	47 %		16	**21**	31 %

The 69 % increase in *ee* when 2‐ and 6‐isopropyl groups were incorporated (**17**) suggests these “lateral” positions have key roles in reaction stereoselectivity. Probing this, multicyclic 1‐naphthyl (**18**), 2‐naphthyl (**19**), 9‐phenanthryl (**20**) and 9‐anthryl (**21**) were incorporated (10 mol %) into our “test” reaction (Scheme 2). All afforded excellent yields of *cis*‐**8**. A gradual *increase* in *ee* was observed as the “lateral” groups were added. Thus catalyst **14** afforded *rac*‐**8**, whereas 1‐naphthyl‐**18** offered *cis*‐**8** with a 28 % *ee*. An almost identical 26 % *ee* was provided by 2‐naphthyl‐**19** and 9‐phenanthryl‐**20** gave an improved 35 % *ee*, finally, 9‐anthryl‐**21** generated *cis*‐**8** in a respectable 47 % *ee*.

Encouraged by the results with **6**, sterically encumbered *tert*‐butyl ester **7** was investigated. A gradual increase in *ee* was observed but, overall, the levels of stereoinduction were, generally, inferior. So, *N*‐benzyl **5** was substituted for a rotationally *less* flexible *N*‐4‐(methoxyphenyl) or *N*‐PMP group. Reacting the corresponding imine (not shown) with **7** mediated by **21** afforded the *cis*‐aziridine in an 81 % yield (*J*_2,3_ 6.8 Hz). Further verifying the importance of including the, presumed, rotationally less flexible *N*‐PMP the product was afforded with a significantly improved 67 % *ee*. Exchanging the *N*‐PMP for the regioisomeric *N*‐2‐methoxyphenyl imine **22** (Scheme 3) its activation (**21**) and reaction with *tert*‐butyl ester‐**7** afforded *cis*‐**25** with a 72 % *ee*. Evidently, the 2‐methoxyphenyl had a positive influence on the stereochemical outcome of the aza‐Darzens reaction. The steric effect was probed using 2‐isopropoxyphenyl‐**23**, 2‐n‐butoxyphenyl (not shown) and 2‐*tert*‐butoxyphenyl‐**24** (Scheme 3) each reacted, independently, with **7** and **21**. In this series and at ambient temperature the *tert*‐butoxy group on **24** afforded *cis*‐**27** with a 74 % *ee*.

**Scheme 3:**
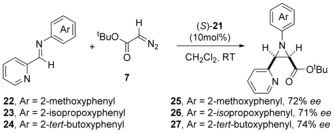
Asymmetric synthesis of *N*‐(alkoxyphenyl)‐*cis*‐aziridines **25**–**27**.

A solvent and temperature study using 1 mol % of **21** established chloroform at −60 °C was the optimum combination for transforming 2‐(*tert*‐butoxyphenyl)‐**24** into *cis*‐**27** with an excellent 98 % *ee* and 95 % yield. Probing the catalytic activity of **21** at 0.5 and 0.25 mol % loadings the reaction times increased to 48 and 62 hours. In both examples *cis*‐**27** was afforded in very similar 87 %/86 % *ee* and 98 %/95 % yield, respectively.

The synthesis of **38**–**47** (Scheme 4) was examined using **21** (1 mol %) in CHCl_3_ at −60 °C. Incorporating (*E*)‐2‐(*tert*‐butoxyphenyl)‐**28** *cis*‐**38** was afforded in an excellent 91 % *ee* and 90 % yield. Confirming reaction versatility electron‐withdrawing 4‐cyano imine‐**30** and 4‐nitrophenyl imine‐**31** were transformed into *cis*‐**40** and *cis*‐**41** with excellent optical purities both 98 % and yields that is, 98 % and 97 % respectively (Scheme 4). Similarly, electron‐rich 4‐hydroxybenzaldehyde (*O*‐Fmoc protected) afforded *cis*‐**42** in a 90 % *ee* and 91 % yield. *Cis*‐**43** to *cis*‐**47** were synthesized in excellent yields and *ee*’s; 4‐bromophenyl‐*cis*‐**45** (93 % *ee*) and 4‐iodophenyl‐*cis*‐**46** (92 % *ee*) appear readily amenable to further elaboration via transition‐metal mediated transformations.

**Scheme 4:**
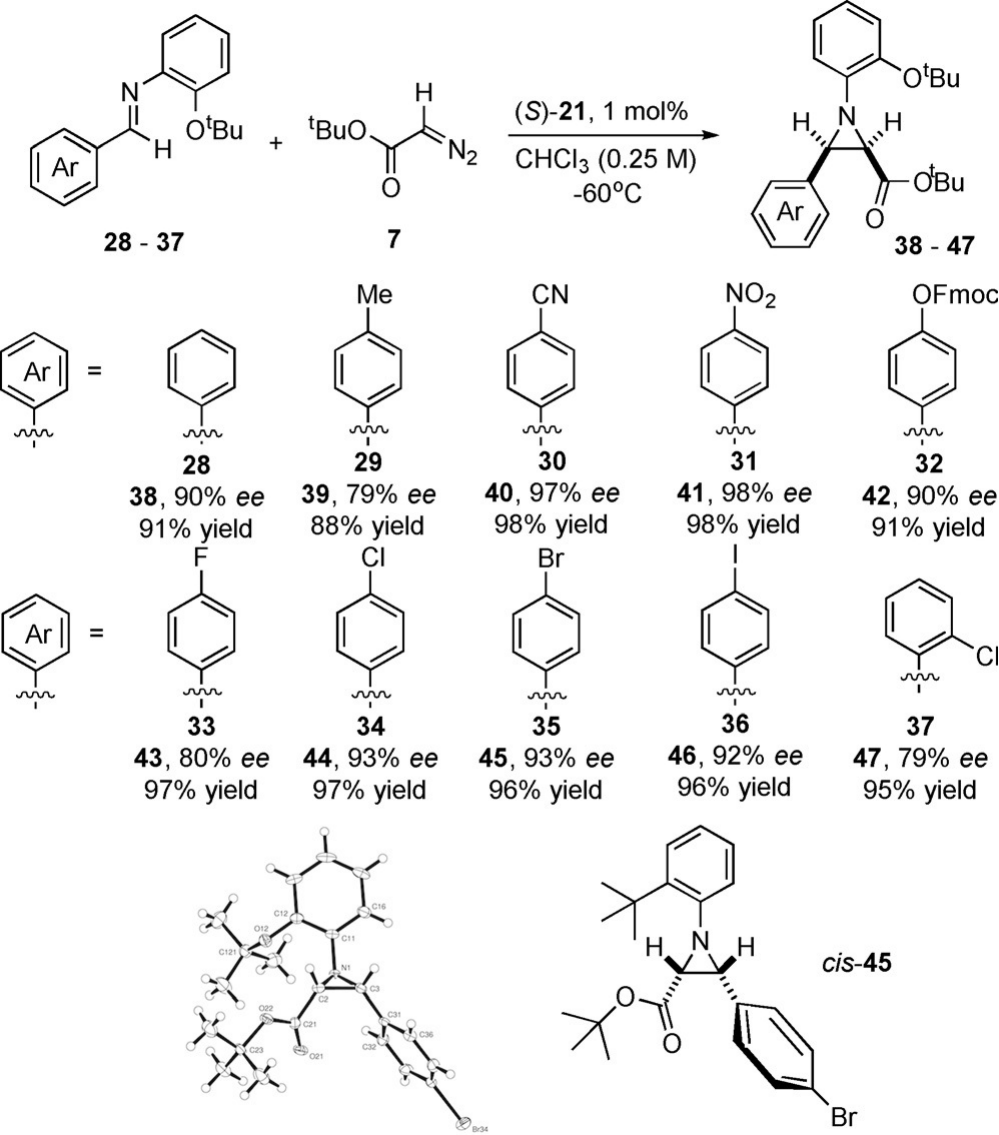
Asymmetric synthesis of structure and function diverse *N*‐(2‐*tert*‐butoxyphenyl)‐*cis*‐aziridines **38**–**47** and the X‐ray structure of *cis*‐**45**.

The magnitude of an aziridine coupling constant (*J*_2,3_) indicates the relative stereochemical assignment of the C_2,3_‐substituents that is, *J*_2,3_ 5–9 Hz=*cis* and 2–6 Hz=*trans*. For **38**–**47** we tentatively assigned a *cis*‐stereochemical relationship; confirming this was essential. Recrystallising **45** [*J*_2,3_ 6.7(4) Hz] afforded colorless orthorhombic plates. X‐ray diffraction established the *cis*‐stereochemical relationship between the 4‐bromophenyl and the *tert*‐butyl carboxylate ester (Scheme 4).^13^

Generating aziridines via multicomponent asymmetric syntheses is advantageous, they are however, still, rare.^14^ It was crucial to verify **21** mediated the multicomponent synthesis of *cis*‐aziridines. A three‐component, two‐step, one‐pot protocol generated 2‐pyridyl‐**27**, 4‐cyanophenyl‐**40** and 4‐nitrophenyl‐**41** in excellent yields that is, 74–96 % and *ee*’s that is, **27** (96 %) **40**, (99 %) and **41** (96 %, Scheme 5). These *ee*’*s* are, within experimental error, identical to those generated via the *pre‐synthesis* imine route (Scheme 4). Incorporating 4‐thiomethyl‐, 4‐trifluoromethyl‐ and pentafluorobenzaldehyde afforded *cis*‐**49** to *cis*‐**51**. The efficient synthesis of thioether *cis*‐**49** is worthy of note; Davis et al. exploited similar (*S*)‐*N*‐(4‐toluenesulfinyl)‐derived aziridines transforming them into thiamphenicol and florfenicol.^15^

**Scheme 5:**
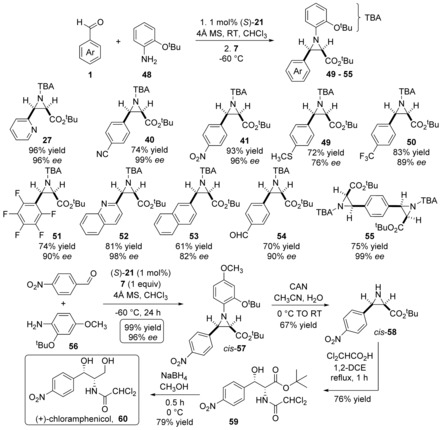
Asymmetric synthesis of *cis*‐aziridines and (+)‐chloramphenicol.

4‐Trifluoromethylbenzaldehyde and pentafluorobenzaldehyde afforded *cis*‐**50** and *cis*‐**51** in excellent 89 % and 90 % *ee*’s, respectively. Integrating bicyclic quinoline‐2‐carboxyaldehyde was also straightforward; optically active *cis*‐**52** was afforded in a 98 % *ee*. Interestingly, the formation of *cis*‐**27** and *cis*‐**52** was faster than for example **40**, **41**, **49**–**50**; the rapid evolution of, presumably, N_2_ was attributed to formation of a more reactive *intramolecular* chelated hydrogen bond (cf. **11**). Combining benzene‐1,4‐dicarboxyaldehyde and **48** (1 equiv) a one‐pot, single asymmetric aziridination afforded mono‐aziridine *cis*‐**54**. Alternatively, 2 equiv of **48** generated bis‐aziridine *cis*‐**55**. Both reactions worked very well, *cis*‐**54** was afforded in a 70 % yield and 90 % *ee* and bis‐aziridine *cis*‐**55** with a 99 % *ee*. Seemingly, the installation of the second aziridine on optically active *cis*‐**54** to generate *cis*‐**55** was not negatively influenced by the first optically active *cis*‐aziridine. (−)‐Chloramphenicol is an important natural product with antibiotic properties. A one‐pot multicomponent aziridination using 4‐nitrobenzaldehyde, amine **56** and *tert*‐butyl diazoester **7** afforded *cis*‐**57** in near quantitative yield and an excellent 96 % *ee*. By using cerium(IV) ammonium nitrate in aqueous acetonitrile an important objective was to establish the cleavage “potential” of the 2‐*tert*‐butoxy‐4‐methoxyphenyl on *cis*‐**57**; NH‐*cis*‐**58** was afforded in an unoptimized 67 % yield, this was ring‐opened to amide **59** with dichloroacetic acid, finally reducing the *tert*‐butyl ester generated primary alcohol **60** (79 % yield). Physicochemical analysis and comparison with the literature confirmed (+)‐chloramphenicol **60** had been synthesized using (*S*)‐**21** in 4 steps and an overall 40 % yield.^16^

Scheme 6 outlines a tentative mechanism for *cis*‐aziridine diastereoselectivity. Initial *N*‐protonation of **31** via Brønsted acid (*S*)‐**21** [p*K*_a_≈6 (CH_3_CN)],^6^ affords imminium‐phosphoramide anion **60** (Path A, Scheme 6) whilst the weaker triflate salts and phosphoric acids (see Supporting Information, page 3) do not form sufficiently reactive imminium‐triflate/phosphate anions.

**Scheme 6:**
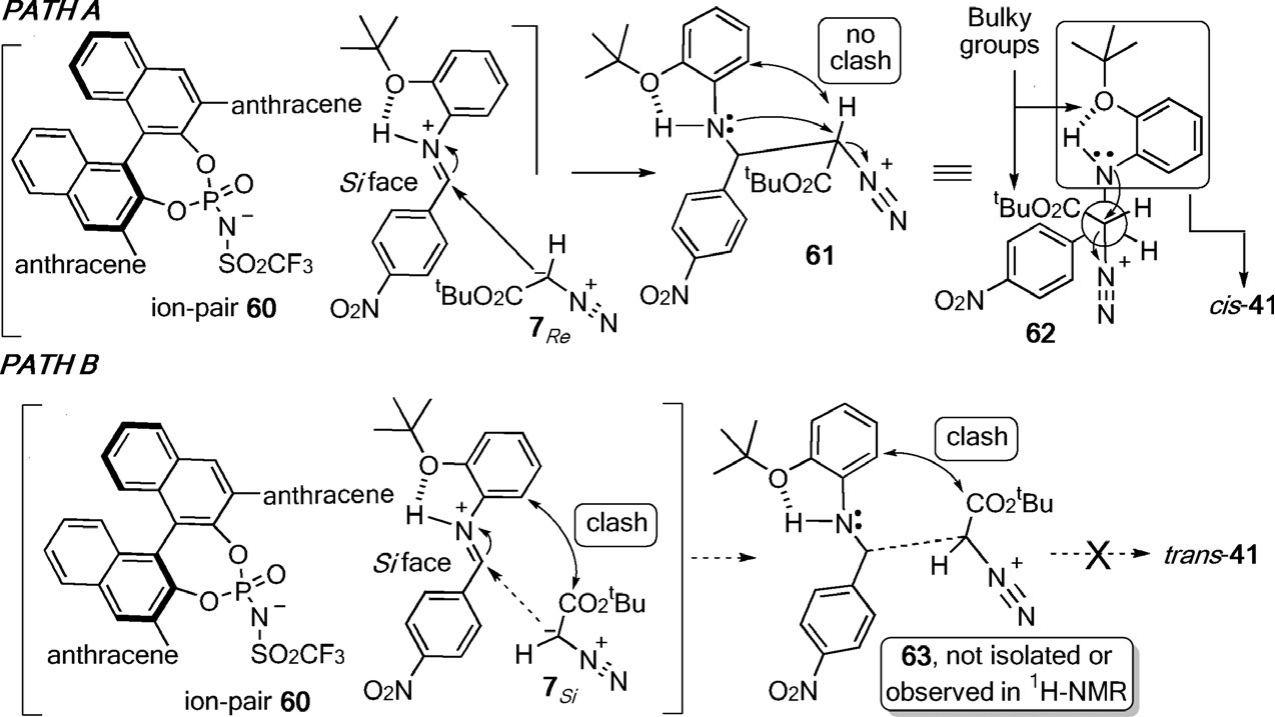
Mechanistic rational for the synthesis of *cis*‐**41** and not *trans*‐**41**.

Supporting protonation, not hydrogen‐bond activation,^17^ Houk et al. described a mechanism and origins of catalysis DFT and experimental study in which a similarly *N*‐protonated, to **60**, reactive hydrazonium‐phosphoramide^18^ anion (not shown) was formed from a BINOL *N*‐triflylphosphoramide and a hydrazone. Activation of **31** is crucial; the widely accepted aza‐Darzens mechanism^19^ invokes attack of a diazo nucleophile (i.e. **7**) on an imminium cation (i.e. **60**) generating an α‐diazonium β‐amino ester (i.e. **61**, see Path A). The importance of the latter, from a reaction kinetics and enantioselectivity point of view has been established by the reluctance of these intermediates to undergo a retro‐Mannich reaction.^20^ Generating, presumed, kinetic product **61** with excellent enantioselectivity is possible only if **7**, with its heterotopic faces that is, **7**_*Re*_ and **7**_*Si*_, efficiently discriminates between the *Si* and *Re* faces of optically active **60**. Path A outlines how *anti*‐diazonium intermediate **62** (Scheme 6) forms when the sterically encumbered heterotopic **7**_*Re*_ face approaches the *Si* face of imine **60** minimising the steric interactions between the intramolecularly hydrogen bonded bulky *ortho*‐(*tert*‐butoxy)phenyl imminium and the *tert*‐butyl ester on **7**_*Re*_. Although we have no direct evidence (^1^H‐NMR) for the backbone rigidifying hydrogen bond in **60** similar intramolecular hydrogen bonds in *ortho*‐substituted Schiff base's are known.^21^ Newman projection **62** affords a detailed depiction of the minimized steric interactions between the *tert*‐butyl ester and *ortho*‐*tert*‐butylphenyl ether. An intramolecular S_N_2 cyclization (release of N_2_) between the *anti*‐periplanar amine and diazonium groups affords *cis*‐**41**. Path B proceeds via ion‐pair **60**, however approach of **7**_*Si*_ onto the imine *Si* face is, now, inhibited by the two sterically bulky groups. Thus, formation of α‐diazonium β‐amino ester **63** and *trans*‐**41** is disfavoured. The crude ^1^H‐NMRs of our reactions afforded no evidence of *trans*‐**41** or α‐diazonium β‐amino ester **63**.

## Experimental Section

A flame dried Radleys tube and stirrer bar was charged with 4‐cyanobenzaldehyde (34 mg, 0.26 mmol) and 2‐*tert*‐butoxy‐phenylamine (43 mg, 0.26 mmol). Anhydrous chloroform (1 mL) and (*S*)‐**21** (2 mg, 0.0025 mmol, 1 mol %) were added followed by 40 mg of freshly powdered 4 Å molecular sieves. The reaction was stirred for 6 hours. Cooling the tube to −60 °C, **7** (40 μL, 0.29 mmol) was added via syringe. The reaction was stirred at −60 °C and monitored via TLC (hexane/ether:80/20) until the starting materials had been consumed. In vacuo removal of solvent allowed flash purification on silica gel (hexane/ether:80/20). Physicochemical analysis confirmed the identity of the solid as *cis*‐**40**. Chiral column analytical HPLC established *cis*‐**40** had an *ee* of 99 %.

## Conflict of interest

The authors declare no conflict of interest.

## Supporting information

As a service to our authors and readers, this journal provides supporting information supplied by the authors. Such materials are peer reviewed and may be re‐organized for online delivery, but are not copy‐edited or typeset. Technical support issues arising from supporting information (other than missing files) should be addressed to the authors.

Supplementary
