# Supplementary material for: Chiral Brønsted Acid‐Catalyzed Asymmetric Synthesis of N‐Aryl‐cis‐aziridine Carboxylate Esters
Source: Angew Chem Int Ed Engl. 2017 Apr 5;56(19):5322–6. doi: 10.1002/anie.201611990 (PMC5502890; doi:10.1002/anie.201611990)
Supplement: Supplementary file 1 — Supplementary [file ANIE-56-5322-s001.pdf]

Angewandte Chemie
Eine Zeitschrift der Gesellschaft Deutscher Chemiker

Supporting Information

Chiral Brønsted Acid-Catalyzed Asymmetric Synthesis of *N*-Aryl-*cis*-aziridine Carboxylate Esters

Sean P. Bew, John Liddle, David L. Hughes, Paolo Pesce, and Sean M. Thurston*

anie_201611990_sm_miscellaneous_information.pdf

Contents:

S1 – Index of contents.
S2 - General information
S3 - Structures of optically active BINOL, VANOL phosphoric acids & H-QUIN-BAM triflate and extra examples for Scheme 3
S4 - Synthesis of **27**
S5 - Synthesis of **38**
S6 - Synthesis of **39**
S7 - Synthesis of **40**
S8 - Synthesis of **41**
S9 - Synthesis of **42**
S10 - Synthesis of **43**
S11 - Synthesis of **44**
S12 - Synthesis of **45**
S13 - Synthesis of **46**
S14 - Synthesis of **47**
S15 - Synthesis of **40**
S16 - Synthesis of **38**
S17 - Synthesis of **49**
S18 - Synthesis of **50**
S19 - Synthesis of **51**
S20 - Synthesis of **51**
S21 - Synthesis of **52**
S22 - Synthesis of **53**
S23 - Synthesis of **54**
S24 - Synthesis of **55**
S25 - Synthesis of **57**
S26 - Synthesis of **58**
S27 - Synthesis of **59**
S28 - Synthesis of **60**
S29 - S74 ^{1}H-NMR & ^{13}C-NMR of optically active aziridines

General Information

All solvents and reagents were purchased from Aldrich or Alfa Aesar and used as received. Analytical thin-layer chromatography was performed on Merck Silica gel 60 F_{254} plates, with visualization by UV light and/or potassium permanganate stain followed by heating. Flash column chromatography was performed using Davisil 40-63 µm silica gel. ^{1}H- & ^{13}C-NMR spectra were recorded on a Bruker Avance 500 (500 MHz) spectrometer. Chemical shifts are reported in δ (ppm) and referenced to residual solvent signals ^{1}H-NMR: $CDCl_3$ at 7.26 ppm; ^{13}C-NMR: $CDCl_3$ at 77.16 ppm. Signal multiplicities are described as follows: s = singlet, d = doublet, dd = doublet of doublets, t = triplet, q = quartet, m = multiplet, br = broad. HPLCs were run on a Hewlett Packard 1100 with a DAD. The reactions were cooled using a Julabo chiller (FT900) with an external temperature probe using acetone in the cooling dewar. FTIR spectra were recorded on a Perkin Elmer Spectrum 100 spectrometer. MALDI-TOF mass spectra were recorded on a Shimadzu Axima-CFR spectrometer. HRMS were carried out by the EPSRC National Mass Spectrometry Facility, Swansea, UK. Melting points were recorded using Stuart Scientific SMP1 apparatus and are uncorrected.

Structures of the BINOL and VANOL phosphoric acids,

*and H-QUIN BAM triflate used to screen imine **5** for the synthesis of **8/9***

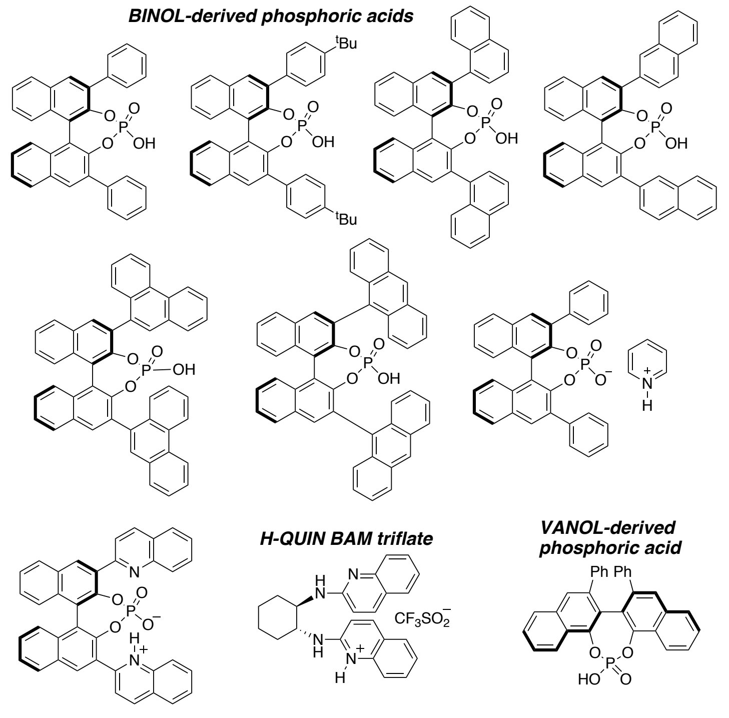

Further examples of N-Ar groups incorporated into the aza-Darzens reactions using presynthesised imines (view in conjunction with Scheme 3)

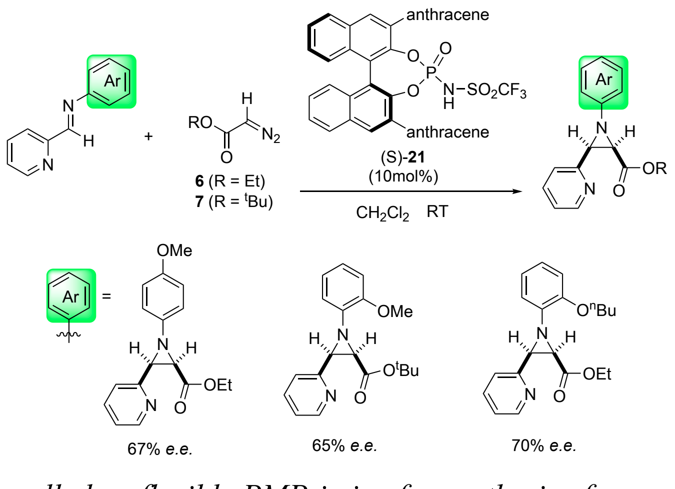

Use of rotationally less flexible PMP-imine for synthesis of corresponding cis-aziridine (to be read in conjunction with text concerning Scheme 3)

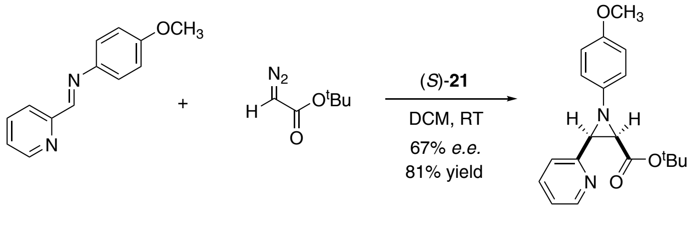

Synthesis of 27

A Radley carousel tube was charged with a magnetic stirrer bar. The tube was flame dried and then cooled to room temperature under an inert gas using a Schlenk line. (*E*)-2-*tert*-Butoxy-*N*-(pyridin-2-ylmethylene)phenylamine (66 mg, 0.26 mmol) and (S)-**21** (2.1 mg, 0.0026 mmol, 1 mol%) were loaded and three vacuum / nitrogen cycles were applied using a Schlenk line. Anhydrous chloroform (1 mL) was injected and the reaction tube placed in a cooling bath at −60 °C. A flux of an inert gas was allowed from the Schlenk line into the reaction tube until the cooling had taken place. The solution was stirred for one minute before *tert*-butyl diazoacetate (0.038 mL, 0.28 mmol, 1.1 eq^n) was added *via* syringe. The solution was stirred at −60 °C for a further 12 hours. Subsequent LC-MS analysis indicated the starting material (imine) had been completely consumed. Chloroform was removed *in vacuo* and the residue purified *via* flash chromatography on silica gel (elution - hexane / ether : 80 / 20). A sample of **27** was submitted to chiral column analytical HPLC analysis [Chiralpak AD, heptane / ethanol : 75 / 25, 1 mL / min, 4.0 min (1st peak), 7.5 min (2nd peak)]. The reaction afforded **27** as a colourless oil in a 96% yield and 96% *e.e.*

Characterization of chiral non-racemic **27**.

^{1}H NMR ($CDCl_3$, 400 MHz) 8.47 (d, J = 4.06 Hz, 1H), 7.68-7.56 (m, 2H), 7.13 (t, J = 5.84 Hz, 1H), 6.99-6.92 (m, 1H), 6.91-6.83 (m, 3H), 3.59 (d, J = 6.81 Hz, 1H, C_2-*H*), 3.07 (d, J = 6.81 Hz, 1H, C_3-*H*), 1.31 (s, 9H, $OC(CH_3)_3$), 1.17 (s, 9H, $OC(CH_3)_3$); ^{13}C NMR ($CDCl_3$, 75 MHz) 166.7, 155.6, 148.7, 148.1, 145.7, 135.9, 132.2, 123.1, 122.9, 122.7, 122.5, 120.8, 81.2, 80.1, 48.4, 46.8, 28.5, 27.6; $[\alpha]_D^{26} = -24.0$ (c = 1.0, $CHCl_3$); IR (thin film, cm^{-1}): 3063, 2977, 2933, 2246, 1923, 1742, 1591, 1569, 1489, 1451, 1436, 1393, 1368, 1262, 1224, 1152, 1112, 1093, 1048, 995, 975, 913, 887, 851, 819, 801, 749, 646; MS (EI)$^+$: *m/z* 369.2 (100%) $[M+H]^+$; HRMS (EI)$^+$: exact mass calculated for $[C_{22}H_{28}N_2O_3H]^+$ requires *m/z* 369.2173, found *m/z* 369.2174.

Synthesis of 38

A Radley carousel tube was charged with a magnetic stirrer bar. The tube was flame dried allowed to cool to room temperature under an inert gas using a Schlenk line. (*E*)-2-*tert*-Butoxy-*N*-(benzylidene)phenylamine **28** (66 mg, 0.26 mmol) and (S)-**21** (2.1 mg, 0.0026 mmol, 1 mol%) were loaded into the tube and three vacuum / nitrogen cycles were applied using the Schlenk line. Anhydrous chloroform (1 mL) was injected into the carousel reaction tube which was then placed in the cooling bath at −60 °C. A flux of an inert gas was allowed from the Schlenk line into the reaction tube until the cooling had taken place. The solution was stirred for one minute before *tert*-butyl diazoacetate (0.038 mL, 0.28 mmol, 1.1 eq) was added *via* syringe. The solution was stirred at −60 °C for a further 12 hours. Subsequent LC-MS analysis indicated the starting (imine) was completely consumed. Chloroform was removed *in vacuo* and the resulting residue purified *via* flash chromatography on silica gel (elution with hexane / ether : 80 / 20). A sample of **38** was submitted to chiral column analytical HPLC analysis [Chiralpak AD, *iso*-hexane / *iso*-propanol : 95 / 5, 1 mL / min, 3.9 min (1st peak), 7.8 min (2nd peak)]. The reaction afforded **38** in a 91% yield and 90% *e.e.*

Characterization of chiral non-racemic **38**.

^{1}H NMR ($CDCl_3$, 400 MHz) 7.45 (d, *J* = 6.84 Hz, 2H), 7.25 (t, *J* = 7.04 Hz, 2H), 7.21-7.16 (m, 1H), 6.95-6.92 (m, 1H), 6.90-6.85 (m, 3H), 3.41 (d, *J* = 6.78 Hz, 1H, C_2-*H*), 2.97 (d, *J* = 6.79 Hz, 1H, C_3-*H*), 1.31 (s, 9H, OC(CH_3)$_3$), 1.12 (s, 9H, OC(CH_3)$_3$); ^{13}C NMR ($CDCl_3$, 75 MHz) 167.3, 148.1, 146.8, 135.4, 128.2, 127.9, 127.6, 123.4, 123.3, 123.1, 121.1, 81.4, 80.5, 47.6, 47.5, 28.8, 27.9; $[\alpha]_D^{21}$ = -36.4 (c = 2.1, $CHCl_3$), IR (thin film, cm^{-1}): 2977, 2931, 1745, 1716, 1592, 1490, 1450, 1391, 1367, 1262, 1161, 1111, 1047, 886, 752; MS (EI)$^+$: *m/z* 368.1 $[M+H]^+$, 390.2 $[M+Na]^+$, 757.3 $[2M+Na]^+$; HRMS (EI)$^+$: exact mass calculated for $[C_{23}H_{30}NO_3]^+$ requires *m/z* 368.2220, found 368.2223.

Synthesis of 39

A Radley carousel tube was charged with a magnetic stirrer bar. The tube was flame dried and then allowed to cool to room temperature under an inert gas using a Schlenk line. (*E*)-2-*tert*-Butoxy-*N*-(*p*-tolylmethylene)phenylamine **29** (69.5 mg, 0.26 mmol) and (S)-**21** (2.1 mg, 0.0026 mmol, 1 mol%) were loaded into the reaction tube. Three vacuum / nitrogen cycles were applied using a Schlenk line and then anhydrous chloroform (1 mL) was injected and the reaction placed in a cooling bath at −60 °C. A flux of an inert gas was admitted from the Schlenk line into the reaction whilst it cooled. The solution was stirred for one minute and then *tert*-butyl diazoacetate (0.038 mL, 0.28 mmol, 1.1 eq^{n}) was added *via* syringe. The solution was stirred at −60 °C for a further 12 hours after which Subsequent LC-MS analysis indicated the starting material imine had been completely consumed. Chloroform was removed *in vacuo* and the resulting residue was purified *via* flash chromatography on silica gel (elution with hexane / diethyl ether / dichloromethane : 92 / 6 / 2). A sample was submitted to chiral column analytical HPLC analysis [Chiralpak AD, *iso*-hexane / *iso*-propanol : 95 / 5, 1 mL / min, 3.9 min (1st peak), 6.8 min (2nd peak)]. The reaction afforded **39** as a transparent oil in 88% yield and 79% *e.e.*

Characterization of chiral non-racemic **39**.

^{1}H NMR ($CDCl_3$, 400 MHz) 7.33 (d, *J* = 8.06 Hz, 2H), 7.04 (d, *J* = 8.17 Hz, 1H), 6.97-6.92 (m, 1H), 6.88-6.85 (m, 3H), 3.38 (d, *J* = 6.74 Hz, 1H, C_2-*H*), 2.94 (d, *J* = 6.75 Hz, 1H, C_3-*H*), 2.26 (s, 3H, ArC*H₃*), 1.31 (s, 9H, $OC(CH_3)_3$), 1.14 (s, 9H, $OC(CH_3)_3$); ^{13}C NMR ($CDCl_3$, 75 MHz) 167.4, 148.1, 146.9, 137.2, 132.3, 128.6, 128.1, 123.4, 123.3, 123.0, 121.1, 81.4, 80.4, 47.6, 47.5, 28.8, 28.1, 21.4; $[\alpha]_D^{21}$ = −34.9 (c = 1.62, $CHCl_3$), FT-IR (thin film, cm^{-1}): 2977, 2929, 1746 (CO), 1716, 1592, 1489, 1450, 1392, 1366, 1308, 1261, 1223, 1160, 1111, 1047, 887, 809, 752; MS $(EI)^+$: *m/z* 382.2 $[M+H]^+$, 404.2 $[M+Na]^+$, 785.3 $[2M+Na]^+$; HRMS $(EI)^+$: exact mass calculated for $[C_{24}H_{32}NO_3]^+$ requires *m/z* 382.2377, found *m/z* 382.2377.

Synthesis of 40

A Radley carousel tube was charged with a magnetic stirrer bar, flame dried and then cooled to room temperature under an inert gas using a Schlenk line. (*E*)-2-*tert*-Butoxy-*N*-(4-cyanophenylmethylene)phenylamine **30** (72.5 mg, 0.26 mmol) and (S)-**21** (2.1 mg, 0.0026 mmol, 1 mol%) were loaded and three vacuum / nitrogen cycles were applied using a Schlenk line. Anhydrous chloroform (1 mL) was injected into the carousel tube which was then placed in a cooling bath at −60 °C. A flux of an inert gas was allowed from the Schlenk line into the reaction tube until the cooling had taken place. The solution was stirred for one minute after which *tert*-butyl diazoacetate (0.038 mL, 0.28 mmol, 1.1 eq^n) was added *via* syringe whilst the solution was stirred at −60 °C for a further 12 hours. Subsequent LC-MS analysis indicated that the starting material (imine) had been completely consumed. Chloroform was removed *in vacuo* and the residue was purified *via* flash chromatography on silica gel (elution with hexane / diethyl ether : 80 / 20). A sample was submitted to chiral column analytical HPLC analysis [Chiralpak AD, *iso*-hexane / *iso*-propanol : 95 / 5, 1 mL / min, 6.0 min (1st peak), 8.6 min (2nd peak)]. The reaction afforded **40** as a pale yellow solid in 98% yield and 97% *e.e.*

Characterization of chiral non-racemic **40**.

M.p. 156.5 - 158.3 °C, ^{1}H NMR ($CDCl_3$, 400 MHz) 7.59 (d, *J* = 8.46 Hz, 2H), 7.55 (d, *J* = 8.54 Hz, 2H), 6.97-6.94 (m, 1H), 6.91-6.84 (m, 3H), 3.42 (d, *J* = 6.77 Hz, 1H, C_2-*H*), 3.04 (d, *J* = 6.77 Hz, 1H, C_3-*H*), 1.28 (s, 9H, OC(CH_3)$_3$), 1.14 (s, 9H, OC(CH_3)$_3$); ^{13}C NMR ($CDCl_3$, 75 MHz) 166.5, 148.1, 145.8, 141.1, 131.7, 129.0, 123.6, 123.3, 123.2, 120.9, 119.1, 111.4, 81.9, 80.6, 47.8, 47.0, 28.8, 27.9; $[\alpha]_D^{21}$ = −68.2 (c = 2.59, $CHCl_3$), FT-IR (thin film, cm^{-1}): 2978, 2933, 2228, 1743, 1715, 1610, 1593, 1490, 1451, 1392, 1367, 1309, 1262, 1225, 1160, 1112, 1048, 887, 850, 753; MS (EI)$^+$: *m/z* 393.1 $[M+H]^+$, 415.1 $[M+Na]^+$, 807.3 $[2M+Na]^+$; HRMS (EI)$^+$: exact mass calculated for $[C_{24}H_{28}N_2O_3Na]^+$ requires *m/z* 415.1992, found *m/z* 415.1995.

Synthesis of 41

A Radley carousel tube was charged with a magnetic stirrer bar and flame dried. It was then cooled to room temperature under an inert gas using a Schlenk line. (*E*)-2-*tert*-Butoxy-*N*-(4-nitrophenylmethylene)phenylamine **31** (78 mg, 0.26 mmol) and (S)-**21** (2.1 mg, 0.0026 mmol, 1 mol%) were loaded into the reaction tube and three vacuum / nitrogen cycles were applied using a Schlenk line. Anhydrous chloroform (1 mL) was injected and the reaction tube was placed in a cooling bath at −60 °C. A flux of an inert gas was introduced from the Schlenk line whilst the reaction tube cooled. The solution was stirred for one minute whilst *tert*-butyl diazoacetate (0.038 mL, 0.28 mmol, 1.1 eq^n) was added *via* syringe. The solution was stirred at −60 °C for a further 12 hours. Subsequent LC-MS analysis indicated that starting material (imine) had been completely consumed. Chloroform was removed *in vacuo* and the resulting residue was purified *via* flash chromatography on silica gel (elution with hexane / diethyl ether : 80 / 20). A sample was submitted to chiral column analytical HPLC analysis [Chiralpak AD, *iso*-hexane / *iso*-propanol : 95 / 5, 1 mL / min, 6.3 min (1st peak), 10.5 min (2nd peak)]. **41** was a yellow solid in 98% yield and 98% *e.e.*

Characterization of chiral non-racemic **41**.

M.p. 117.4 - 119.3 °C, ^{1}H NMR ($CDCl_3$, 400 MHz) 8.17 (d, *J* = 8.75 Hz, 2H), 7.69 (d, *J* = 8.88 Hz, 2H), 7.02-6.98 (m, 1H), 6.95-6.90 (m, 3H), 3.50 (d, *J* = 6.76 Hz, 1H, C_2-*H*), 3.11 (d, *J* = 6.77 Hz, 1H, C_3-*H*), 1.32 (s, 9H, $OC(CH_3)_3$), 1.19 (s, 9H, $OC(CH_3)_3$); ^{13}C NMR ($CDCl_3$, 75 MHz) 166.5, 148.2, 147.6, 145.6, 143.2, 129.2, 123.7, 123.3, 123.2, 123.1, 120.9, 82.1, 80.6, 47.9, 46.9, 28.9, 28.0; $[\alpha]_D^{21}$ = −63.9 (c = 3.5, $CHCl_3$), FT-IR (thin film, cm^{-1}): 2978, 2933, 1743, 1716, 1603, 1520, 1490, 1451, 1392, 1367, 1344, 1262, 1224, 1160, 1111, 1048, 888, 854, 753; MS $(EI)^+$: *m/z* 412.1 $[M]^+$; HRMS $(EI)^+$: exact mass calculated for $[C_{23}H_{28}N_2O_5]^+$ requires *m/z* 412.1993, found *m/z* 412.1998.

Synthesis of 42

A Radley carousel tube was charged with a magnetic stirrer bar and flame dried and the allowed to cool to room temperature under an inert gas using a Schlenk line. The starting material imine **32** (125.3 mg, 0.26 mmol) and (S)-**21** (2.1 mg, 0.0026 mmol, 1 mol%) was loaded into the reaction tube and three vacuum / nitrogen cycles were applied using a Schlenk line. Anhydrous chloroform (1 mL) was injected into the carousel tube which was then placed in a cooling bath at −60 °C. A flux of an inert gas was administered from the Schlenk line whilst the reaction cooled. The solution was stirred for one minute after which *tert*-butyl diazoacetate (0.038 mL, 0.28 mmol, 1.1 eq^n) was added *via* syringe, the solution was stirred for a further 12 hours at −60 °C. Subsequent LC-MS analysis indicated the starting material imine had been completely consumed. The chloroform was removed *in vacuo* and the resulting residue purified *via* flash chromatography on silica gel (elution with hexane / diethyl ether : 8 / 1). A sample was submitted to chiral column analytical HPLC analysis [Chiralpak AD, *iso*-hexane / *iso*-propanol : 95 / 5, 1 mL / min, 8.3 min (1st peak), 27.5 min (2nd peak)]. The reaction afforded **42** as a yellow oil in 91% yield and 90% *e.e.*

Characterization of chiral non-racemic **42**.

^{1}H NMR ($CDCl_3$, 400 MHz) 7.69 (d, J = 7.52 Hz, 2H), 7.57 (d, J = 7.47 Hz, 2H), 7.48 (d, J = 8.69 Hz, 2H), 7.34 (t, J = 7.48 Hz, 2H), 7.25 (t, J = 7.39 Hz, 2H), 7.07 (d, J = 8.54 Hz, 2H), 6.95-6.92 (m, 1H), 6.87-6.84 (m, 3H), 4.44 (d, J = 7.27 Hz, 2H, ArCHCH_2), 4.24 (t, J = 7.28 Hz, 1H, ArCHCH$_2$O-), 3.39 (d, J = 6.72 Hz, 1H, C_2-H), 2.97 (d, J = 6.73 Hz, 1H, C_3-H), 1.29 (s, 9H, OC(CH_3)$_3$), 1.13 (s, 9H, OC(CH_3)$_3$); ^{13}C NMR ($CDCl_3$, 75 MHz) 167.2, 153.8, 150.7, 148.1, 146.5, 143.4, 143.3, 141.5, 133.5, 129.4, 128.2, 127.5, 125.4, 123.4, 123.3, 121.1, 120.7, 120.3, 81.7, 80.6, 70.6, 47.6, 47.0, 46.9, 28.9, 28.0; $[\alpha]_D^{21}$ = −19.1 (c = 5.5, $CHCl_3$), FT-IR (thin film, cm^{-1}): 2977, 2931, 1762, 1714, 1610, 1592, 1509, 1490, 1450, 1391, 1367, 1300, 1259, 1206, 1163, 1111, 1048, 970, 888, 847, 758, 741; MS $(EI)^+$: *m/z* 606.2 $[M+H]^+$, 628.2 $[M+Na]^+$; HRMS $(EI)^+$: exact mass calculated for $[C_{38}H_{39}NO_6NH_4]^+$ requires *m/z* 623.3116, found *m/z* 623.3118.

Synthesis of 43

A Radley carousel tube was charged with a magnetic stirrer bar, flame dried and then cooled to room temperature under an inert gas using a Schlenk line. (*E*)-2-*tert*-Butoxy-*N*-(4-fluorophenylmethylene)phenylamine **33** (70.5 mg, 0.26 mmol) and (S)-**21** (2.1 mg, 0.0026 mmol, 1 mol%) were loaded into the reaction tube whilst three vacuum / nitrogen cycles were applied using the Schlenk line. Anhydrous chloroform (1 mL) was injected into the carousel and the reaction placed in a cooling bath at −60 °C. A flux of an inert gas was administered whilst being stirred for one minute and the *tert*-butyl diazoacetate (0.038 mL, 0.28 mmol, 1.1 eq^n) added *via* syringe. The solution was stirred at −60 °C for a further 12 hours. Subsequent LC-MS analysis indicated that the starting material imine had been completely consumed. Chloroform was removed *in vacuo* and the resulting residue was purified *via* flash chromatography on silica gel (elution with hexane / diethyl ether / dichloromethane : 92 / 4 / 2). A sample of **43** was submitted to chiral column analytical HPLC analysis [Chiralpak AD, *iso*-hexane / *iso*-propanol : 95 / 5, 1 mL / min, 3.8 min (1st peak), 6.1 min (2nd peak)]. The reaction afforded **43** as a transparent oil in 97% yield and 80% *e.e.*

Characterization of chiral non-racemic **43**.

^{1}H NMR ($CDCl_3$, 400 MHz) 7.45-7.41 (m, 2H), 6.99-6.92 (m, 3H), 6.89-6.84 (m, 3H), 3.38 (d, *J* = 6.70 Hz, 1H, C_2-*H*), 2.96 (d, *J* = 6.71 Hz, 1H, C_3-*H*), 1.29 (s, 9H, OC(CH_3)$_3$), 1.15 (s, 9H, OC(CH_3)$_3$); ^{13}C NMR ($CDCl_3$, 75 MHz) 167.1, 163.7, 161.3, 148.1, 146.5, 131.1, 129.8, 123.3, 121.1, 114.9, 114.7, 81.6, 80.5, 47.5, 47.0, 28.8, 28.0; $[\alpha]_D^{21}$ = −23.6 (c = 2.18, $CHCl_3$), FT-IR (thin film, cm^{-1}): 2978, 2933, 1744 (CO), 1715, 1607, 1593, 1512, 1490, 1450, 1392, 1367, 1304, 1262, 1220, 1162, 1120, 1048, 887, 846, 752; MS $(EI)^+$: *m/z* 386.2 $[M+H]^+$, 408.1 $[M+Na]^+$, 793.3 $[2M+Na]^+$; HRMS $(EI)^+$: exact mass calculated for $[C_{23}H_{28}FNO_3Na]^+$ requires *m/z* 408.1945, found *m/z* 408.1950.

Synthesis of 44

A Radley carousel tube was charged with a magnetic stirrer bar. The tube was flame dried and then cooled to room temperature under an inert gas using a Schlenk line. (*E*)-2-*tert*-Butoxy-*N*-(4-chlorophenylmethylene)phenylamine **34** (75 mg, 0.26 mmol) and (S)-**21** (2.1 mg, 0.0026 mmol, 1 mol%) was loaded into the reaction tube and three vacuum / nitrogen cycles were applied. Anhydrous chloroform (1 mL) was injected and the reaction tube was then placed in the cooling bath at -60 °C. A flux of an inert gas was allowed from the Schlenk line into the reaction tube until the cooling had taken place. The solution was stirred for a further one minute before *tert*-butyl diazoacetate (0.038 mL, 0.28 mmol, 1.1 eq^{n}) was added *via* syringe. The solution was stirred at -60 °C for a further 12 hours. Subsequent LC-MS analysis indicated that the starting material (imine) had been completely consumed. Chloroform was removed *in vacuo* and the resulting residue was purified *via* flash chromatography on silica gel (elution with hexane / ether : 80 / 20). A sample of **44** was submitted to chiral column analytical HPLC analysis [Chiralpak AD, *iso*-hexane / *iso*-propanol : 95 / 5, 1 mL / min, 3.9 min (1st peak), 6.4 min (2nd peak)]. The reaction afforded **44** as a white solid in 97% yield and 93% *e.e.*

Characterization of chiral non-racemic **44**.

M.p. 68.2 – 70.2 °C; ^{1}H NMR ($CDCl_3$, 400 MHz) 7.40 (d, J = 8.63 Hz, 2H), 7.25 (d, J = 8.60 Hz, 2H), 6.96-6.93 (m, 1H), 6.88-6.87 (m, 3H), 3.36 (d, J = 6.73 Hz, 1H, C_2-H), 2.97 (d, J = 6.75 Hz, 1H, C_3-H), 1.29 (s, 9H, $OC(CH_3)_3$), 1.16 (s, 9H, $OC(CH_3)_3$); ^{13}C NMR ($CDCl_3$, 75 MHz) 165.8, 146.9, 145.2, 132.8, 132.2, 128.4, 126.8, 122.1, 122.05, 122.0, 119.8, 80.5, 79.3, 46.3, 45.8, 27.7, 26.8; $[\alpha]_D^{21}$ = –37.7 (c = 1.24, $CHCl_3$), IR (thin film, cm^{-1}): 2977, 2923, 1745, 1716, 1593, 1489, 1450, 1392, 1367, 1261, 1223, 1162, 1112, 1088, 1048, 887, 844, 769, 752; MS $(EI)^+$: *m/z* 402.2 $[M+H]^+$, 424.1 $[M+Na]^+$, 825.2 $[2M+Na]^+$; HRMS $(EI)^+$: exact mass calculated for $[C_{23}H_{28}ClNO_3H]^+$ requires *m/z* 402.1830, found *m/z* 402.1832.

Synthesis of 45

A Radley carousel tube was charged with a magnetic stirrer bar, flame dried and then cooled to room temperature under an inert gas using a Schlenk line. (*E*)-2-*tert*-Butoxy-*N*-(4-bromophenylmethylene)phenylamine **35** (86.5 mg, 0.26 mmol) and (S)-**21** (2.1 mg, 0.0026 mmol, 1 mol%) were loaded and three vacuum / nitrogen cycles were applied. Anhydrous chloroform (1 mL) was injected and the reaction tube was cooled in a bath at −60 °C. A flux of an inert gas was administered from the Schlenk line and the solution was stirred for one minute whilst *tert*-butyl diazoacetate (0.038 mL, 0.28 mmol, 1.1 eq^n) was added *via* syringe. The solution was stirred at −60 °C for a further 12 hours. Subsequent LC-MS analysis indicated that the starting material (imine) had been completely consumed. Chloroform was removed and the resulting residue purified *via* flash chromatography on silica gel (elution with hexane / ether : 80 / 20). A sample of **45** was submitted to chiral column analytical HPLC analysis [Chiralpak AD, *iso*-hexane / *iso*-propanol : 95 / 5, 1 mL / min, 4.0 min (1st peak), 6.7 min (2nd peak)]. The reaction afforded **45** as a white solid in 96% yield and 93% *e.e.*

Characterization of chiral non-racemic **45**.

M.p. 95.3- 97.1 °C; ^{1}H NMR ($CDCl_3$, 400 MHz) 7.43 (d, J = 8.63 Hz, 2H), 7.38 (d, J = 8.48 Hz, 2H), 7.00-6.97 (m, 1H), 6.93-6.90 (m, 3H), 3.39 (d, J = 6.74 Hz, 1H, C_2-*H*), 3.02 (d, J = 6.74 Hz, 1H, C_3-*H*), 1.34 (s, 9H, $OC(CH_3)_3$), 1.20 (s, 9H, $OC(CH_3)_3$); ^{13}C NMR ($CDCl_3$, 75 MHz) 167.0, 148.1, 146.4, 134.6, 131.0, 129.9, 123.4, 123.3, 123.2, 121.5, 121.0, 81.7, 80.5, 47.5, 47.0, 28.9, 28.0; $[\alpha]_D^{21}$ = −39.1 (c = 2.63, $CHCl_3$), IR (thin film, cm^{-1}): 2977, 2932, 1745, 1716, 1594, 1489, 1450, 1392, 1367, 1262, 1222, 1160, 1112, 1012, 887, 843, 770, 752; MS $(EI)^+$: *m/z* 446.0 $[M]^+$, 468.1 $[M+Na]^+$, 915.1 $[2M+Na]^+$; HRMS $(EI)^+$: exact mass calculated for $[C_{23}H_{28}{}^{81}BrNO_3]^+$ requires *m/z* 448.1305, found *m/z* 448.1301.

Synthesis of 46

A Radley carousel tube was charged with a magnetic stirrer bar, flame dried and then cooled to room temperature under an inert gas using a Schlenk line. (*E*)-2-*tert*-Butoxy-*N*-(4-iodophenylmethylene)phenylamine **36** (99 mg, 0.26 mmol) and (S)-21 (2.1 mg, 0.0026 mmol, 1 mol%) were loaded into the reaction tube and three vacuum / nitrogen cycles were applied using a Schlenk line. Anhydrous chloroform (1 mL) was injected and the reaction was placed in the cooling bath at −60 °C. A flux of an inert gas was admitted from the Schlenk line whilst the reaction tube cooled after which the solution was stirred for one minute. *tert*-Butyl diazoacetate (0.038 mL, 0.28 mmol, 1.1 eq^n) was added *via* syringe and the solution stirred at −60 °C for a further 12 hours. Subsequent LC-MS analysis indicated the starting imine **36** had been completely consumed. Chloroform was removed *in vacuo* and the resulting residue purified *via* flash chromatography on silica gel (elution with hexane / diethyl ether : 80 / 20). A sample of **46** was submitted to chiral column analytical HPLC analysis [Chiralpak AD, *iso*-hexane / *iso*-propanol : 95 / 5, 1 mL / min, 4.6 min (1st peak), 9.2 min (2nd peak)]. The reaction afforded **46** as a yellow solid in 96% yield and 92% *e.e.*

Characterization of chiral non-racemic **46**.

M.p. 122.3- 124.2 °C; ^{1}H NMR ($CDCl_3$, 400 MHz) 7.58 (d, J = 8.47 Hz, 2H), 7.21 (d, J = 8.26 Hz, 2H), 6.95-6.92 (m, 1H), 6.88-6.85 (m, 3H), 3.33 (d, J = 6.73 Hz, 1H, C_2-*H*), 2.97 (d, J = 6.75 Hz, 1H, C_3-*H*), 1.29 (s, 9H, $OC(CH_3)_3$), 1.16 (s, 9H, $OC(CH_3)_3$); ^{13}C NMR ($CDCl_3$, 75 MHz) 166.9, 148.1, 146.3, 136.9, 135.3, 130.2, 123.4, 123.3, 123.25, 121.1, 93.1, 81.7, 80.6, 47.5, 47.1, 28.9, 28.0; $[\alpha]_D^{21}$ = −35.9 (c = 2.45, $CHCl_3$), FT-IR (thin film, cm^{-1}): 2977, 2930, 1744, 1715, 1593, 1489, 1450, 1392, 1367, 1261, 1222, 1162, 1112, 1047, 1007, 887, 842, 808, 751; MS $(EI)^+$: *m/z* 494.1 $[M+H]^+$, 516.2 $[M+Na]^+$; HRMS $(EI)^+$: exact mass calculated for $[C_{23}H_{29}INO_3]^+$ requires *m/z* 494.1187, found *m/z* 494.1182.

Synthesis of 47

A Radley carousel tube was charged with a magnetic stirrer bar, flame dried and then cooled to room temperature under an inert gas using a Schlenk line. (*E*)-2-*tert*-Butoxy-*N*-(2-chlorophenylmethylene)phenylamine **37** (75 mg, 0.26 mmol) and (S)-**21** (2.1 mg, 0.0026 mmol, 1 mol%) were loaded and three vacuum / nitrogen cycles were applied using a Schlenk line. Anhydrous chloroform (1 mL) was injected into the tube which was then placed in the cooling bath at −60 °C. A flux of an inert gas was admitted from the Schlenk line until cooling had taken place. The solution was stirred for one minute and then *tert*-butyl diazoacetate (0.038 mL, 0.28 mmol, 1.1 eq^n) was added *via* syringe, the solution was stirred at −60 °C for a further 12 hours. Subsequent LC-MS analysis indicated that starting imine **37** had been completely consumed. Chloroform was removed *in vacuo* and the resulting residue purified *via* flash chromatography on silica gel (elution with hexane / diethyl ether / dichloromethane : 96 / 2 / 2). A sample of **47** was submitted to chiral column analytical HPLC analysis [Chiralpak AD, *iso*-hexane / *iso*-propanol : 95 / 5, 1 mL / min, 3.9 min (1st peak), 7.3 min (2nd peak)]. The reaction afforded **47** as a white solid in 95% yield and 79% *e.e.*

Characterization of chiral non-racemic **47**

M.p. 70.2 – 72.4 °C, ^{1}H NMR ($CDCl_3$, 400 MHz) 7.76-7.23 (m, 1H), 7.35-7.32 (m, 1H), 7.27-7.24 (m, 2H), 7.05-6.98 (m, 1H), 6.96-6.95 (m, 3H), 3.64 (d, *J* = 6.68 Hz, 1H, C_2-*H*), 3.15 (d, *J* = 6.68 Hz, 1H, C_3-*H*), 1.41 (s, 9H, OC(CH_3)$_3$), 1.19 (s, 9H, OC(CH_3)$_3$); ^{13}C NMR ($CDCl_3$, 75 MHz) 167.1, 148.3, 146.2, 133.8, 133.6, 130.9, 128.9, 128.6, 126.4, 123.3, 123.1, 122.8, 120.9, 81.3, 80.4, 46.8, 46.1, 28.9, 27.9; $[\alpha]_D^{21}$ = −78.6 (c = 0.35, $CHCl_3$), FT-IR (thin film, cm^{-1}): 2976, 2927, 2855, 1746, 1721, 1593, 1491, 1449, 1392, 1366, 1261, 1223, 1164, 1111, 1046, 1032, 888, 850, 798, 751; MS $(EI)^+$: *m/z* 402.1 $[M+H]^+$, 424.1 $[M+Na]^+$, 825.2 $[2M+Na]^+$; HRMS $(EI)^+$: exact mass calculated for $[C_{23}H_{28}ClNO_3Na]^+$ requires *m/z* 424.1650, found *m/z* 424.1647.

Three component one pot asymmetric synthesis of 40

4-Cyanobenzaldehyde (34 mg, 0.26 mmol,), *O-tert*-butoxy aniline (43 mg, 0.26 mmol), and catalyst (S)-**21** (10.8 mg, 0.013 mmol, 5%) were added to a flame dried biotage 2 mL microwave vial under nitrogen. 1 mL of chloroform was added (pre-dried over 4Å molecular sieves), followed by ~40 mg of powdered 4Å molecular sieves and the vial sealed with a PTFE crimp cap. After stirring at room temperature for 6 h, the reaction was cooled to -60 °C. After 30 minutes, *tert*-butyl diazoacetate (40 μL, 0.286 mmol) was added *via* syringe, and the reaction mixture was stirred at -60 °C, monitoring by ^{1}H-NMR until the reaction was deemed complete. At this point the reaction mixture was passed through a short plug of silica, eluting with diethyl ether. The solvents were removed under reduced pressure, and the residue was purified by flash chromatography (14 % diethyl ether in petroleum ether). A sample was submitted to chiral column analytical HPLC analysis [Chiralpak AD, *iso*-hexane / *iso*-propanol : 95/ 5, 1 mL / min, 7.04 min (1st peak), 13.69 min (2nd peak), 99% *e.e.*]. The reaction product **40** was a pale yellow solid afforded in a 74 % yield.

Characterization of the chiral non-racemic product **40**

This material had, within experiemental error, identical physicochemical characteristics as the material prepared previously via pre-imine synthesis.

Three component one pot asymmetric synthesis of 38

Pyridine-2-carboxaldehyde (25 μL, 0.26 mmol,), *O-tert*-butoxy aniline (43 mg, 0.26 mmol), and catalyst (S)-**21** (2.2 mg, 0.0026 mmol, 1%) were added to a flame dried biotage 2 mL microwave vial under nitrogen. 1 mL of chloroform was added (pre-dried over 4Å molecular sieves), followed by ~40 mg of powdered 4Å molecular sieves and the vial sealed with a PTFE crimp cap. After stirring at room temperature for 6 h, the reaction was cooled to -60 °C. After 30 minutes, *tert*-butyl diazoacetate (40 μL, 0.286 mmol) was added *via* syringe, and the reaction mixture was stirred at -60 °C, monitoring by ^{1}H-NMR until the reaction was deemed complete. At this point the reaction mixture was passed through a short plug of silica, eluting with diethyl ether. The solvents were removed under reduced pressure, and the residue was purified by flash chromatography (14 % diethyl ether in petroleum ether). A sample was submitted to chiral column analytical HPLC analysis [Chiralpak AD, *iso*-hexane / *iso*-propanol : 8 / 2, 1 mL / min, 5.25 min (1st peak), 7.43 min (2nd peak), 96% *e.e.*]. The reaction product **38** was a colourless oil afforded in a 96 % yield.

Characterization of the chiral non-racemic product **38**

This material had, within experiemental error, identical physicochemical characteristics as the material prepared previously via pre-imine synthesis.

Three component one pot asymmetric synthesis of 49

4-(Methylthio)benzaldehyde (51 mg, 0.26 mmol,), *O-tert*-butoxy aniline (43 mg, 0.26 mmol), and catalyst (S)-**21** (10.8 mg, 0.013 mmol, 5%) were added to a flame dried biotage 2 mL microwave vial under nitrogen. 1 mL of chloroform was added (pre-dried over 4Å molecular sieves), followed by ~40 mg of powdered 4Å molecular sieves and the vial sealed with a PTFE crimp cap. After stirring at room temperature for 6 h, the reaction was cooled to -60 °C. After 30 minutes, *tert*-butyl diazoacetate (40 μL, 0.286 mmol) was added *via* syringe, and the reaction mixture was stirred at -60 °C, monitoring by ^{1}H-NMR until the reaction was deemed complete. At this point the reaction mixture was passed through a short plug of silica, eluting with diethyl ether. The solvents were removed under reduced pressure, and the residue was purified by flash chromatography (12 % diethyl ether in petroleum ether). A sample was submitted to chiral column analytical HPLC analysis [Chiralpak AD, *iso*-hexane / *iso*-propanol : 95/ 5, 1 mL / min, 5.06 min (1st peak), 9.63 min (2nd peak), 76 % *e.e.*]. The reaction product was a colourless oil **49** afforded in a 72 % yield.

Characterization of the chiral non-racemic product **49**

^{1}H NMR ($CDCl_3$, 400 MHz) δ 7.43 (d, 2H, *J* = 8.47 Hz, ArH), 7.21 (d, 2H, *J* = 8.26 Hz, Ar-H), 7.03-6.87 (m, 4H, ArH), 3.42 (d, *J* = 6.73 Hz, 1H, C_2-H), 3.01 (d, 1H, *J* = 6.72 Hz, C_3-H), 2.44 (s, 3H, SCH_3), 1.36 (s, 9H, $C(CH_3)_3$), 1.19 (s, 9H, $C(CH_3)_3$); ^{13}C NMR ($CDCl_3$, 100 MHz) 167.2, 148.1, 146.7, 137.6, 132.5, 128.7, 126.3, 123.4, 123.3, 123.2, 121.1, 81.5, 80.6, 47.6, 47.3, 28.9, 28.1, 16.3 ppm; $[\alpha]_D^{23}$ -35.6 (c 1.4 $CHCl_3$); FT-IR (thin film cm^{-1}) 2976, 2929, 1743, 1711, 1592, 1489. 1391, 1365, 1304, 1260; MS (ES) 414.2 $[M+H]^+$, 436.2 $[M+Na]^+$; HRMS (EI) Exact mass calculated for $[C_{24}H_{32}NO_3S]$ requires 414.2097 found 414.2099

Three component one pot asymmetric synthesis of 50

4-(Trifluoromethyl)benzaldehyde (17 μL, 0.13 mmol,), *O-tert*-butoxy aniline (43 mg, 0.13 mmol), and catalyst (S)-**21** (10.8 mg, 0.0065 mmol, 5%) were added to a flame dried biotage 2 mL microwave vial under nitrogen. 1 mL of chloroform was added (pre-dried over 4Å molecular sieves), followed by ~40 mg of powdered 4Å molecular sieves and the vial sealed with a PTFE crimp cap. After stirring at room temperature for 6 h, the reaction was cooled to -60 °C. After 30 minutes, *tert*-butyl diazoacetate (20 μL, 0.143 mmol) was added *via* syringe, and the reaction mixture was stirred at -60 °C, monitoring by ^{1}H-NMR until the reaction was deemed complete. At this point the reaction mixture was passed through a short plug of silica, eluting with diethyl ether. The solvents were removed under reduced pressure, and the residue was purified by flash chromatography (12 % diethyl ether in petrolum ether). A sample was submitted to chiral column analytical HPLC analysis [Chiralpak AD, *iso*-hexane / *iso*-propanol : 95/ 5, 1 mL / min, 3.98 min (1st peak), 6.06 min (2nd peak), 90% *e.e.*]. The reaction product **50** was a colourless oil afforded in a 83 % yield.

Characterization of the chiral non-racemic product **50**

^{1}H NMR ($CDCl_3$, 400 MHz) δ 7.64 (d, 2H, J = 8.09 Hz, ArH), 7.57 (d, 2H, J = 8.48 Hz, Ar-H), 7.06-6.90 (m, 4H, ArH), 3.49 (d, J = 6.78 Hz, 1H, C_2-H), 3.07 (d, 1H, J = 6.77 Hz, C_3-H) 1.37 (s, 9H, $C(CH_3)_3$), 1.19 (s, 9H, $C(CH_3)_3$); ^{13}C NMR ($CDCl_3$, 100 MHz) 166.8, 148.2, 146.1, 139.7, 130.1, 129.7, 129.4, 128.6, 125.8, 124.9, 124.8, 124.8, 124.8, 123.5, 123.3, 123.2, 123.1, 121.0, 81.8, 80.6, 47.6, 47.0, 28.9, 28.0 ppm; $[\alpha]_D^{23}$ -41.0 (c 1 $CHCl_3$); FT-IR (thin film cm^{-1}) 2978, 1844, 1715, 1620, 1593, 1489, 1450, 1392, 1323, 1280, 1159; MS (ES) 458.1 $[M+Na]^+$; HRMS (EI) Exact mass calculated for $[C_{24}H_{29}F_3NO_3]$ requires 436.2094 found 436.2094

Three component one pot asymmetric synthesis of the ester precursor to 51

Pentafluorobenzaldehyde (51 mg, 0.26 mmol,), *O-tert*-butoxy aniline (43 mg, 0.26 mmol), and catalyst (S)-**21** (2.2 mg, 0.0026 mmol, 1%) were added to a flame dried biotage 2 mL microwave vial under nitrogen. 1 mL of chloroform was added (pre-dried over 4Å molecular sieves), followed by ~40 mg of powdered 4Å molecular sieves and the vial sealed with a PTFE crimp cap. After stirring at room temperature for 6 h, the reaction was cooled to -60 °C. After 30 minutes, *tert*-butyl diazoacetate (40 μL, 0.286 mmol) was added *via* syringe, and the reaction mixture was stirred at -60 °C, monitoring by ^{1}H-NMR until the reaction was deemed complete. At this point the reaction mixture was passed through a short plug of silica, eluting with diethyl ether. The solvents were removed under reduced pressure, and the residue was purified by flash chromatography (12 % diethyl ether in petroleum ether). The reaction product afforded the ester as a colourless oil in a 74 % yield.

Characterization of the chiral non-racemic ester precursor to **51**

^{1}H NMR ($CDCl_3$, 400 MHz) δ 7.14-6.88 (m, 4H, ArH), 3.33 (d, 1H, *J* = 5.57 Hz, C_2-H), 3.04 (d, 1H, J = 5.97 Hz, C_3-H), 1.40 (s, 9H, $C(CH_3)_3$), 1.38 (s, 9H, $C(CH_3)_3$); ^{13}C NMR ($CDCl_3$, 100 MHz) 167.2, 148.2, 147.7, 147.7, 147.5, 145.3, 144,5, 144.3, 139.3, 139.1, 139.0, 136.0, 135.8, 135.8, 123.7, 123.1, 122.8, 120.7, 110.5, 110.2, 110.2, 82.1, 80.5, 43.55, 36.82, 28.6, 27.6 ppm; $[\alpha]_D^{23}$ -122.7 (c 0.8 $CHCl_3$); FT-IR (thin film cm^{-1}) 2979, 2933, 1740, 1655, 1594, 1523, 1500, 1451, 1393, 1369, 1331; MS (ES) 480.1 $[M+Na]^+$; HRMS (EI) Exact mass calculated for $[C_{23}H_{25}F_5NO_3]$ requires 458.1749 found 458.1748

Synthesis of compound 51

To a stirred solution of the ester generated in the previous aziridination (40 mg, 0.088 mmol) in 1 mL acetonitrile, in a Biotage 4 mL microwave vial, was added *para*-toluenesulfonic acid (19 mg, 0.096 mmol, 1.1 eq). 500 µL distilled water was added, the reaction was capped with a PTFE seal, and heated to 60 °C for 5 h. After this time the reaction was diluted with 10 mL ethyl acetate, washed with 15 mL saturated. $NaHCO_{3(aq)}$, 10 mL brine, dried with $MgSO_4$, filtered, and the solvent removed. The impure material was purified by flash chromatography (30 % diethyl ether in petroleum ether). A sample was submitted to chiral column analytical HPLC analysis [Chiralpak AD, *iso*-hexane / *iso*-propanol : 95/ 5, 1 mL / min, 2.67 min (1st peak), 3.00 min (2nd peak), 90% *e.e.*]. The reaction product **51** was a colourless oil afforded in a 74 % yield.

Characterization of the chiral non-racemic product **51**

^{1}H NMR ($CDCl_3$, 400 MHz) δ 7.13-7.05 (m, 1H, ArH), 6.97 (dd, 1H, *J* 8.00, 1.31 Hz, Ar-H), 6.91-6.81 (m, 2H, Ar-H), 6.51 (s, 1H, Ar-OH), 3.64 (d, 1H, *J* 6.19 Hz, C_2-H), 3.09 (d, 1H, J = 6.28 Hz, C_3-H), 1.40 (s, 9H, $C(CH_3)_3$); ^{13}C NMR ($CDCl_3$, 100 MHz) 166.4, 151.5, 147.7, 147.6, 147.5, 144.3, 144.3, 144.2, 139.4, 139.3, 139.2, 139.2, 136.1, 126.4, 120.4, 117.6, 115.7, 83.0, 44.5, 37.1, 27.6 ppm; $[\alpha]_D^{23}$ 17.2 (c 1 $CHCl_3$); FT-IR (thin film cm^{-1}) 3408, 1721, 1597, 1523, 1501, 1458, 1370, 1281, 1156; MS (ES) 424.1 $[M+Na]^+$; HRMS (EI) Exact mass calculated for $[C_{19}H_{17}F_5NO_3]$ requires 402.1123 found 402.1129

Three component one pot asymmetric synthesis of 52

Quinoline-2-carboxaldehyde (40.8 mg, 0.26 mmol,), *O-tert*-butoxy aniline (43 mg, 0.26 mmol), and catalyst (S)-**21** (2.2 mg, 0.0026 mmol, 1%) were added to a flame dried biotage 2 mL microwave vial under nitrogen. 1 mL of chloroform was added (pre-dried over 4Å molecular sieves), followed by ~40 mg of powdered 4Å molecular sieves and the vial sealed with a PTFE crimp cap. After stirring at room temperature for 6 h, the reaction was cooled to -60 °C. After 30 minutes, *tert*-butyl diazoacetate (40 μL, 0.286 mmol) was added *via* syringe, and the reaction mixture was stirred at -60 °C, monitoring by ^{1}H-NMR until the reaction was deemed complete. At this point the reaction mixture was passed through a short plug of silica, eluting with diethyl ether. The solvents were removed under reduced pressure, and the residue was purified by flash chromatography (14 % diethyl ether in petroleum ether). A sample was submitted to chiral column analytical HPLC analysis [Chiralpak AD, *iso*-hexane / *iso*-propanol : 8 / 2, 1 mL / min, 4.78 min (1st peak), 5.80 min (2nd peak), 98% *e.e.*]. The reaction product **52** was a colourless oil afforded in a 81 % yield.

Characterization of the chiral non-racemic product **52**

^{1}H NMR ($CDCl_3$, 400 MHz) δ 8.14 (d, 1H, *J* = 8.50 Hz, Ar-H), 8.07 (d, 1H, *J* = 8.49 Hz, Ar-H), 7.90 (d, 1H, *J* = 8.53 Hz, Ar-H), 7.82 (d, 1H, *J* = 8.08 Hz, Ar-H), 7.70 (t, 1H, *J* = 7.71, 7.71 Hz, Ar-H), 7.52 (t, 1H, *J* = 7.51, 7.51 Hz, Ar-H), 7.12-6.90 (m, 4H, ArH), 3.82 (d, 1H, *J* = 6.82 *Hz,* C_2-H), 3.21 (d, 1H, *J* = 6.84 *Hz*, C_3-H) 1.38 (s, 9H, $C(CH_3)_3$), 1.18 (s, 9H, $C(CH_3)_3$); ^{13}C NMR ($CDCl_3$, 100 MHz) 167.0, 156.7, 148.3, 147.7, 145.8, 135.9, 129.6, 128.9, 127.9, 127.8, 126.4, 123.5, 123.2, 122.9, 121.3, 121.1, 81.7, 80.5, 49.2, 47.4, 29.0, 28.0 ppm; $[\alpha]_D^{23}$ -78.2 (c 1.7 $CHCl_3$); FT-IR (thin film cm^{-1}) 2976, 2931, 1740, 1718, 1618, 1597, 1562, 1489, 1449, 1426, 1330, 1311, 1227; MS (ES) 419.2 $[M+H]^+$, 441.2 $[M+Na]^+$; HRMS (EI) Exact mass calculated for $[C_{26}H_{31}N_2O_3]$ requires 419.2329 found 419.2329

Three component one pot asymmetric synthesis of 53

2-Napthaldehyde (40.6 mg, 0.26 mmol,), *O-tert*-butoxy aniline (43 mg, 0.26 mmol), and catalyst (S)-**21** (10.8 mg, 0.013 mmol, 5%) were added to a flame dried biotage 2 mL microwave vial under nitrogen. 1 mL of chloroform was added (pre-dried over 4Å molecular sieves), followed by ~40 mg of powdered 4Å molecular sieves. The vial was sealed with a PTFE crimp cap. After stirring at room temperature for 6 h, the reaction was cooled to -60 °C. After 30 minutes, *tert*-butyl diazoacetate (40 µL, 0.286 mmol) was added *via* syringe, and the reaction mixture was stirred at -60 °C, monitoring by ^{1}H-NMR until the reaction was deemed complete. At this point the reaction mixture was passed through a short plug of silica, eluting with diethyl ether. The solvents were removed under reduced pressure, and the residue was purified by flash chromatography (14 % diethyl ether in petroleum ether). A sample was submitted to chiral column analytical HPLC analysis [Chiralpak AD, *iso*-hexane / *iso*-propanol : 95 / 5, 1 mL / min, 5.11 min (1st peak), 11.66 min (2nd peak), 82% *e.e.*]. The reaction product **53** was a colourless oil afforded in a 61 % yield.

Characterization of the chiral non-racemic product **53**

^{1}H NMR ($CDCl_3$, 300 MHz) δ 8.02 (s, 1H, ArH), 7.93-7.72 (m, 3H, Ar-H), 7.64 (dd, 1H, *J* = 1.61, 8.45 Hz, Ar-H), 7.58-7.35 (m, 2H, Ar-H) 7.18-6.85 (m, 4H, ArH), 3.64 (d, 1H, *J* = 6.73 Hz, C_2-H), 3.13 (d, 1H, *J* = 6.78 Hz, C_3-H), 1.39 (s, 9H, $C(CH_3)_3$), 1.15 (s, 9H, $C(CH_3)_3$); ^{13}C-NMR ($CDCl_3$, 75 MHz) 166.2, 146.9, 145.4, 131.9, 131.8, 126.9, 126.6, 126.3, 126.0, 124.9, 124.6, 122.0, 121.9, 121.9, 120.0, 80.3, 79.3, 46.7, 46.5, 27.7, 26.7 ppm; $[\alpha]_D^{26}$ = -25.6 (c 0.9 $CHCl_3$); FT-IR (thin film, cm^{-1}) 2977, 1740, 1708, 1591, 1449, 1413, 1392, 1367, 1261, 1160, 1111; MS $(EI)^+$: *m/z* 440.2 $[M+Na]^+$, 857.5 $[2M+Na]^+$; HRMS $(EI)^+$: exact mass calculated for $[C_{27}H_{32}NO_3]^+$ requires *m/z* 418.2377, found *m/z* 418.2377

Three component one pot asymmetric synthesis of 54

Terephthalaldehyde (34.8 mg, 0.26 mmol,), *O-tert*-butoxy aniline (43 mg, 0.26 mmol), and catalyst (S)-**21** (5.4 mg, 0.0065 mmol, 5%) were added to a flame dried biotage 2 mL microwave vial under nitrogen. 1 mL of chloroform was added (pre-dried over 4Å molecular sieves), followed by ~40 mg of powdered 4Å molecular sieves and the vial sealed with a PTFE crimp cap. After stirring at room temperature for 6 h, the reaction was cooled to -60 °C. After 30 minutes, *tert*-butyl diazoacetate (17.5 μL, 0.27 mmol) was added *via* syringe, and the reaction mixture was stirred at -60 °C, monitoring by ^{1}H-NMR until the reaction was deemed complete. At this point the reaction mixture was passed through a short plug of silica, eluting with diethyl ether. The solvents were removed under reduced pressure, and the residue was purified by flash chromatography (12 % diethyl ether in petroleum ether). A sample was submitted to chiral column analytical HPLC analysis [Chiralpak AD, *iso*-hexane / *iso*-propanol : 95/ 5, 1 mL / min, 6.94 min (1st peak), 14.85 min (2nd peak), 90 % *e.e.*]. The reaction product (**54**) was a colourless oil afforded in a 70 % yield.

Characterization of the chiral non-racemic product **54**

^{1}H NMR ($CDCl_3$, 400 MHz) δ 10.01 (s, 1H, CHO), 7.86 (d, 2H, *J* = 8.33 Hz, ArH), 7.71 (d, 2H, *J* = 8.15 Hz, ArH), 7.11-6.85 (m, 4H, ArH), 3.52 (d, *J* = 6.80 Hz, 1H, C_2-H), 3.12 (d, 1H, *J* = 6.80 Hz, C_3-H), 1.35 (s, 9H, $C(CH_3)_3$), 1.19 (s, 9H, $C(CH_3)_3$); ^{13}C NMR ($CDCl_3$, 100 MHz) 192.2, 166.6, 148.1, 145.9, 142.6, 135.8, 129.3, 128.8, 123.4, 123.1, 123.0, 120.9, 81.6, 80.3, 47.5, 47.0, 28.6, 27.7 ppm; $[\alpha]_D^{23}$ -67.4 (c 2.4 $CHCl_3$); FT-IR (thin film cm^{-1}) 2976. 2932, 1742, 1701, 1608, 1592, 1577, 1489, 1450, 1391, 1303, 1279; MS (ES) 418.3 $[M+Na]^+$; HRMS (EI) Exact mass calculated for $[C_{24}H_{30}N_1O_4]$ requires 396.2169 found 396.2170

Three component one pot asymmetric synthesis of 55

Terephthalaldehyde (8.8 mg, 0.065 mmol,), *O-tert*-butoxy aniline (21 mg, 0.13 mmol), and catalyst (S)-**21** (5.4 mg, 0.0065 mmol, 5%) were added to a flame dried biotage 2 mL microwave vial under nitrogen. 1 mL of chloroform was added (pre-dried over 4Å molecular sieves), followed by ~40 mg of powdered 4Å molecular sieves and the vial sealed with a PTFE crimp cap. After stirring at room temperature for 6 h, the reaction was cooled to -60 °C. After 30 minutes, *tert*-butyl diazoacetate (20 μL, 0.142 mmol) was added *via* syringe, and the reaction mixture was stirred at -60 °C, monitoring by ^{1}H-NMR until the reaction was deemed complete. At this point the reaction mixture was passed through a short plug of silica, eluting with diethyl ether. The solvents were removed under reduced pressure, and the residue was purified by flash chromatography (12 % diethyl ether in petroleum ether). A sample was submitted to chiral column analytical HPLC analysis [Chiralpak AD, *iso*-hexane / *iso*-propanol : 95/ 5, 1 mL / min, 4.47 min (1st peak), 6.01 min (2nd peak), 99 % *e.e.*]. The reaction product (**55**) was a colourless oil afforded in a 75 % yield.

Characterization of the chiral non-racemic product **55**

^{1}H NMR ($CDCl_3$, 400 MHz) δ 7.50 (d, 4H, J = 3.15 Hz, ArH), 7.14-6.81 (m, 8H, ArH), 3.46 (d, 2H, J = 6.77 Hz, C_2-H), 3.03 (d, 2H, J = 6.77 Hz, C_3-H), 1.37 (s, 18H, $C(CH_3)_3$), 1.27 (s, 18H, $C(CH_3)_3$); ^{13}C NMR ($CDCl_3$, 100 MHz 167.1, 167.0, 148.0, 147.9, 146.9, 146.8, 134.6, 134.6, 127.6, 127.5, 123.3, 123.2, 123.1, 123.0, 121.1, 81.2, 80.3, 80.3, 47.6, 47.5, 47.4, 47.3, 28.6, 27.8 ppm; $[\alpha]_D^{23}$ -43.7 (c 0.7 $CHCl_3$); FT-IR (thin film cm^{-1}) 2977, 1745, 1716, 1450, 1367, 1262, 1163; MS (ES) 679.4 $[M+Na]^+$; HRMS (EI) Exact mass calculated for $[C_{40}H_{53}N_2O_6]$ requires 656.3898 found 657.3895

Three component one pot asymmetric synthesis of 57

A Radley carousel tube was charged with a magnetic stirrer bar. The tube was flame dried and then cooled to room temperature under an inert gas using a Schlenk line. 2-*Tert*-butoxy-4-methoxyaniline **56** (51 mg, 0.26 mmol), 4-nitrobenzaldehyde (39.3 mg, 0.26 mmol), (S)-**21** (21 mg, 0.026 mmol, 10mol%) and 4Å molecular sieves (~100 mg) were loaded into the reaction tube. Three vacuum / nitrogen cycles were applied to the carousel tube using the Schlenk line. Anhydrous chloroform (1 mL) was injected into the carousel tube *via* the rubber seals on the tube. The reaction tube was then placed in the cooling bath at −60 °C. A flux of an inert gas was allowed from the Schlenk line into the reaction tube until the cooling had taken place. The solution was stirred for one minute. *tert*-Butyl diazoacetate (0.038 mL, 0.28 mmol, 1.1 eq^n) was added *via* syringe and the solution was stirred at −60 °C for a further 12 hours. Subsequent LC-MS analysis indicated that the intermediate imine was completely consumed. Chloroform was removed *in vacuo* from the reaction solution and the resulting residue was purified *via* flash chromatography on silica gel (elution with hexane / diethyl ether : 8 / 1). A sample of **57** was submitted to chiral column analytical HPLC analysis [Chiralpak AD, *iso*-hexane / *iso*-propanol : 95 / 5, 1 mL / min, 13.6 min (1st peak), 16.9 min (2nd peak)]. The reaction afforded **57** in 99% yield and 96% *e.e.*

Characterization of chiral non-racemic **57**.

^{1}H NMR ($CDCl_3$, 400 MHz) 8.19 (d, J = 8.83 Hz, 2H), 7.70 (d, J = 8.61 Hz, 2H), 6.83 (d, J = 8.66 Hz, 1H), 6.61 (d, J = 2.66 Hz, 1H), 6.50 (dd, J_1 = 8.67 Hz, J_1 = 2.73 Hz, 1H), 3.74 (s, 3H, OCH_3), 3.46 (d, J = 6.74 Hz, 1H, C_2-H), 3.06 (d, J = 6.80 Hz, 1H, C_3-H), 1.34 (s, 9H, OC(CH_3)$_3$), 1.21 (s, 9H, OC(CH_3)$_3$); ^{13}C NMR ($CDCl_3$, 75 MHz) δ 165.4, 154.8, 147.7, 146.3, 142.2, 137.9, 128.0, 121.9, 119.7, 108.5, 106.1, 80.8, 79.5, 54.5, 46.8, 45.9, 27.7, 26.8; $[\alpha]_D^{26}$ = −84.6 (c = 4.3, $CHCl_3$), FT-IR (thin film, cm^{-1}): 2979, 2934, 1742, 1715, 1606, 1585, 1517, 1498, 1346, 1226, 1151, 1046, 972, 912, 854, 810, 736; MS (EI)$^+$: *m/z* 443.1 [M+H]$^+$, 465.2 [M+Na]$^+$; HRMS (EI)$^+$: exact mass calculated for $[C_{24}H_{30}N_2O_6\ H]^+$ requires *m/z* 443.2177, found *m/z* 443.2176.

Synthesis of 58

Ammonium cerium(IV) nitrate (1.56 g, 2.85 mmol, 2.2 eq^{n}) was dissolved in water (7 mL) and slowly added to a 50 mL round-bottom flask containing an ice-cold solution of *cis*-**57** (573 mg, 1.2 mmol) in acetonitrile (26 mL). Soon after the addition of CAN, the solution turns in sequence: turbid brown, clear orange and again turbid brown within a minute. The reaction mixture was stirred at room temperature and monitored by TLC until complete consumption of the starting material **57**. The pH of the solution was then adjusted to neutral by addition of a few drops of 5% aqueous $NaHCO_3$ solution. Solid sodium sulfite was added in small portions until a brown slurry formed. After extraction with ethyl acetate (3 x 10 mL), the combined organic phases were dried over anhydrous magnesium sulfate and concentrated under reduced pressure. Purification *via* flash chromatography on silica gel (eluent diethyl ether / hexane : 1 / 1 →2 / 1→diethyl ether) afforded 200 mg of a white solid in 67% yield. Subsequent physicochemical analysis of the purified product confirmed formation of **58**.

Characterization of chiral non-racemic **58**

M.p. = 133.5 – 135.6 °C; ^{1}H NMR (300 MHz, $CDCl_3$): 8.18 (d, 2H, J = 8.88 Hz), 7.55 (d, 2H, J = 8.50 Hz), 3.50(br s, 1H, C_2-*H*), 3.06 (d, 1H, J = 6.53 Hz, C_3-*H*), 1.9 (br s, 1H, N*H*), 1.19 (s, 9H, $OC(CH_3)_3$); ^{13}C NMR (75 MHz, $CDCl_3$): δ 167.5, 147.4, 142.8, 128.8, 123.2, 82.2, 38.6, 38.1, 27.7 ppm; $[\alpha]_D^{26}$ = −8.0 (c = 1.0, $CHCl_3$), FT-IR (thin film, cm^{-1}): 3208, 2976, 2933, 1725, 1600, 1515, 1369, 1347, 1218, 1154, 908, 883, 859, 854, 733; MS (EI)$^+$: *m/z* 663.2 (100%), 465 (20%). HRMS (EI)$^+$: exact mass calculated for $[C_{13}H_{16}N_2O_4H]^+$ requires *m/z* 265.1188, found *m/z* 265.1190.

Synthesis of 59

A 10 mL two-necked round-bottom flask was equipped with a condenser and a magnetic stirrer bar. The flask was loaded with a solution of *cis*-**59** (160 mg, 0.6 mmol) in 2.5 mL of 1,2-dichloroethane. Dichloroacetic acid (0.5 mL, 6 mmol, 10 eq^{n}) was added to the solution and the mixture was heated at reflux for one hour. The excess dichloroacetic acid was removed by evaporation. The resulting residue was dissolved in dichloromethane and treated with saturated aqueous solution of sodium carbonate. The aqueous layer was extracted with dichloromethane and the combined organic extracts were dried over anhydrous magnesium sulfate. Purification *via* flash chromatography on silica gel (hexane / diethyl ether : 1 / 2) afforded 180 mg of **59** in 76% yield. Subsequent physicochemical analysis of the purified product confirmed formation of **59**.

Characterization of chiral non-racemic **59**

^{1}H NMR (300 MHz, $CDCl_3$): 8.20 (d, 2H, J = 8.68 Hz), 7.58 (d, 2H, J = 8.83 Hz), 7.17 (d, 1H, J = 9.35 Hz), 5.77 (s, 1H, C*H*Cl_2), 5.49 (s, 1H, C_3-*H*), 4.77 (dd, 1H, J_1 = 9.13 Hz, J_2 = 2.61 Hz, C_2-*H*), 2.98 (s, 1H, N*H*), 1.51 (s, 9H, OC(CH_3)$_3$). ^{13}C NMR (75 MHz, $CDCl_3$): δ 167.8, 164.1, 147.8, 146.4, 126.9, 123.7, 84.1, 72.7, 65.9, 58.1, 27.8; $[\alpha]_D^{26}$ = + 13.3 (c = 1.0, $CHCl_3$), IR (thin film, cm^{-1}): 3406, 2958, 2926, 2854, 1732 (CO), 1683, 1608, 1520, 1348, 1259, 1157, 1074, 811, 735; MS (EI)$^+$: *m/z* 441 (20%), 365 (5%), 337 (20%), 268 (100%), 264 (50%), 247 (35%); HRMS (EI)$^+$: exact mass calculated for $[C_{15}H_{18}Cl_2N_2O_6H]^+$ requires *m/z* 421.0569, found *m/z* 421.0570.

Synthesis of compound (+)-60

A 2 mL vial was flame dried and charged with a magnetic stirrer bar. The vial was cooled to room temperature under nitrogen and **59** (29 mg, 0.07 mmol) was loaded into the vial and three vacuum / nitrogen cycles applied before the solid was dissolved in 0.2 mL of methanol and cooled to 0 °C. Sodium borohydride (14 mg, 0.4 mmol, 5 eq^n) was added to the solution all at once and the reaction mixture was stirred for 30 minutes. The product was quenched with water (2.5 mL) and extracted with ethyl acetate three times. The combined organic phases were dried over anhydrous magnesium sulfate. Purification *via* flash chromatography on silica gel (hexane / ethyl acetate : 3 / 7) afforded 19 mg of a white solid in 79% yield. Subsequent physicochemical analysis of the purified product confirmed formation of the title compound. The physico-chemical characteristics were essentially identical to those reported by Dixon *et al.*[1]

Characterization of chiral non-racemic (+)-**60**

^{1}H NMR (300 MHz, DMSO): δ 8.32 (d, 1H, J = 9.0 Hz), 8.15 (m, 2H), 7.59 (m, 2H), 6.47 (s, 1H), 6.03 (m, 1H), 5.05 (m, 1H), 4.98 (m, 1H), 3.93 (m, 1H), 3.57 (m, 1H), 3.34 (m, 1H). ^{13}C NMR (75 MHz, DMSO): δ163.4, 151.3, 146.4, 127.3, 122.9, 69.0, 66.5, 60.3, 56.9; $[\alpha]_D^{26}$ = + 23.9 (c = 1.0, EtOAc); FT-IR (thin film, cm^{-1}): 3298, 2924, 1682, 1514, 1348, 1070, 812.

[1] A. Franchino, P. Jakubec, D. J. Dixon, *Organic & Biomolecular Chemistry*, **2016**, *14*, 93 - 96

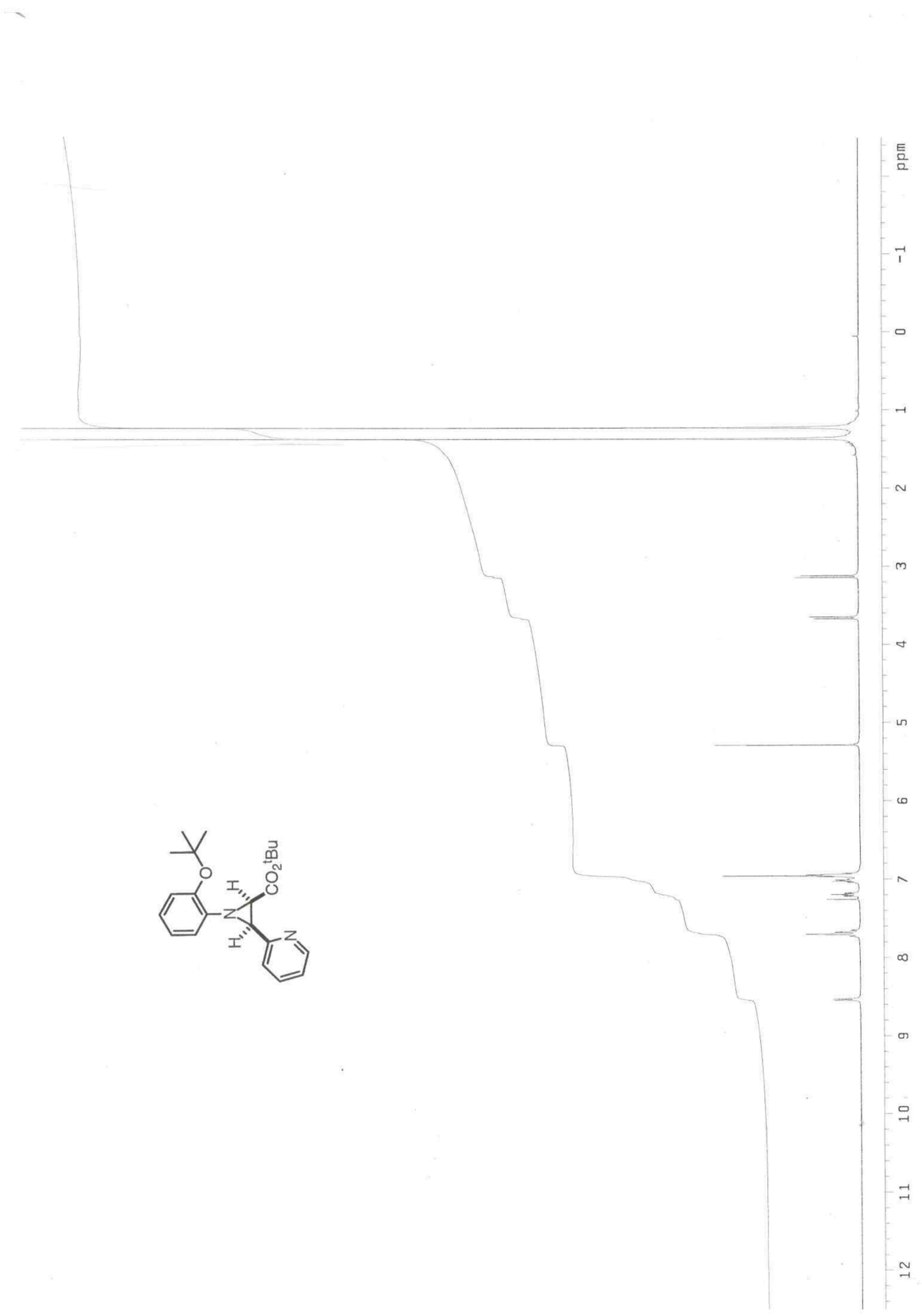

^{1}H-NMR of **27**

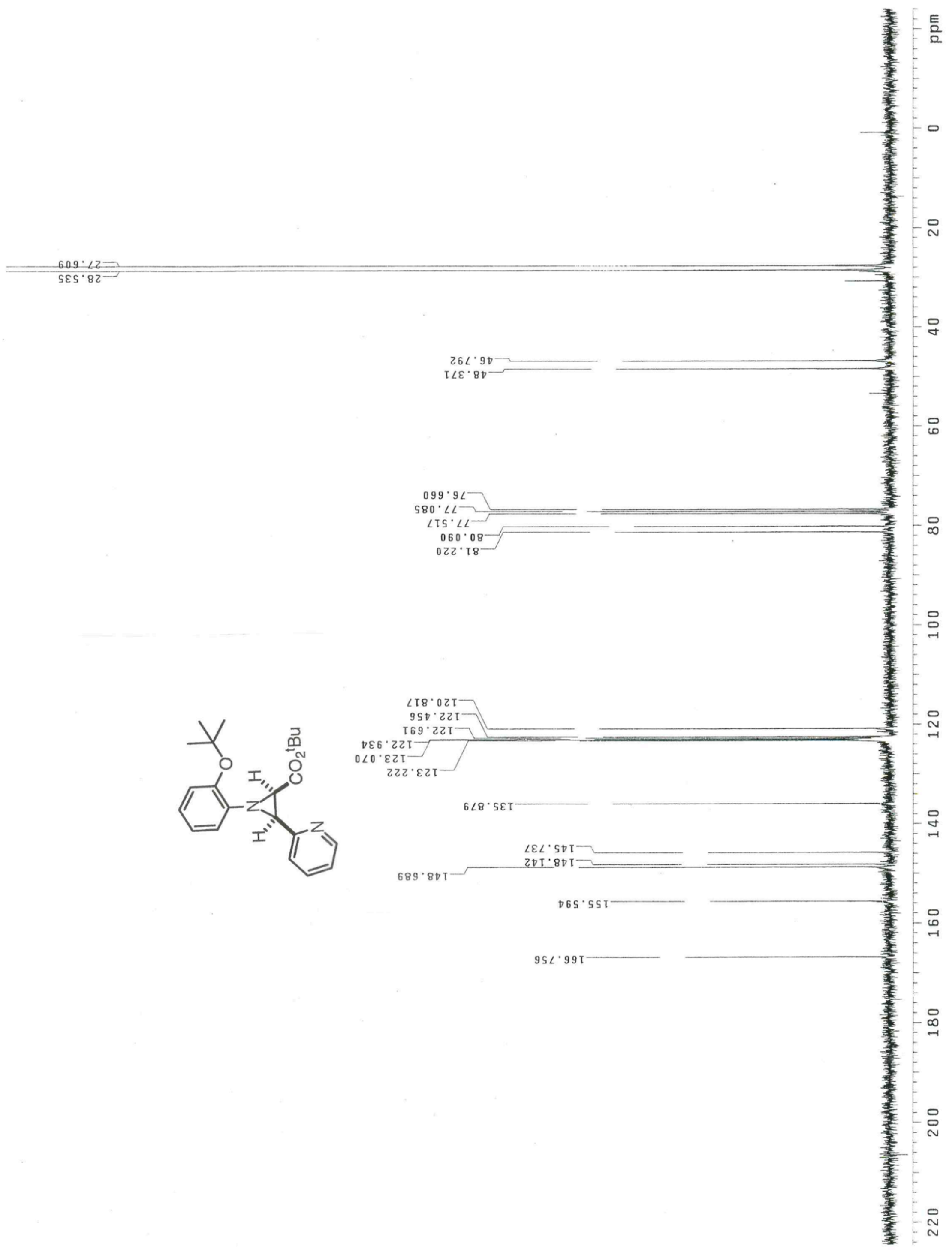

^{13}C-NMR of **27**

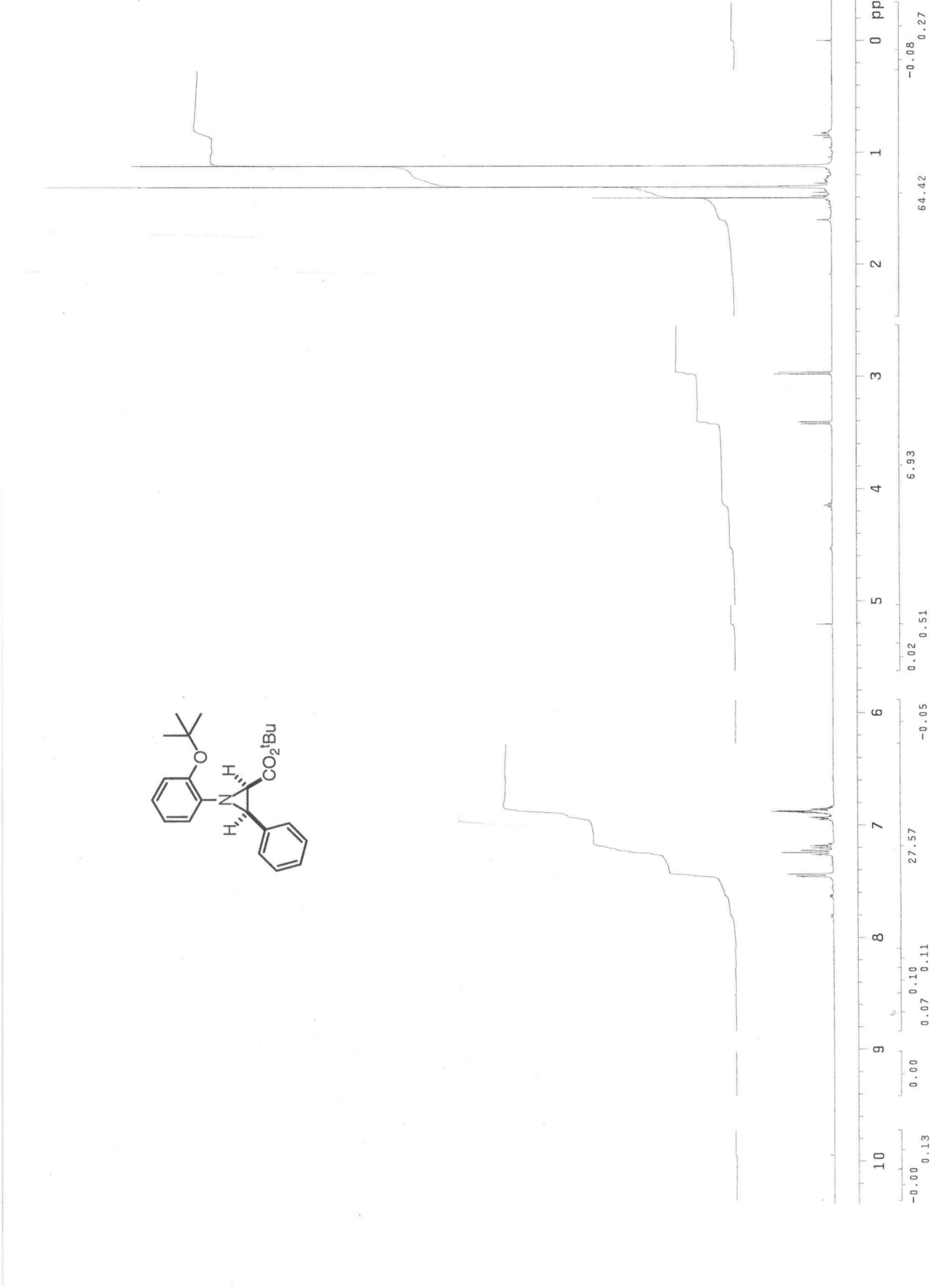

^{1}H-NMR of **38**

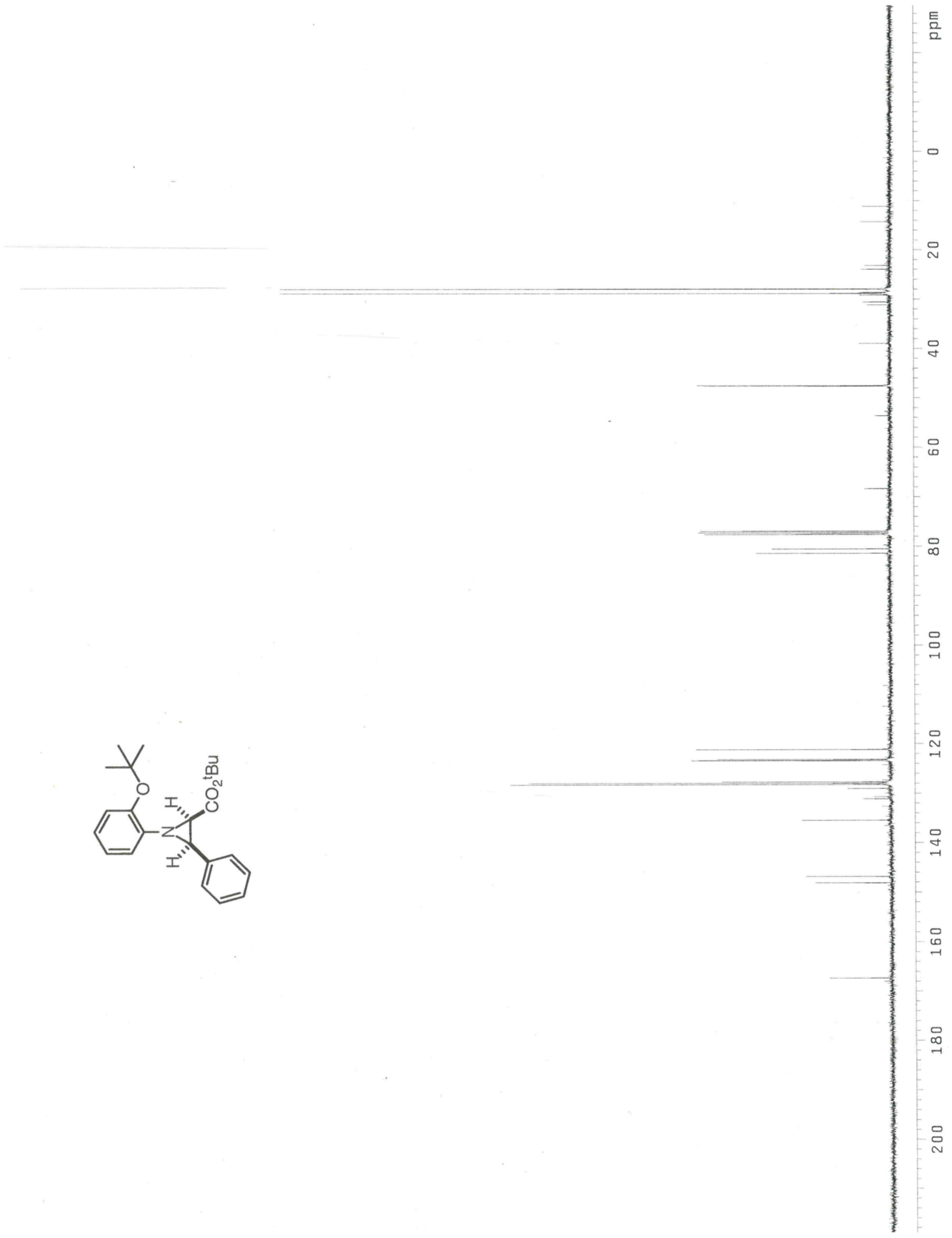

^{13}C-NMR of **38**

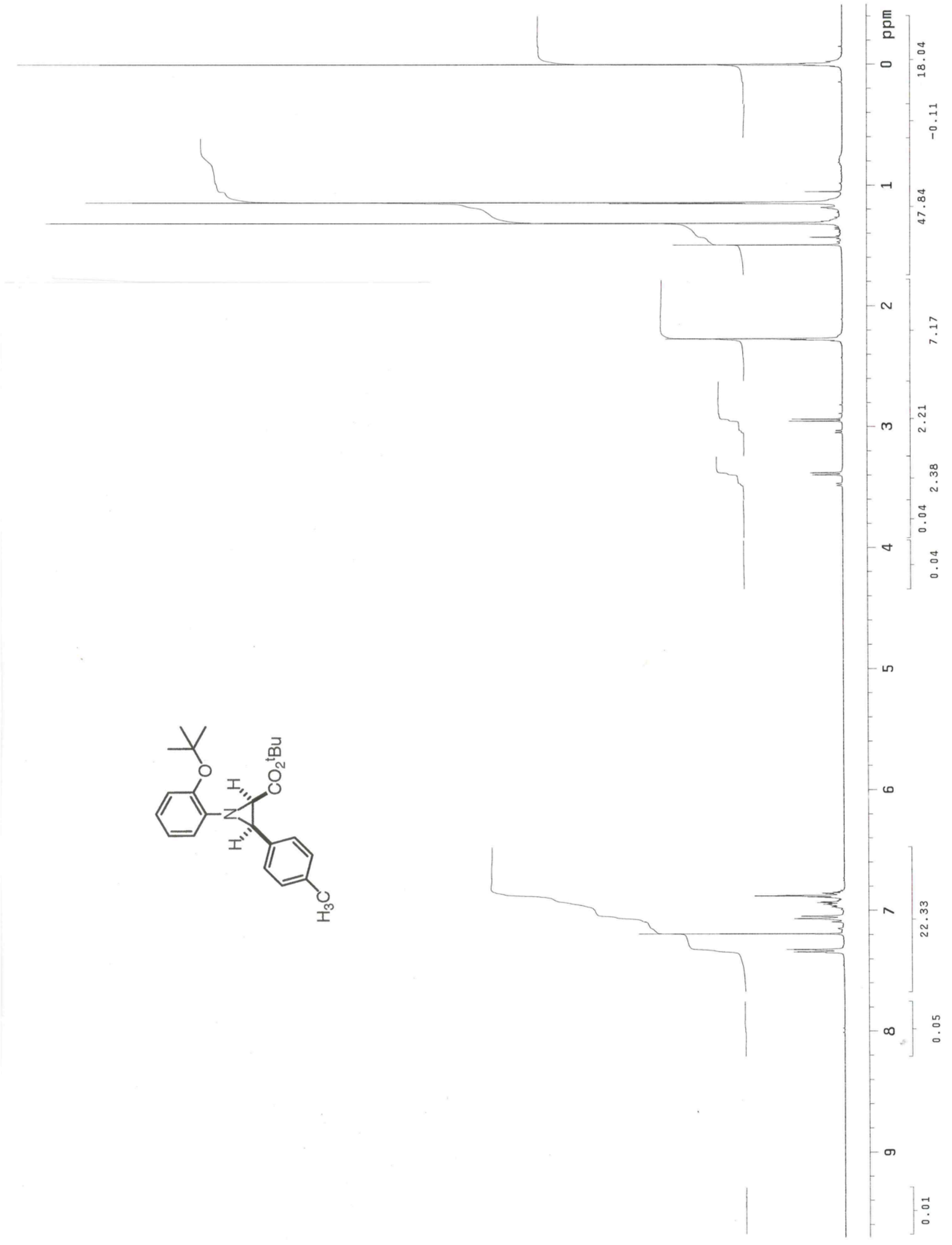
ppm
0
1
2
3
4
5
6
7
8
9
18.04
-0.11
47.84
7.17
2.21
2.38
0.04
0.04
22.33
0.05
0.01
CO2tBu
H3C
N
H
H

^{1}H-NMR of **39**

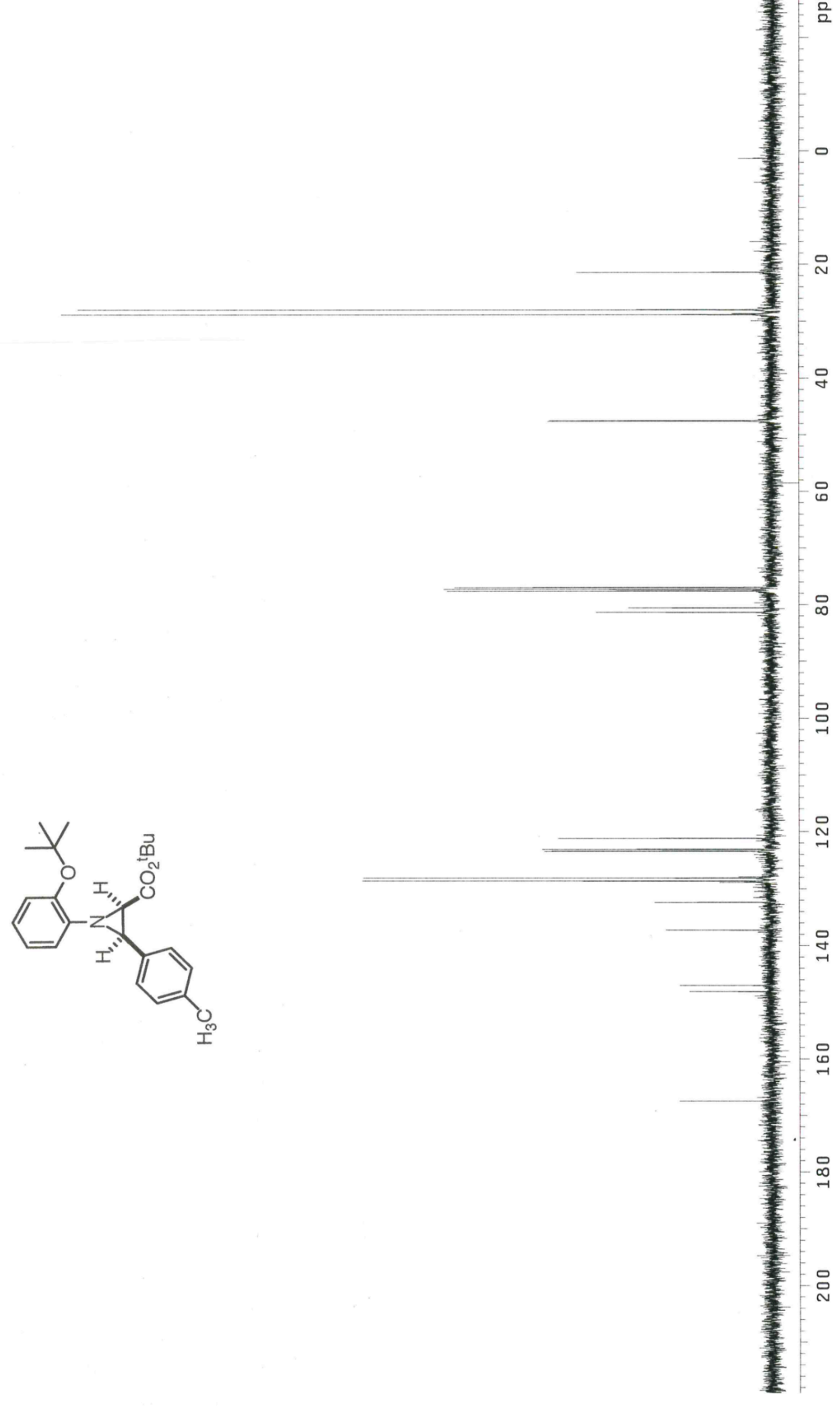

^{13}C-NMR of **39**

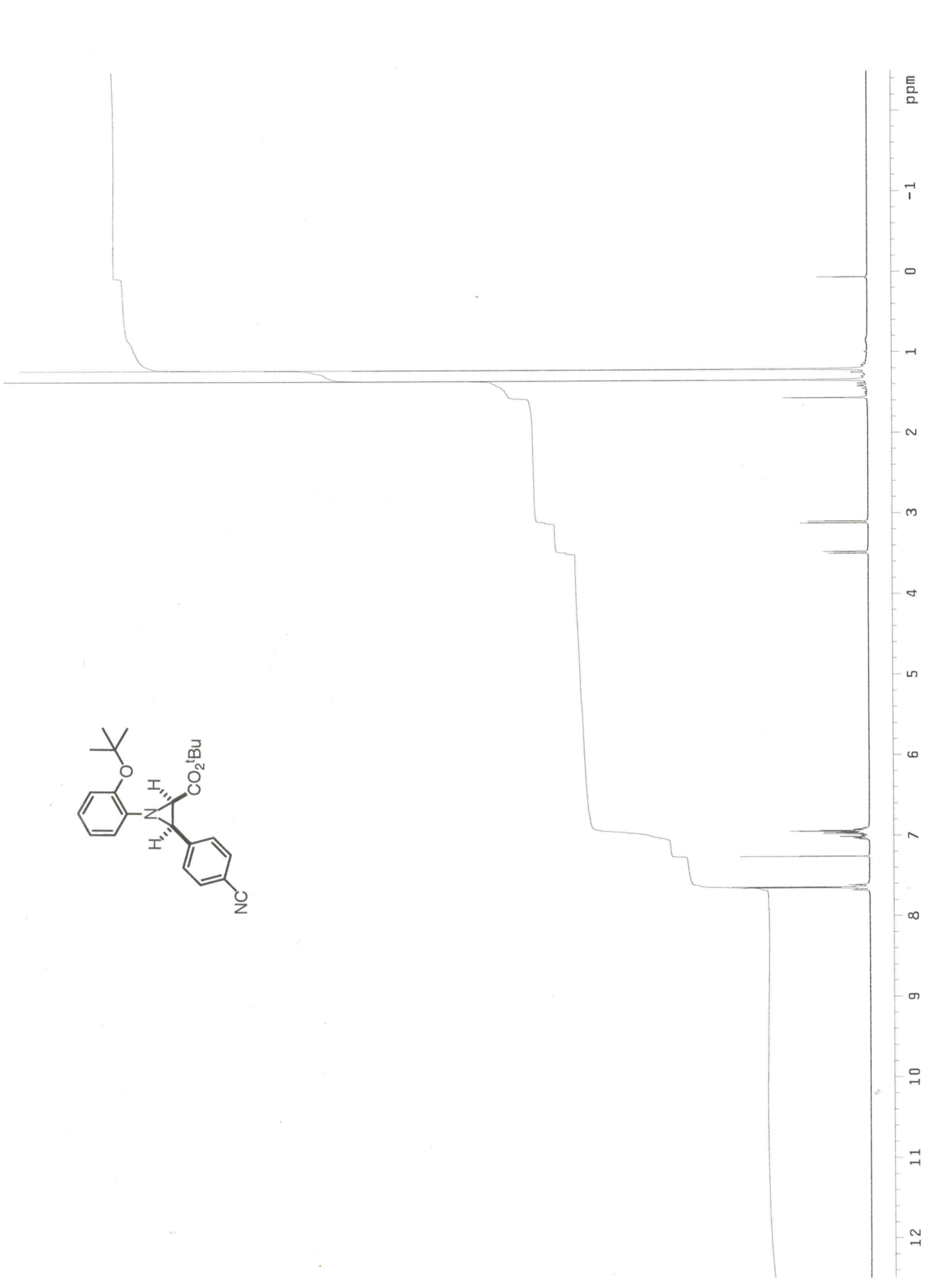

^{1}H-NMR of **40**

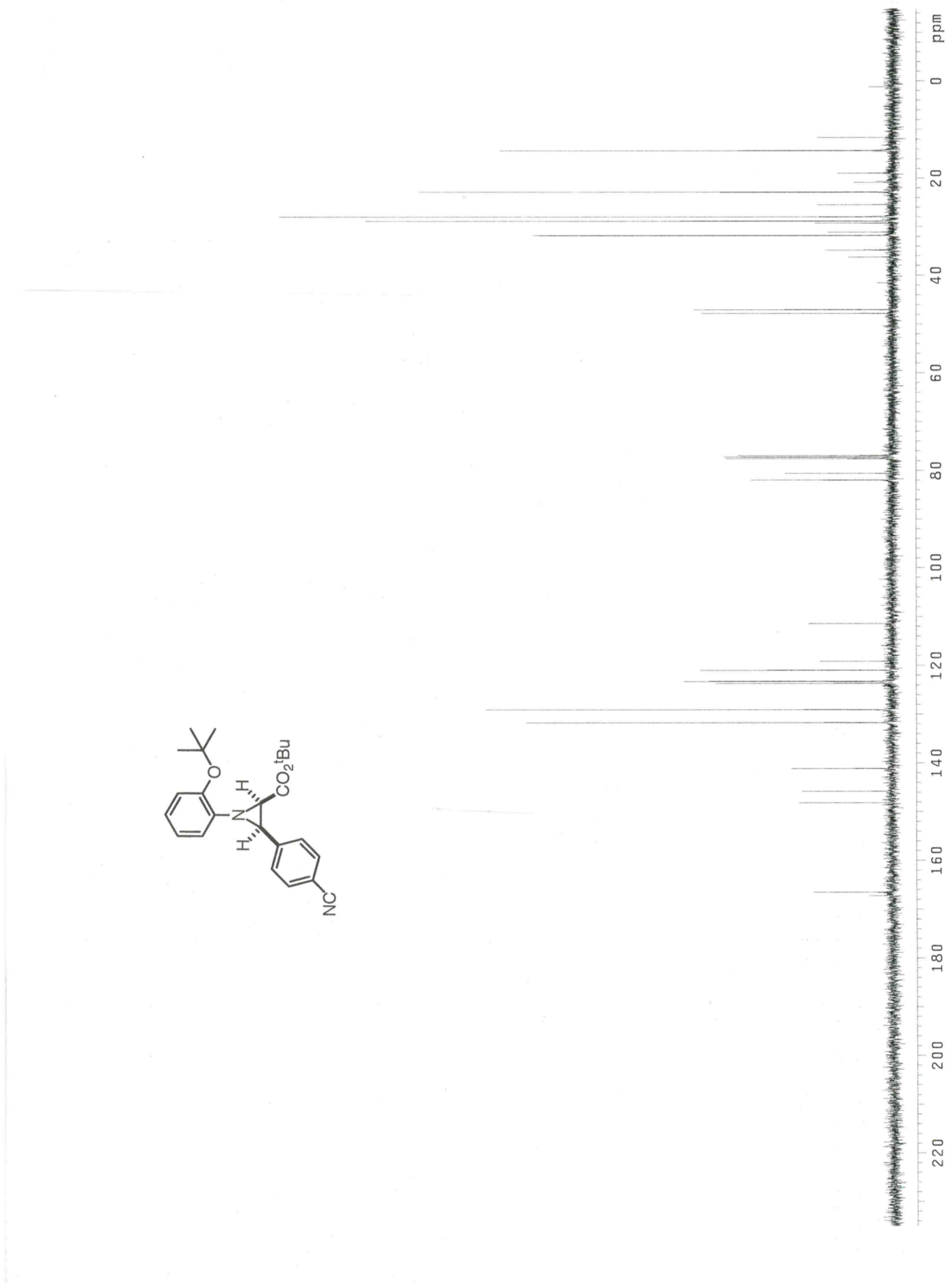

^{13}C-NMR of **40**

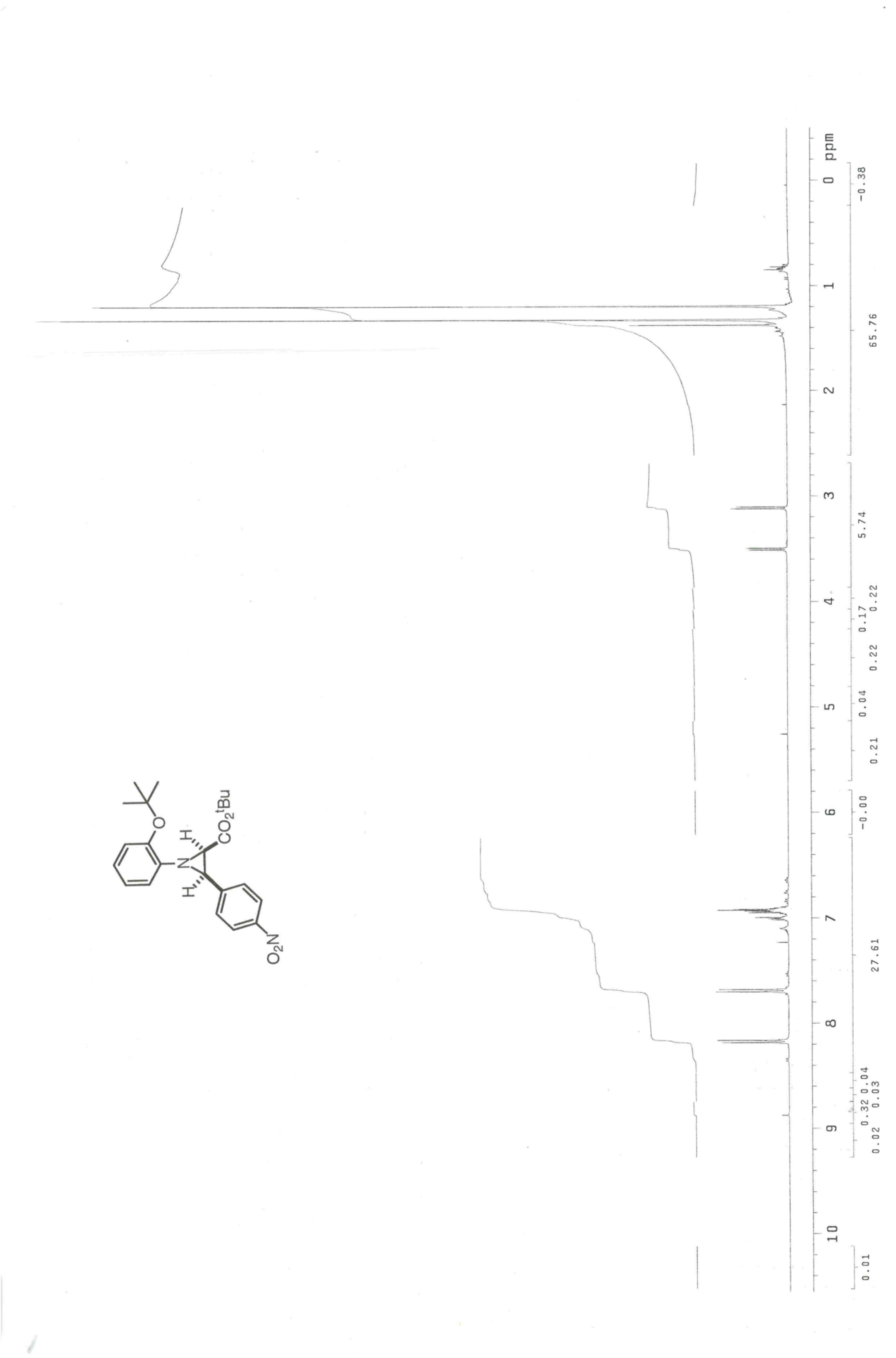
ppm
0
1
2
3
4
5
6
7
8
9
10
-0.38
65.76
5.74
0.17
0.22
0.22
0.04
0.21
-0.00
27.61
0.32
0.04
0.02
0.03
0.01
CO2tBu
O2N
N
H
H
O

^{1}H-NMR of **41**

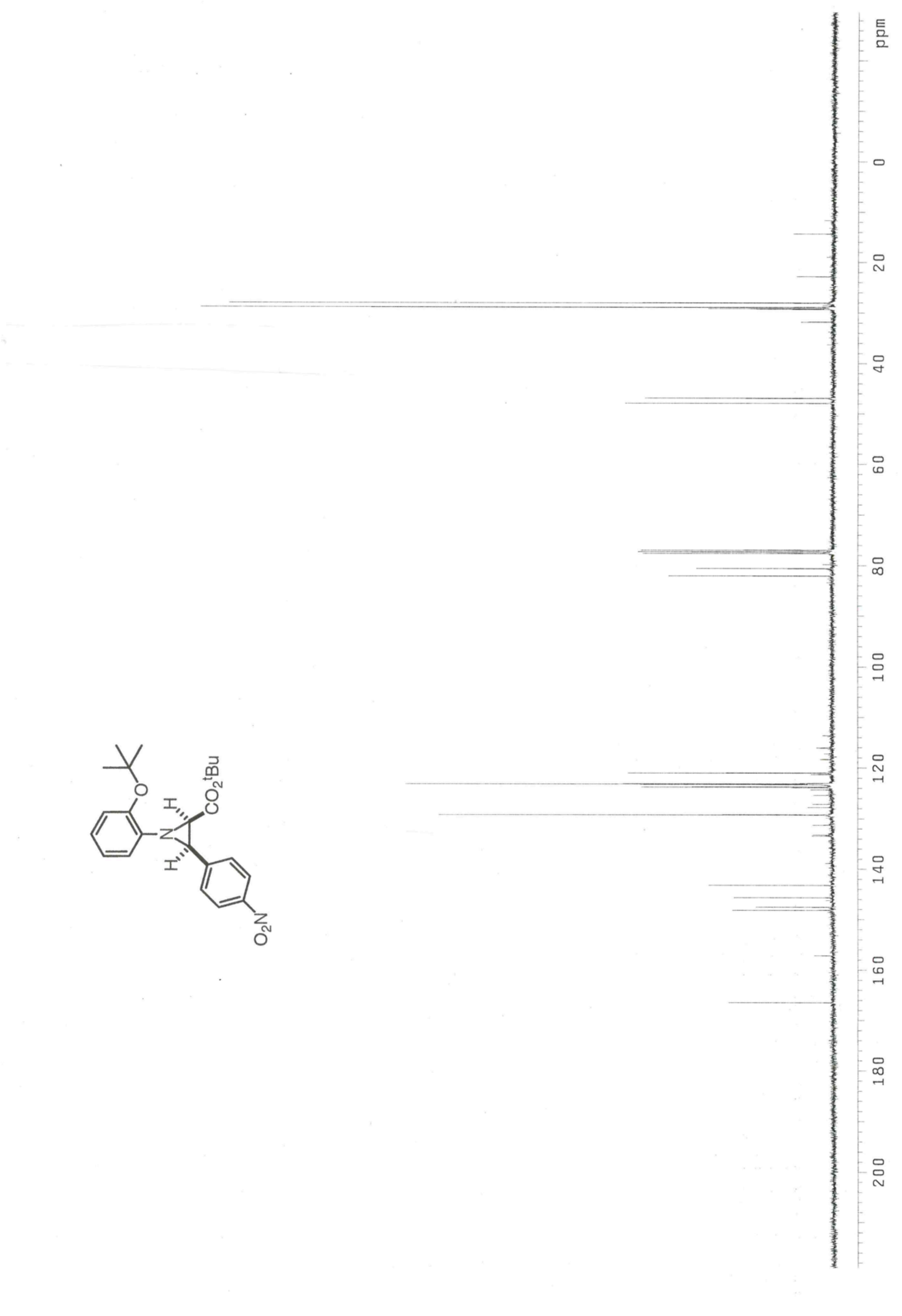

^{13}C-NMR of **41**

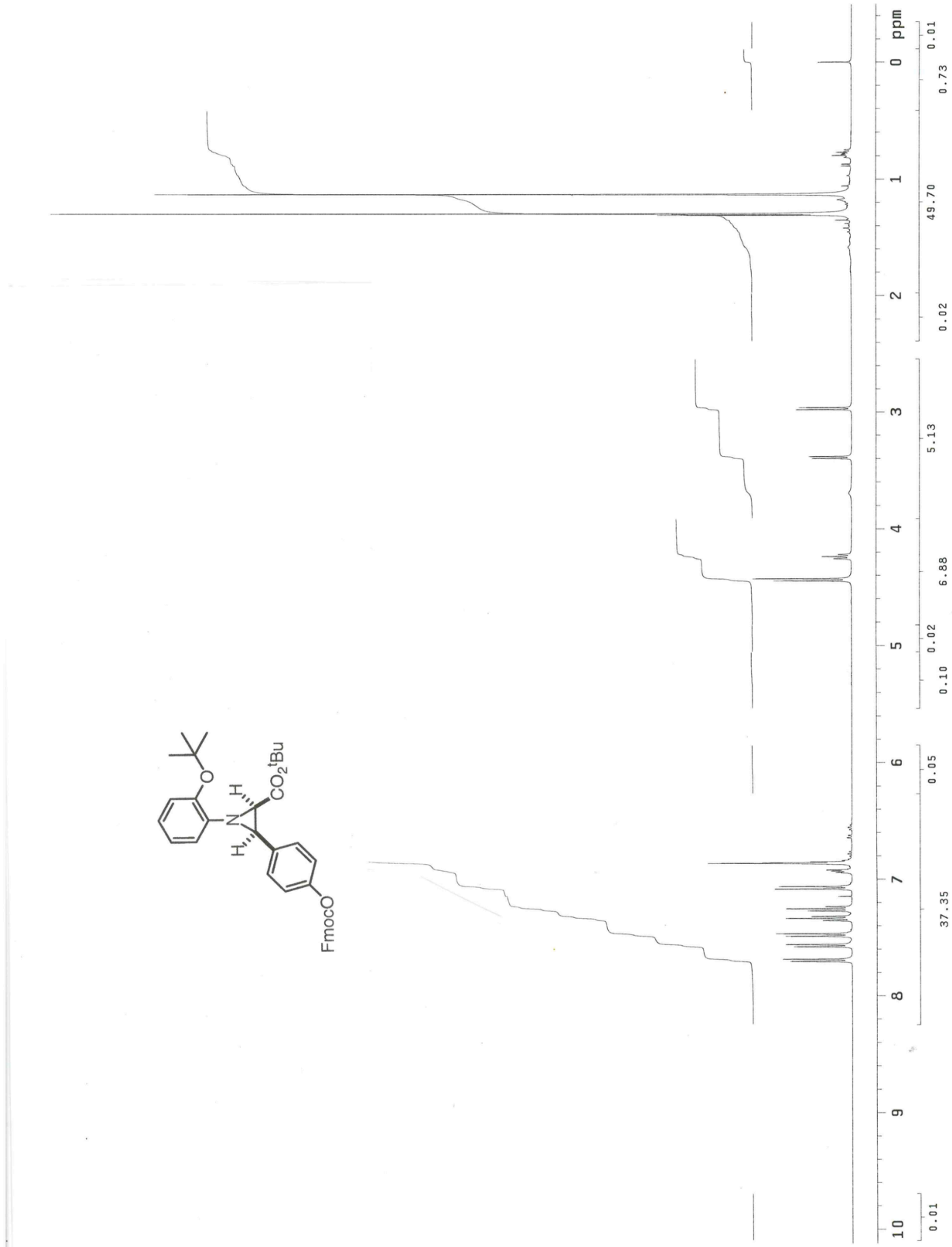
FmocO
CO2tBu
ppm
10
9
8
7
6
5
4
3
2
1
0
0.01
37.35
0.05
0.10
0.02
6.88
5.13
0.02
49.70
0.73
0.01

^{1}H-NMR of **42**

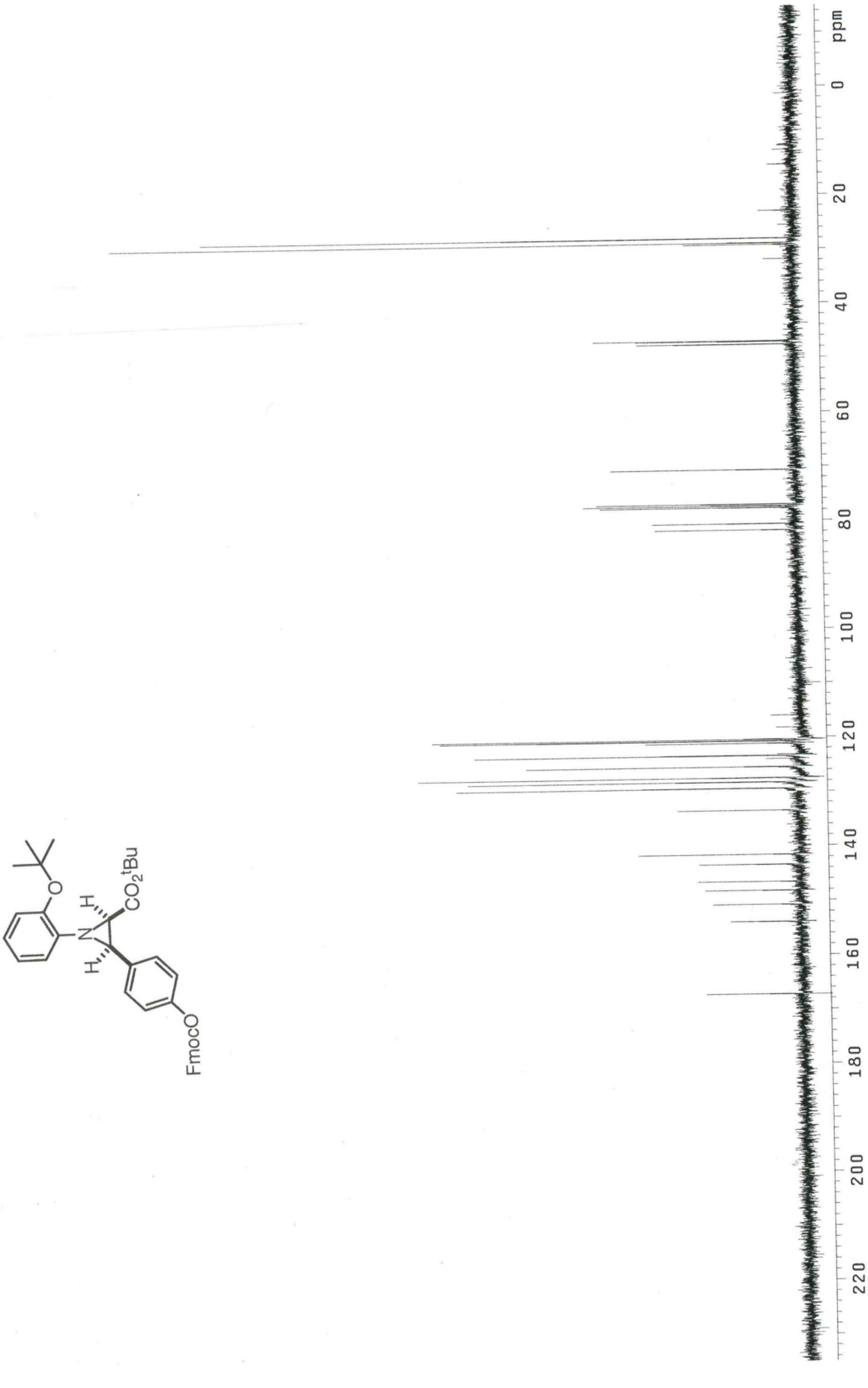

^{13}C-NMR of **42**

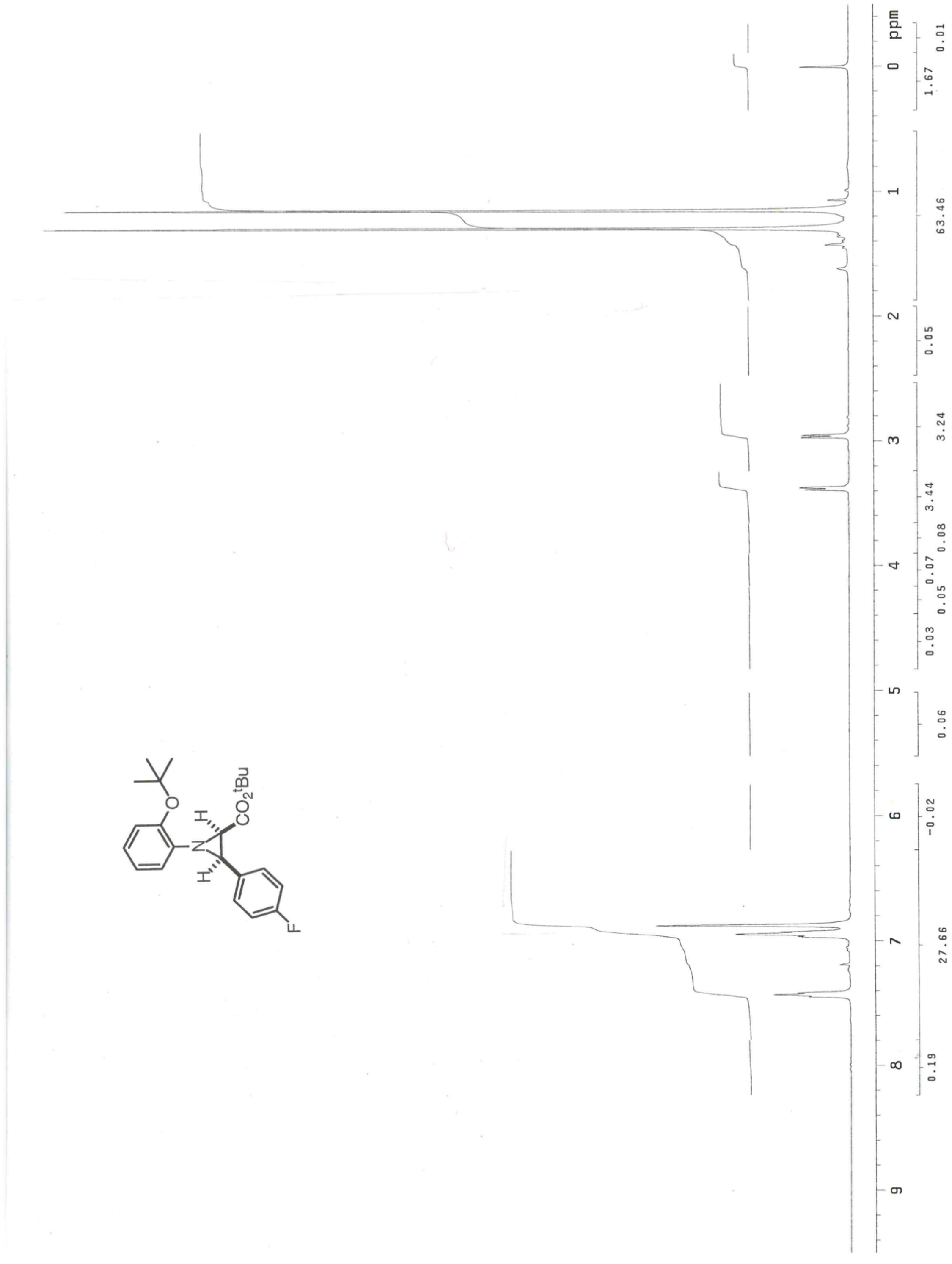
ppm
0
1
2
3
4
5
6
7
8
9
0.01
1.67
63.46
0.05
3.24
3.44
0.08
0.07
0.05
0.03
0.06
-0.02
27.66
0.19
CO2tBu
F

^{1}H-NMR of **43**

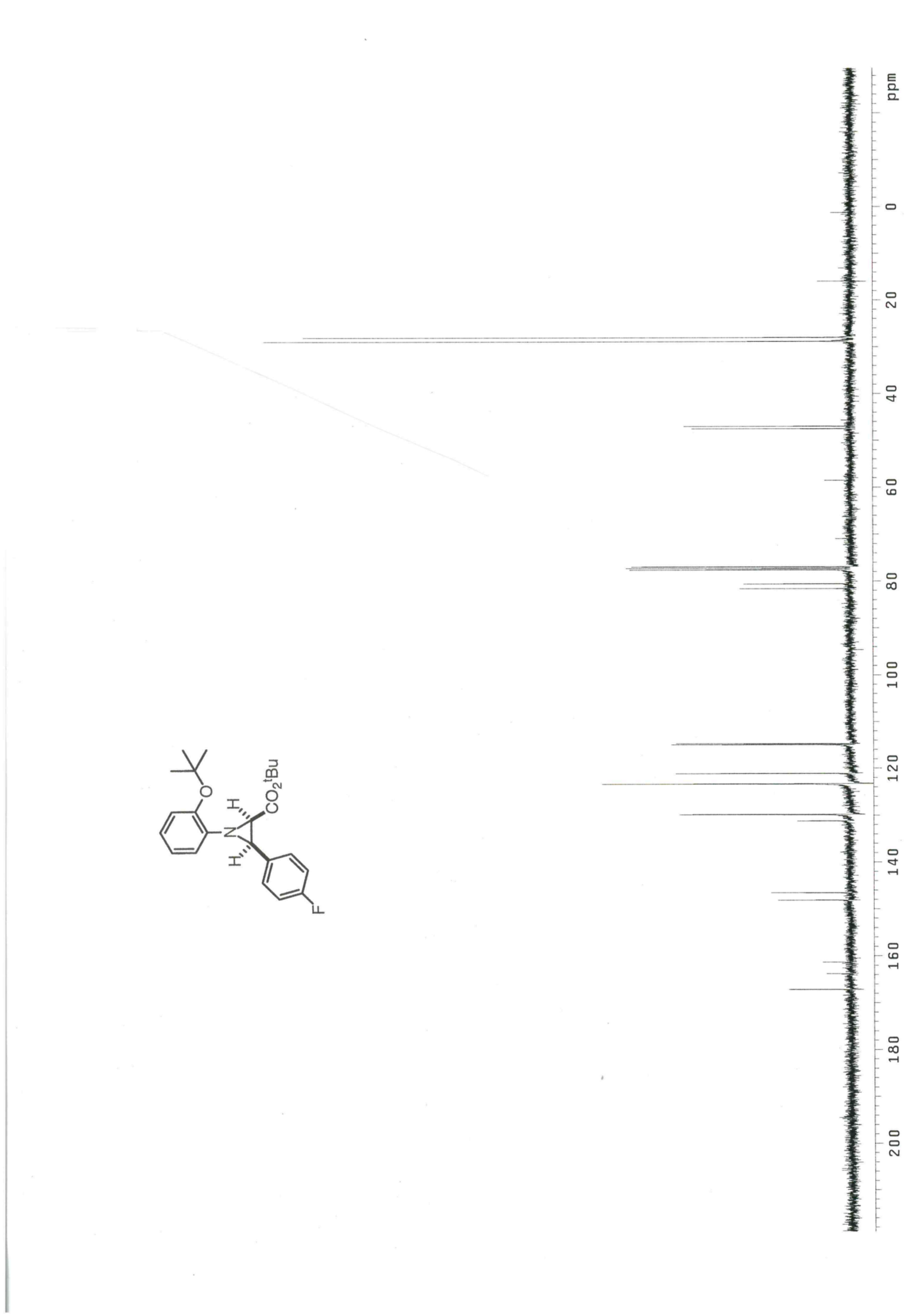

^{13}C-NMR of **43**

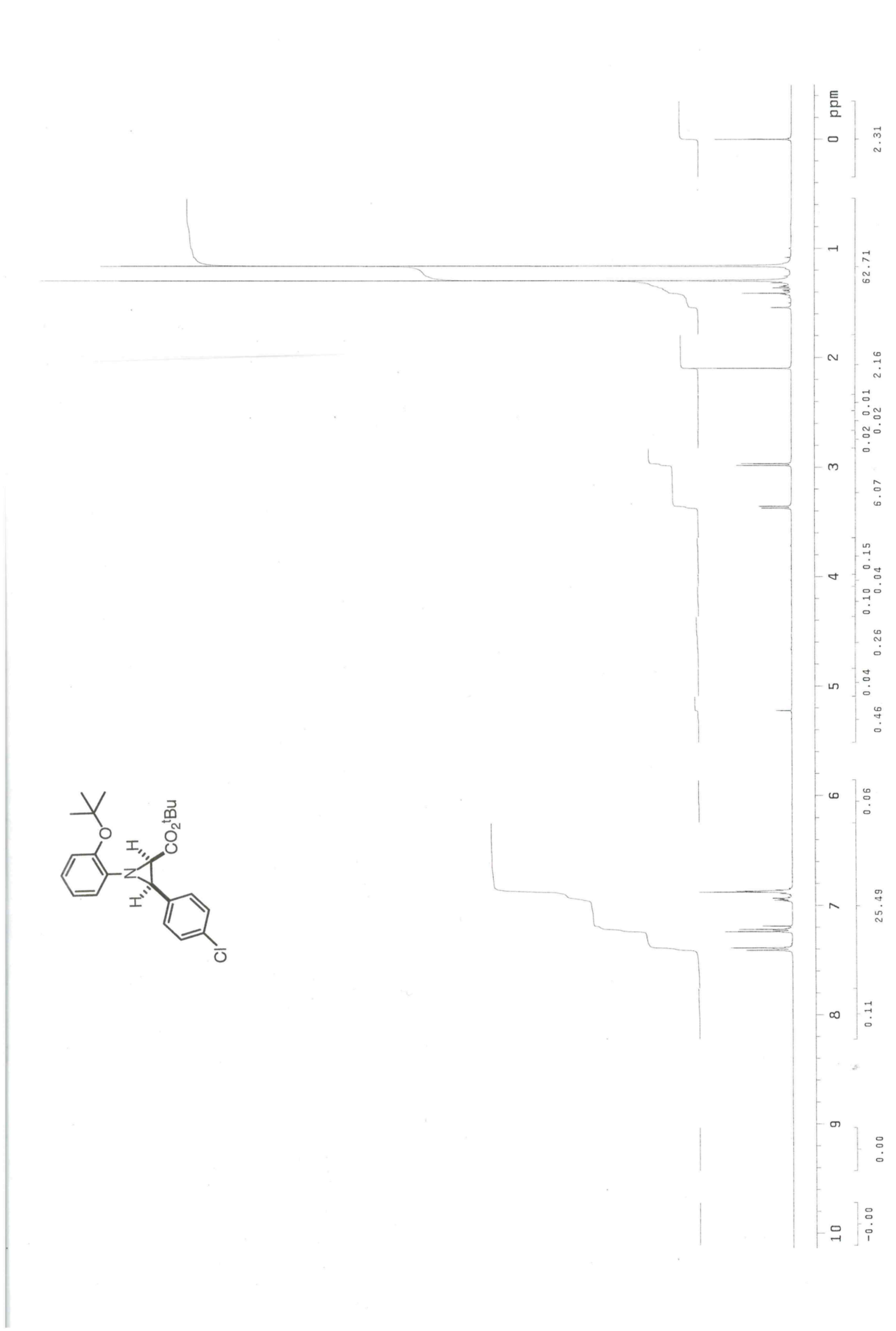

^{1}H-NMR of **44**

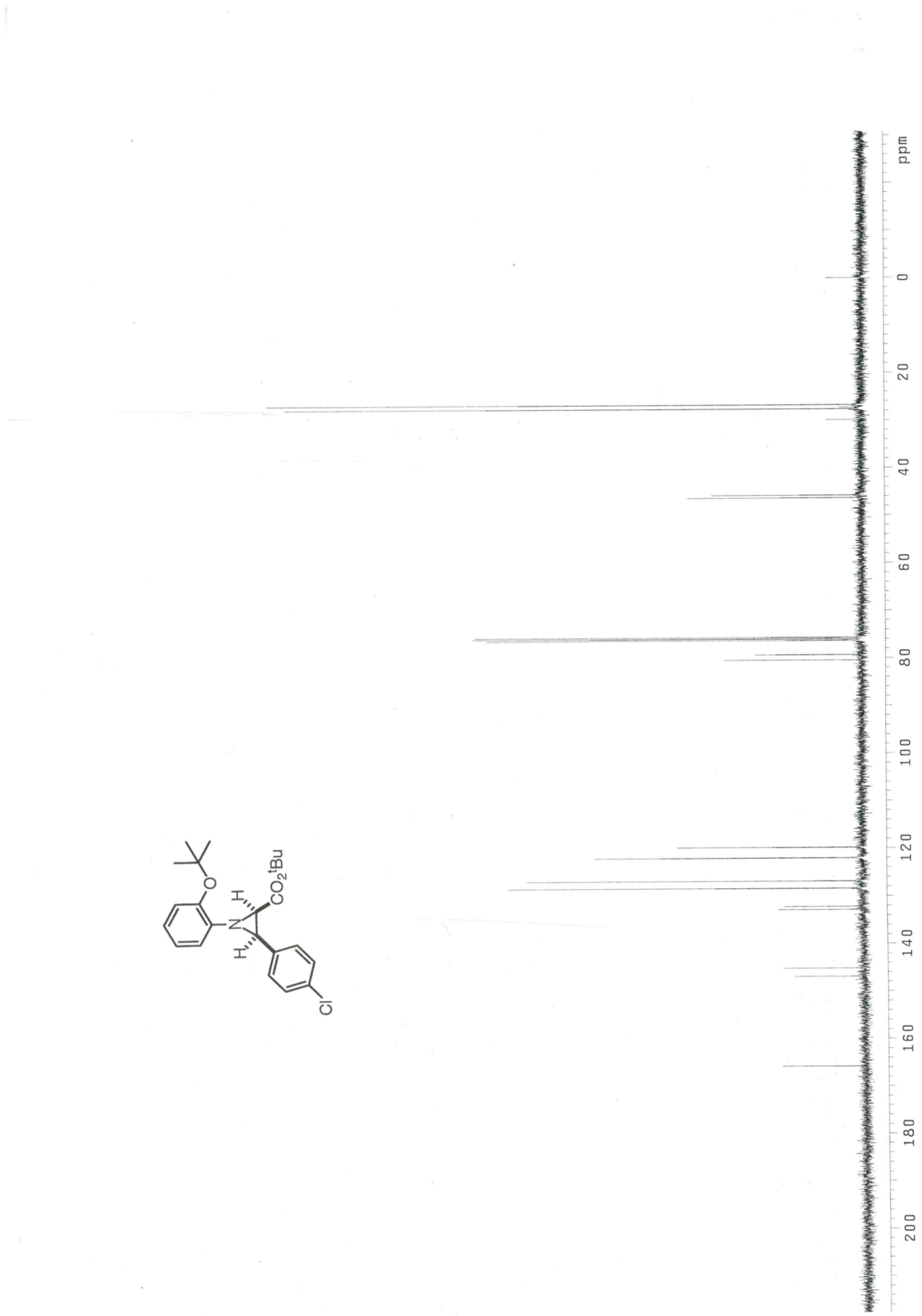

^{13}C-NMR of **44**

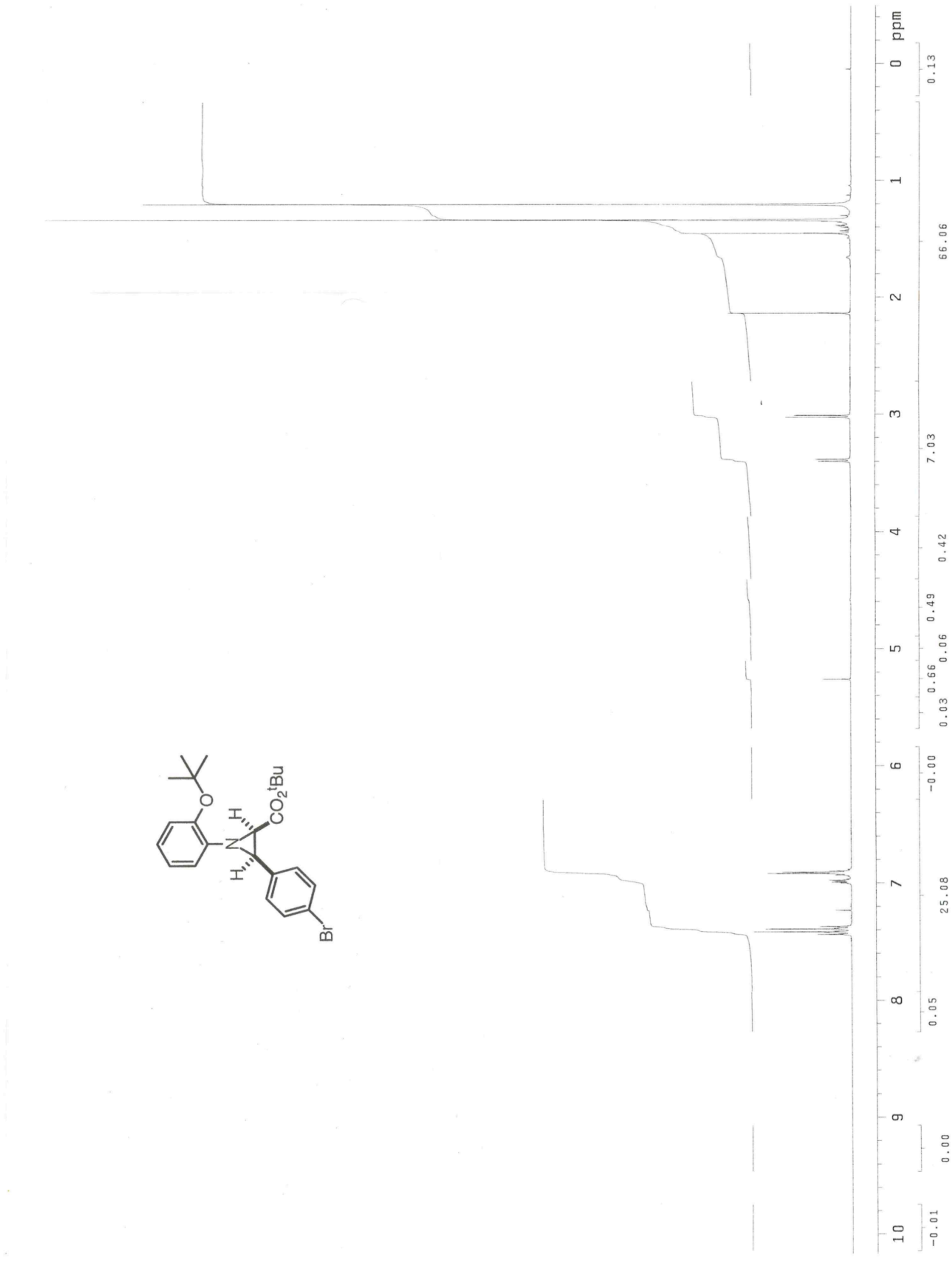

^{1}H-NMR of **45**

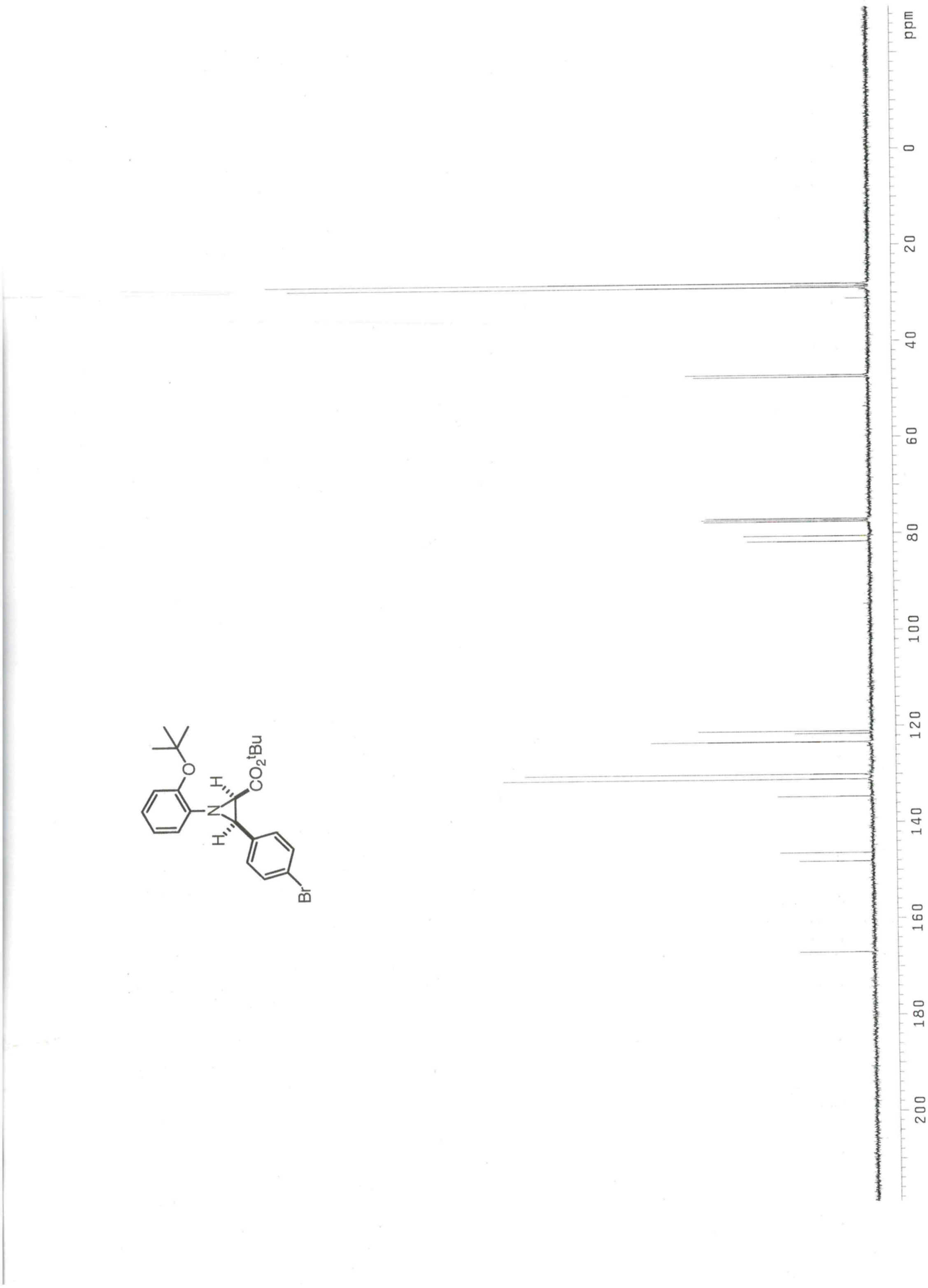

^{13}C-NMR of **45**

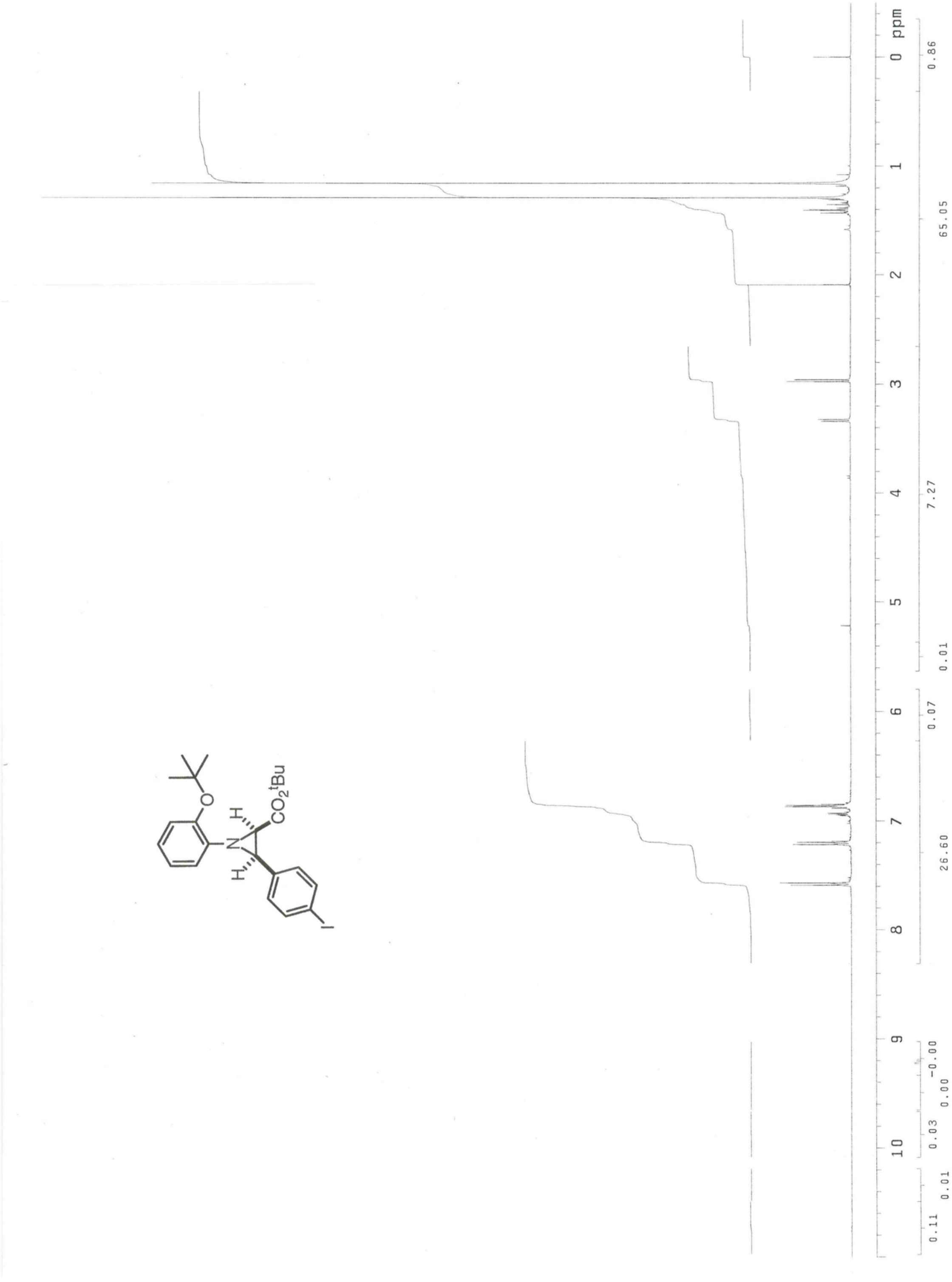

^{1}H-NMR of **46**

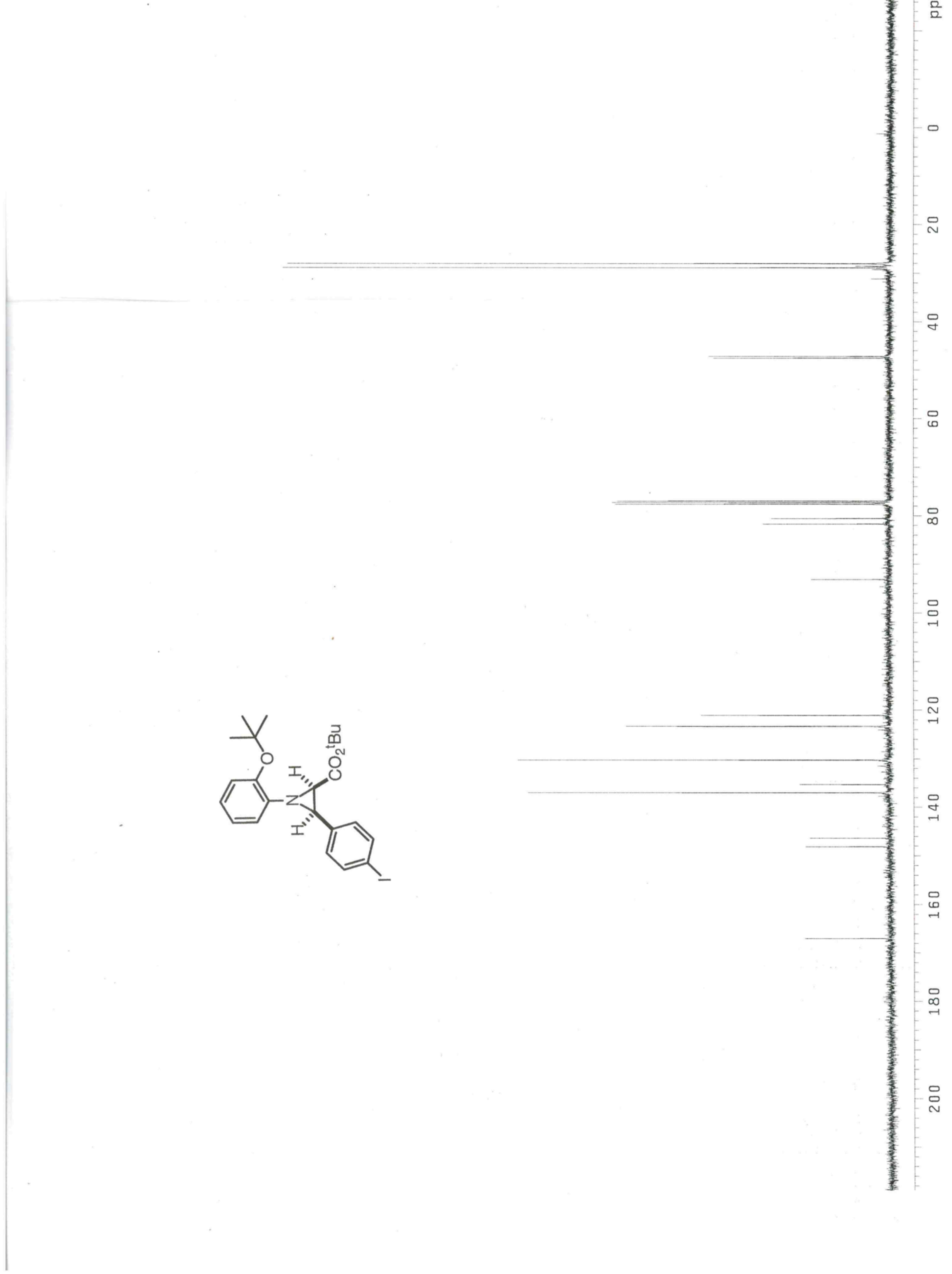

^{13}C-NMR of **46**

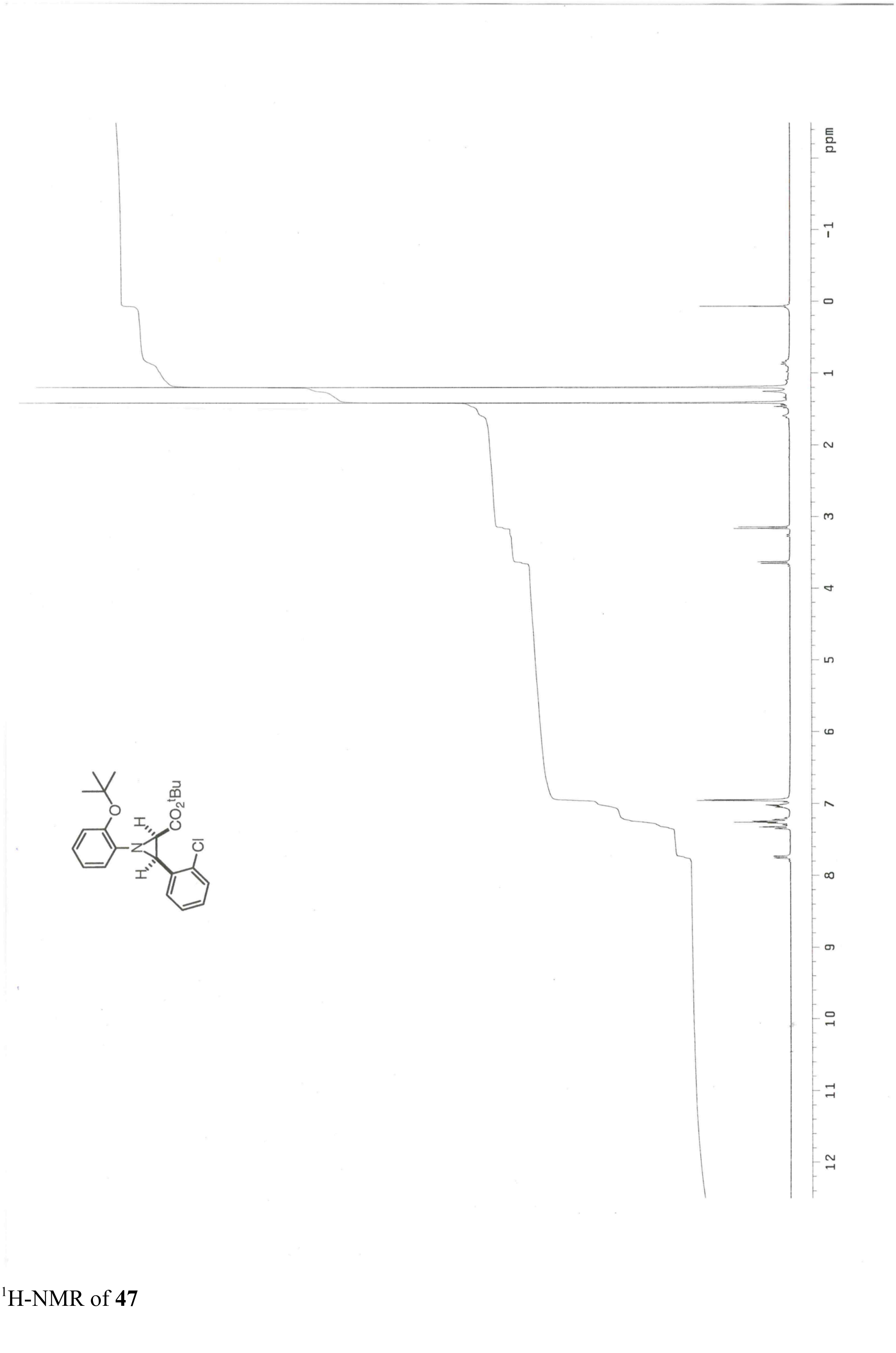

^{1}H-NMR of **47**

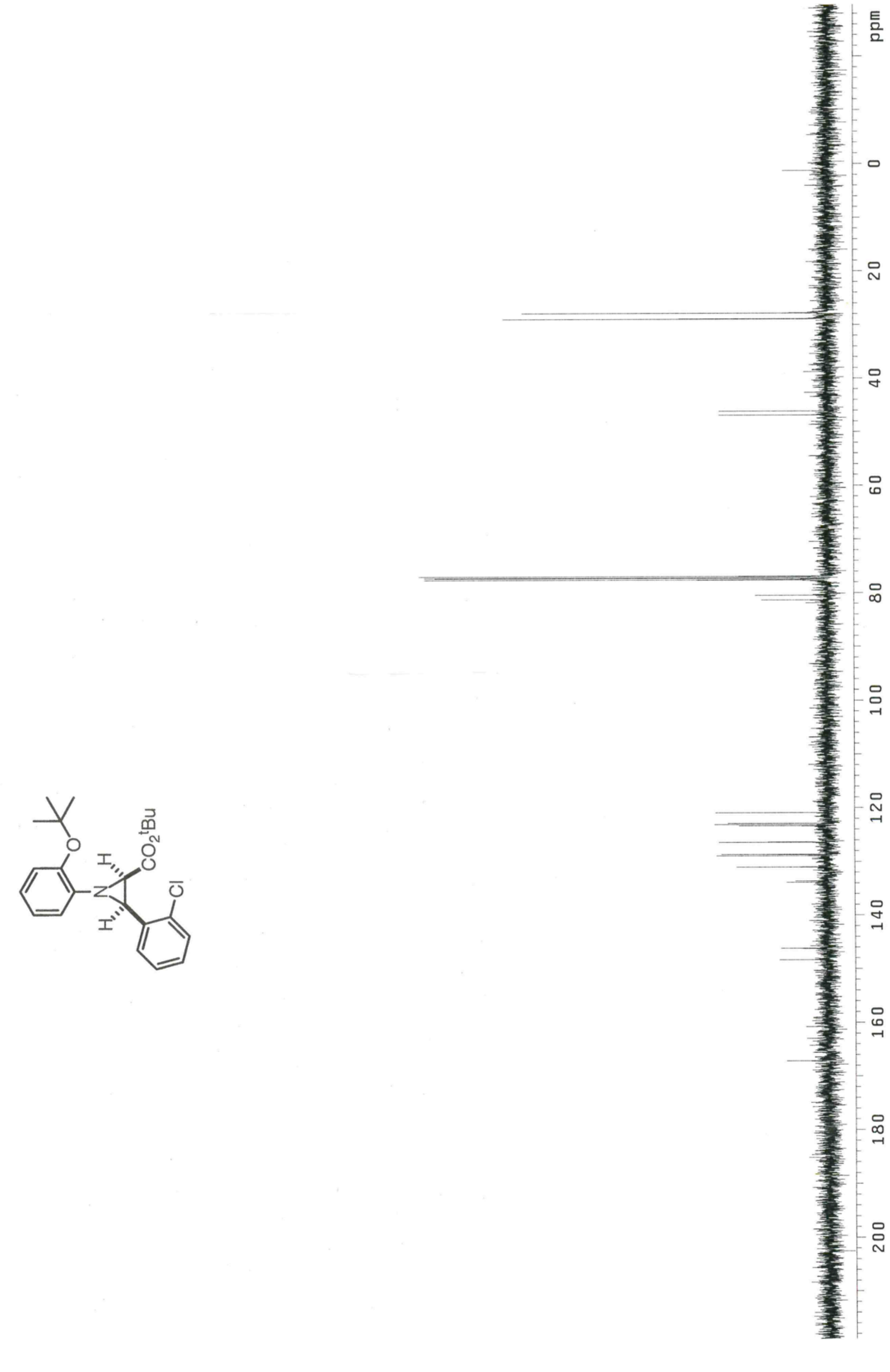

^{13}C-NMR of **47**

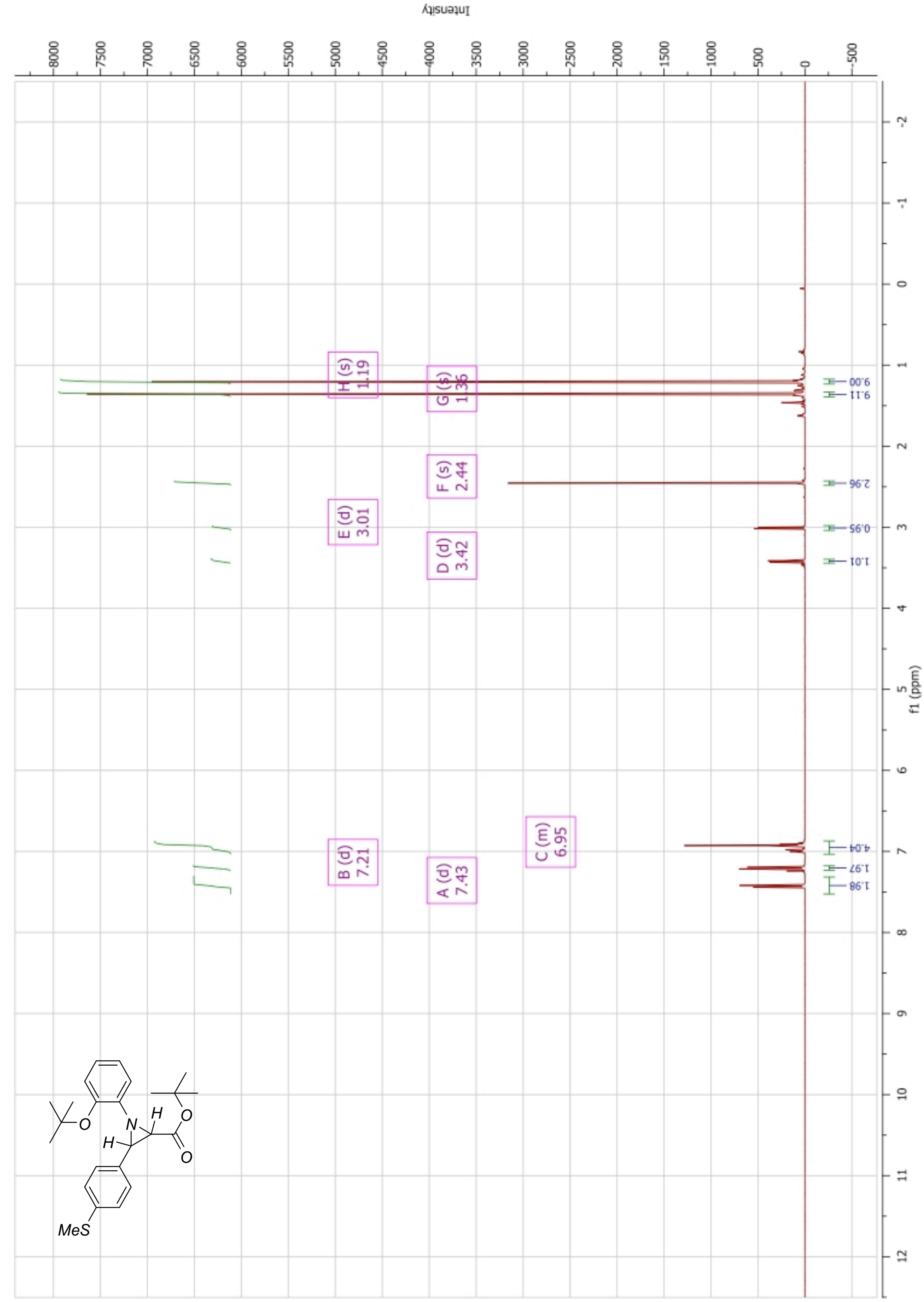
Intensity
f1 (ppm)
H (s)
1.19
G (s)
1.36
F (s)
2.44
E (d)
3.01
D (d)
3.42
C (m)
6.95
B (d)
7.21
A (d)
7.43
9.00
9.11
2.96
0.95
1.01
4.04
1.97
1.98
MeS

^{1}H-NMR of **49**

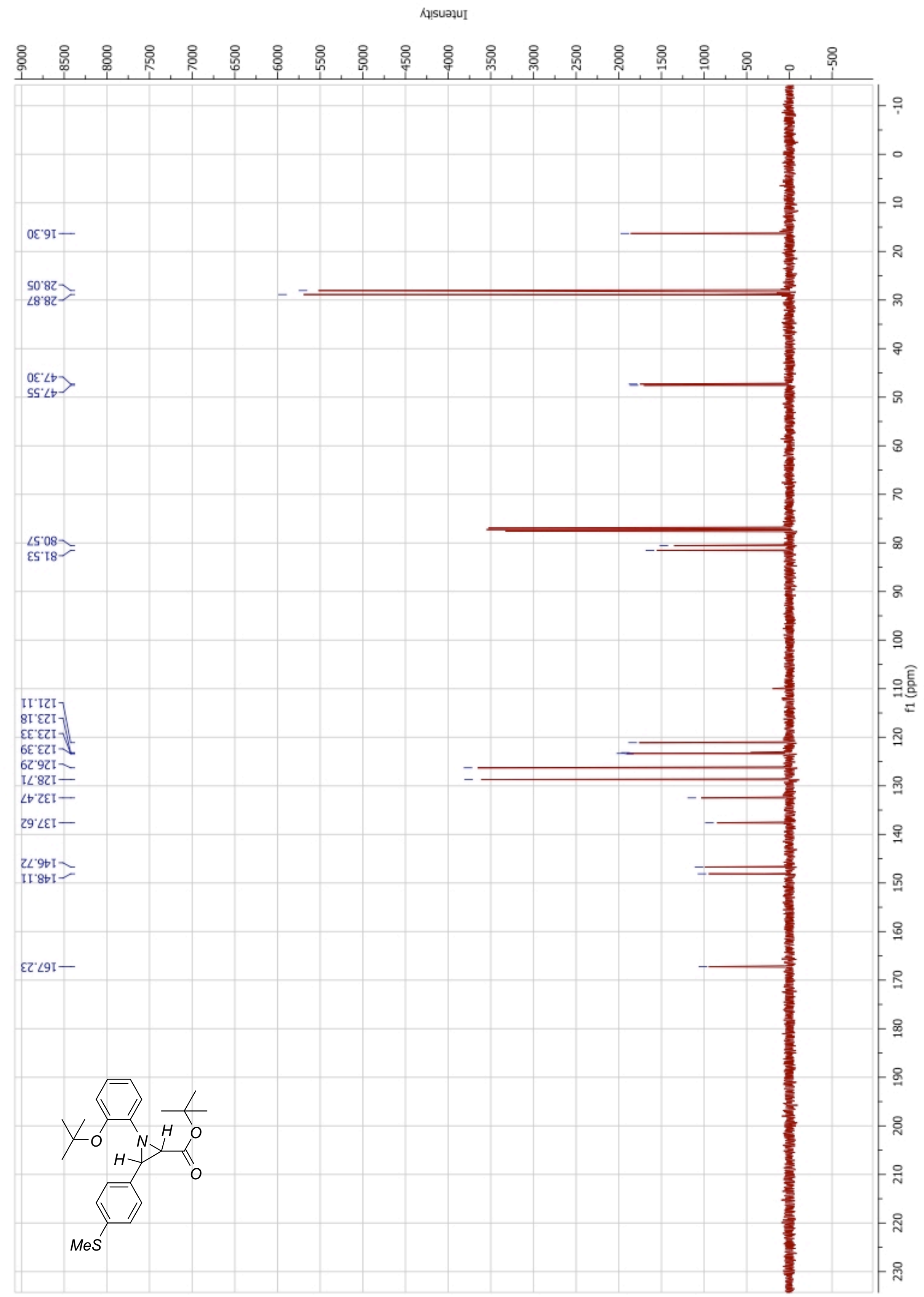
Intensity
f1 (ppm)
16.30
28.05
28.87
47.30
47.55
80.57
81.53
121.11
123.18
123.33
123.39
126.29
128.71
132.47
137.62
146.72
148.11
167.23
MeS

^{13}C-NMR of **49**

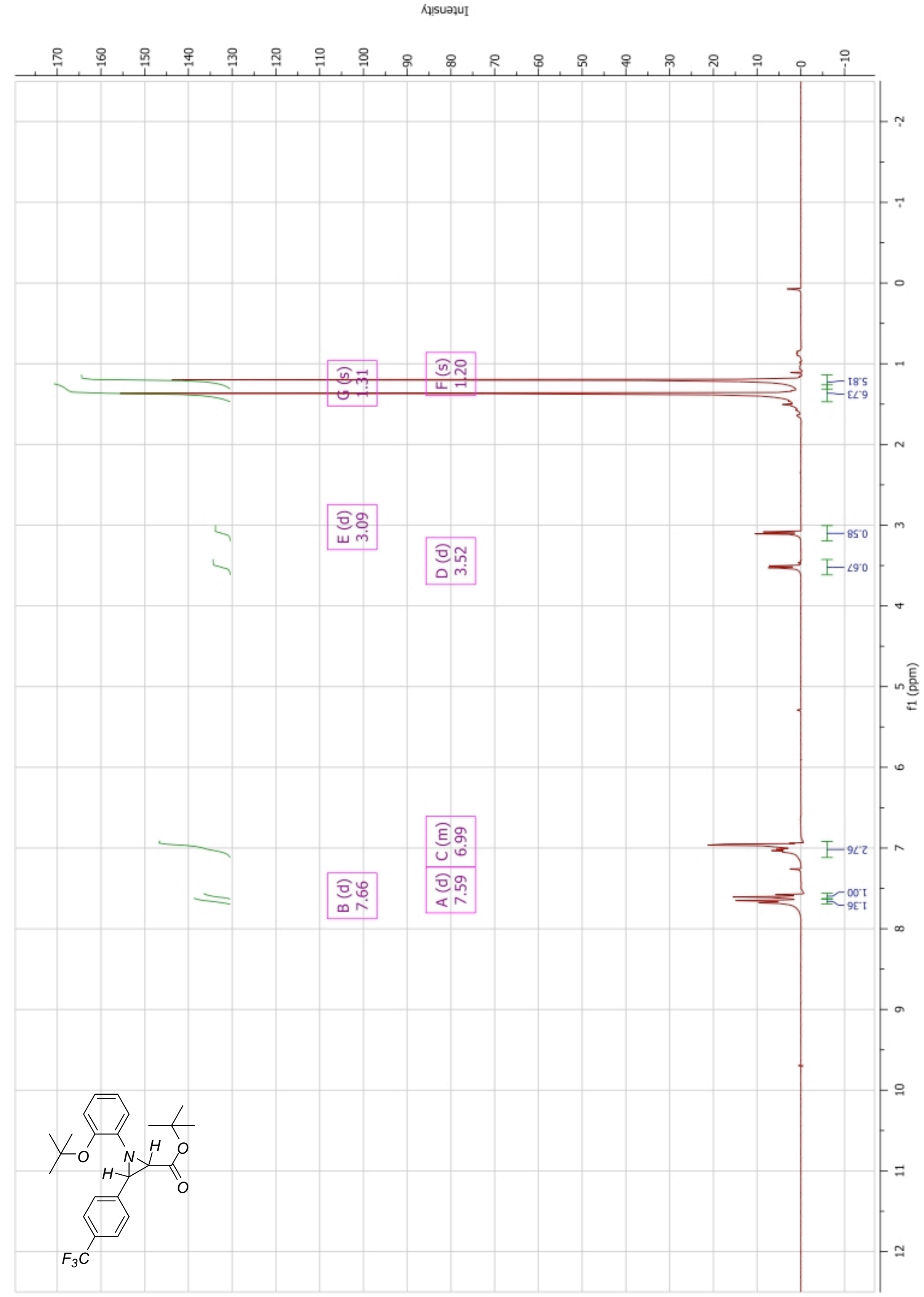
Intensity
f1 (ppm)
G (s) 1.31
F (s) 1.20
E (d) 3.09
D (d) 3.52
C (m) 6.99
A (d) 7.59
B (d) 7.66
F3C

^{1}H-NMR of **50**

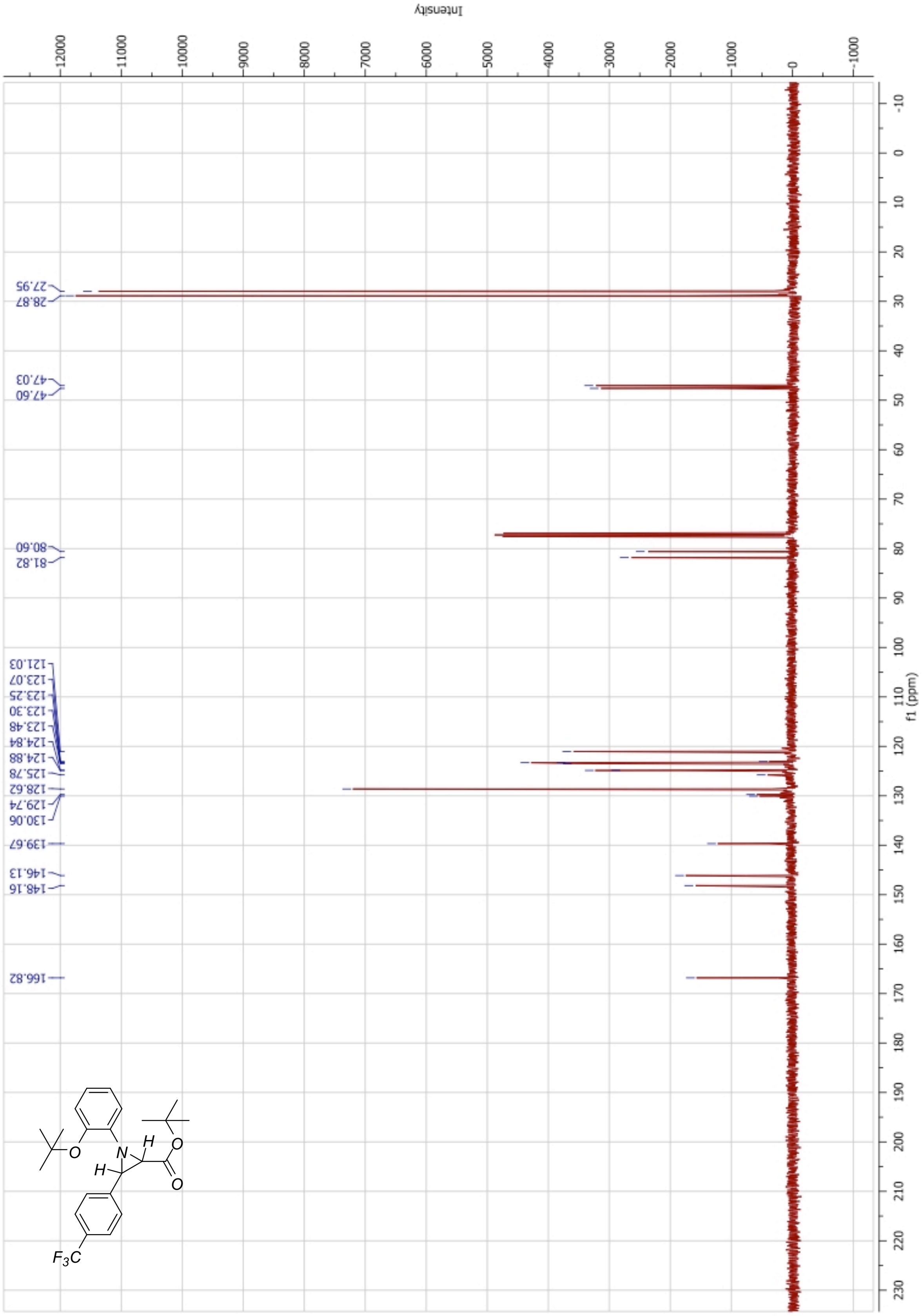
Intensity
f1 (ppm)
27.95
28.87
47.03
47.60
80.60
81.82
121.03
123.07
123.25
123.30
123.48
124.84
124.88
125.78
128.62
129.74
130.06
139.67
146.13
148.16
166.82
F_3C

^{13}C-NMR of **50**

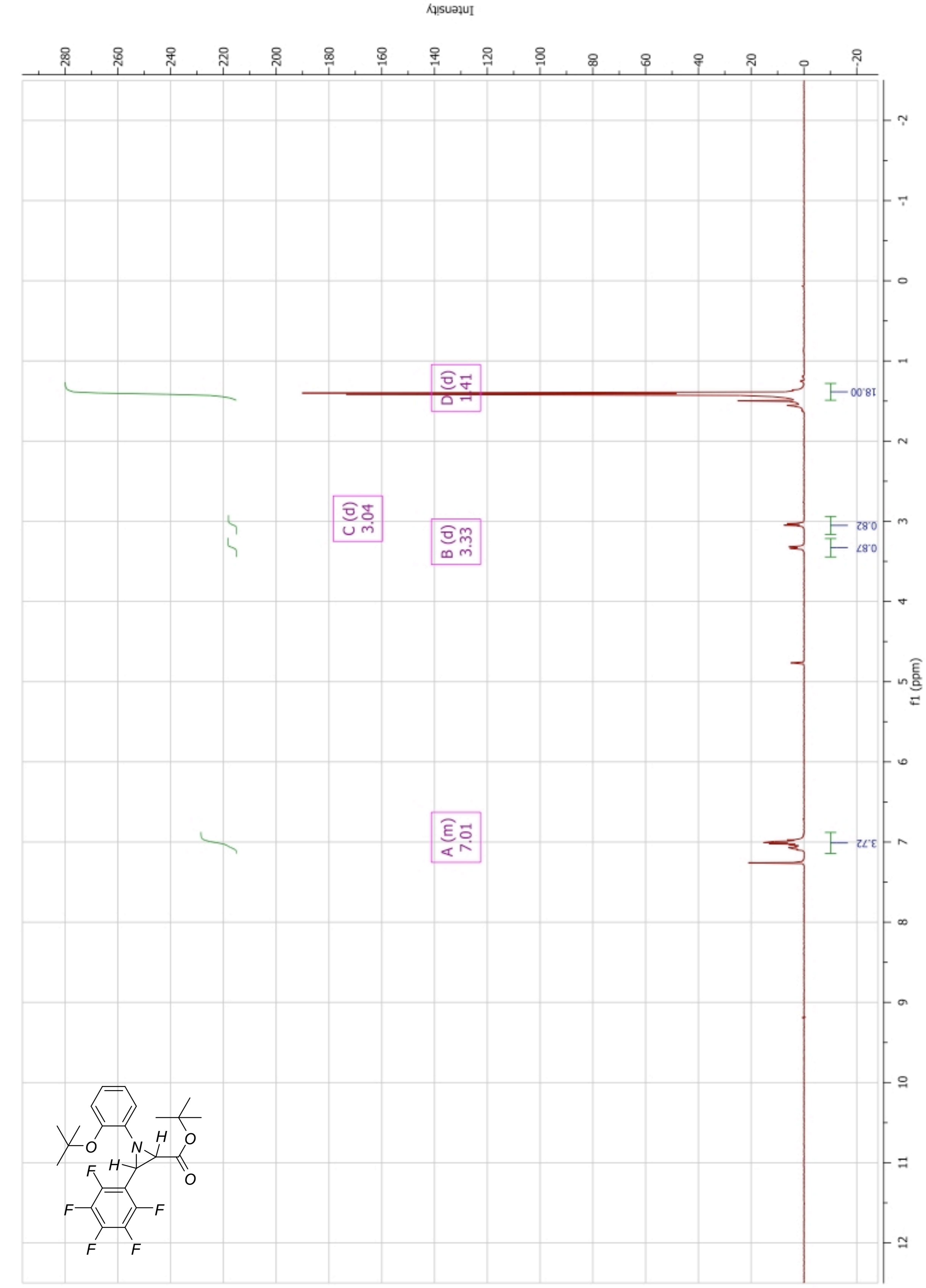

^{1}H-NMR of tert-butyl ester of **51**

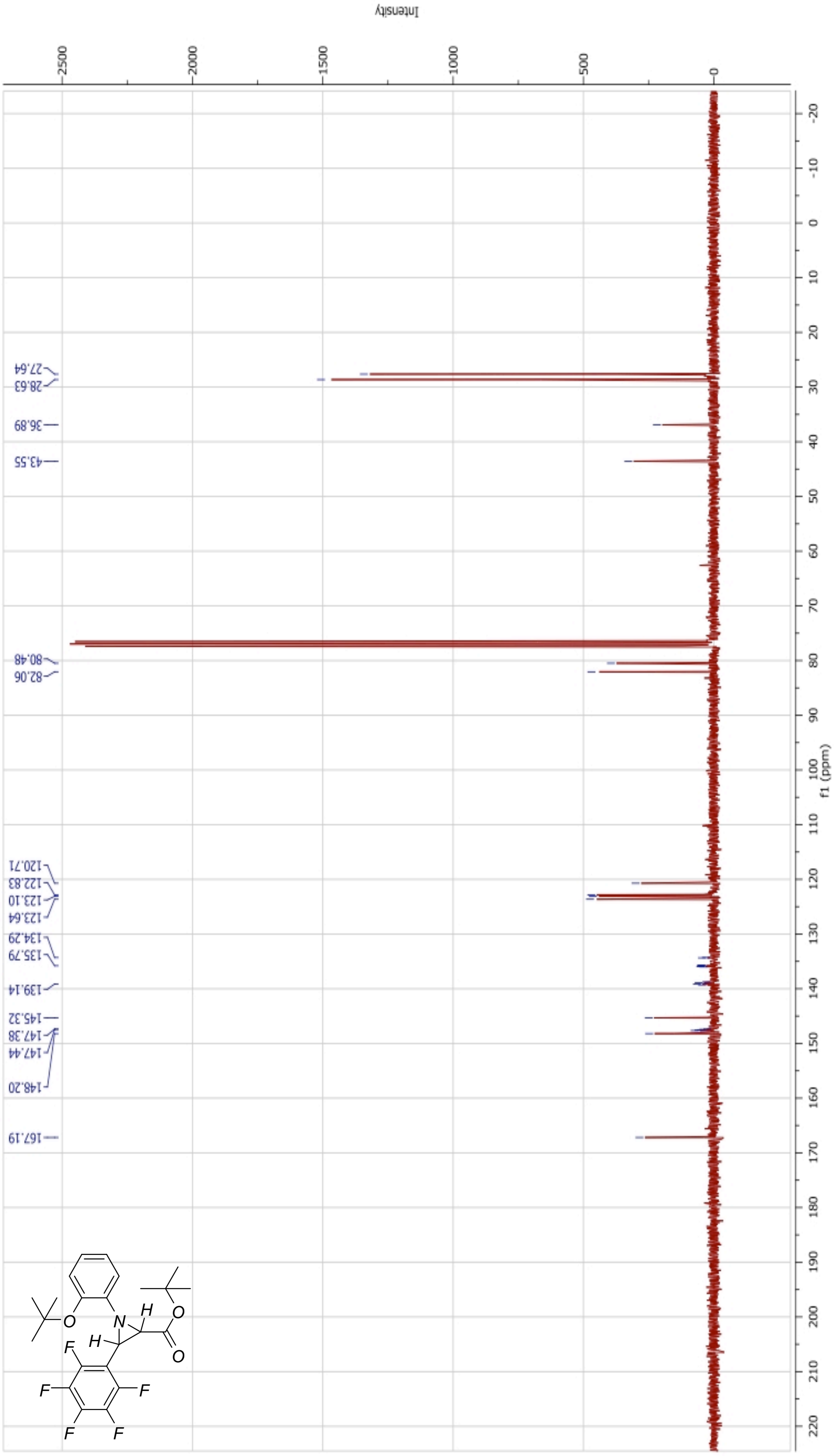

^{13}C-NMR of tert-butyl ester of **51**

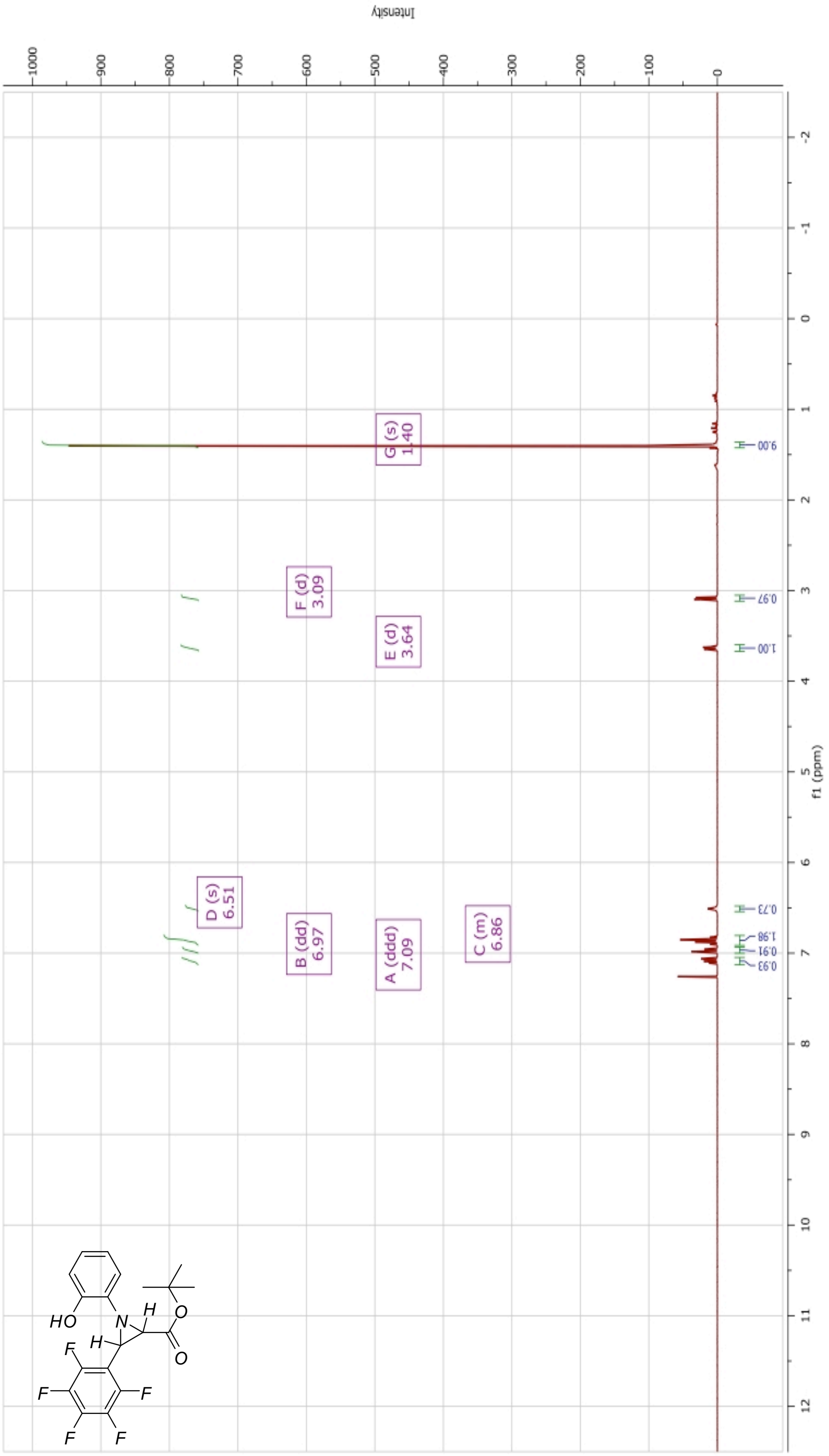

Intensity
f1 (ppm)
G (s) 1.40
F (d) 3.09
E (d) 3.64
D (s) 6.51
B (dd) 6.97
A (ddd) 7.09
C (m) 6.86
9.00
0.97
1.00
0.73
1.98
0.91
0.93
HO
N
H
O
O
F
F
F
F
F

^{1}H-NMR of **51**

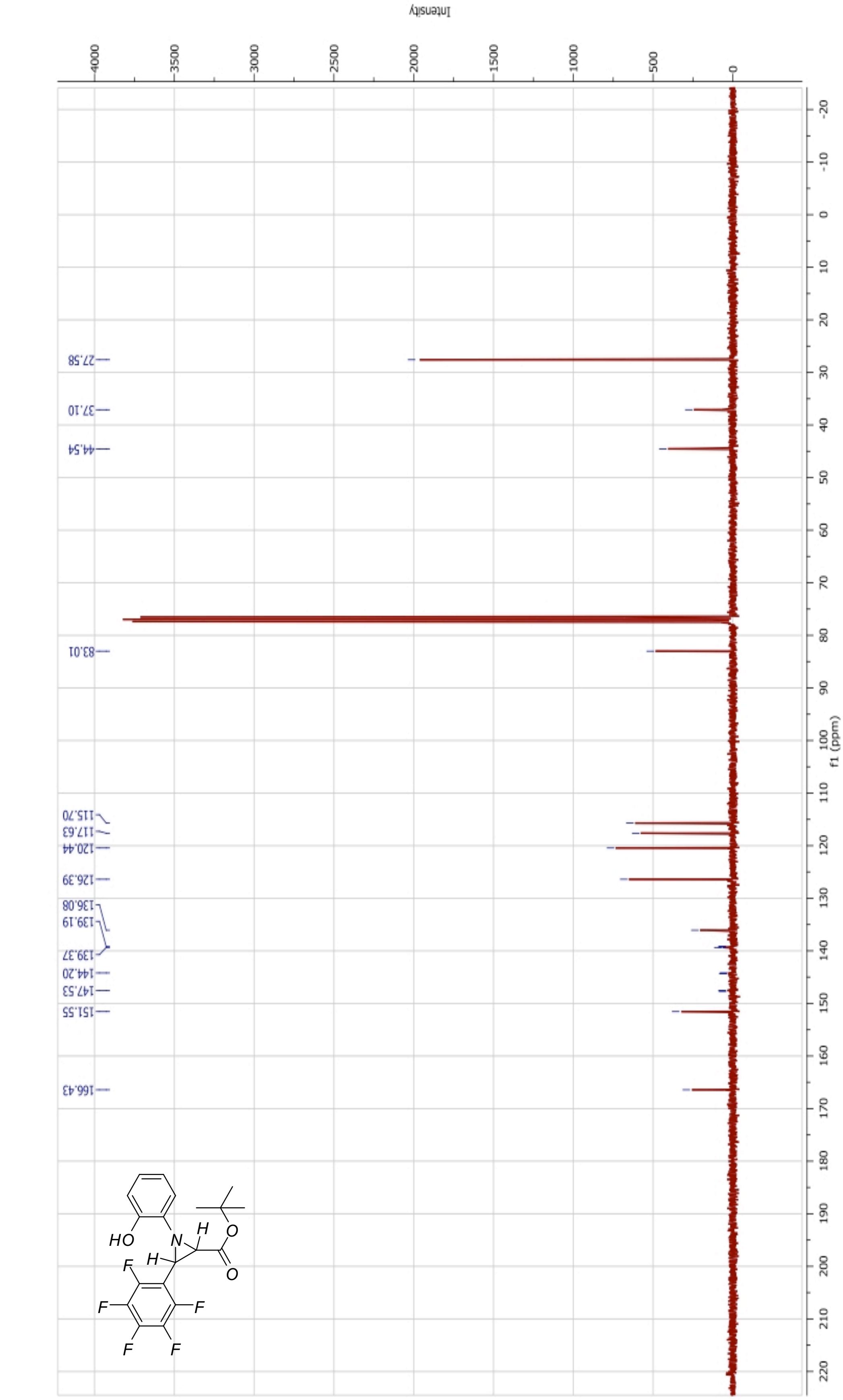
Intensity
f1 (ppm)
27.58
37.10
44.54
83.01
115.70
117.63
120.44
126.39
136.08
139.19
139.37
144.20
147.53
151.55
166.43
HO
N
H
H
O
O
F
F
F
F
F

^{13}C-NMR of **51**

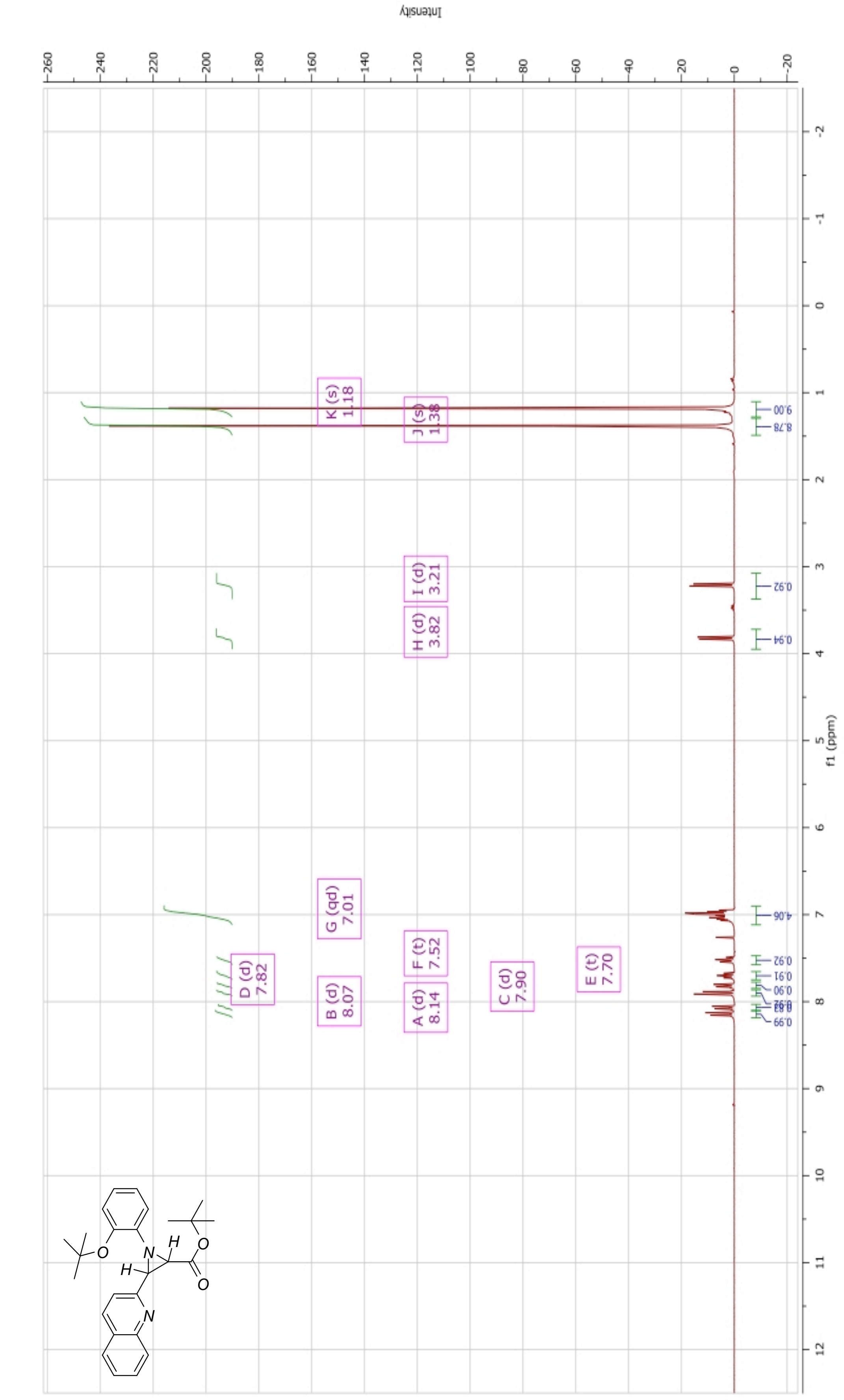
Intensity
f1 (ppm)
K (s) 1.18
J (s) 1.38
I (d) 3.21
H (d) 3.82
G (qd) 7.01
F (t) 7.52
E (t) 7.70
D (d) 7.82
C (d) 7.90
B (d) 8.07
A (d) 8.14
9.00
8.78
0.92
0.94
4.06

^{1}H-NMR of **52**

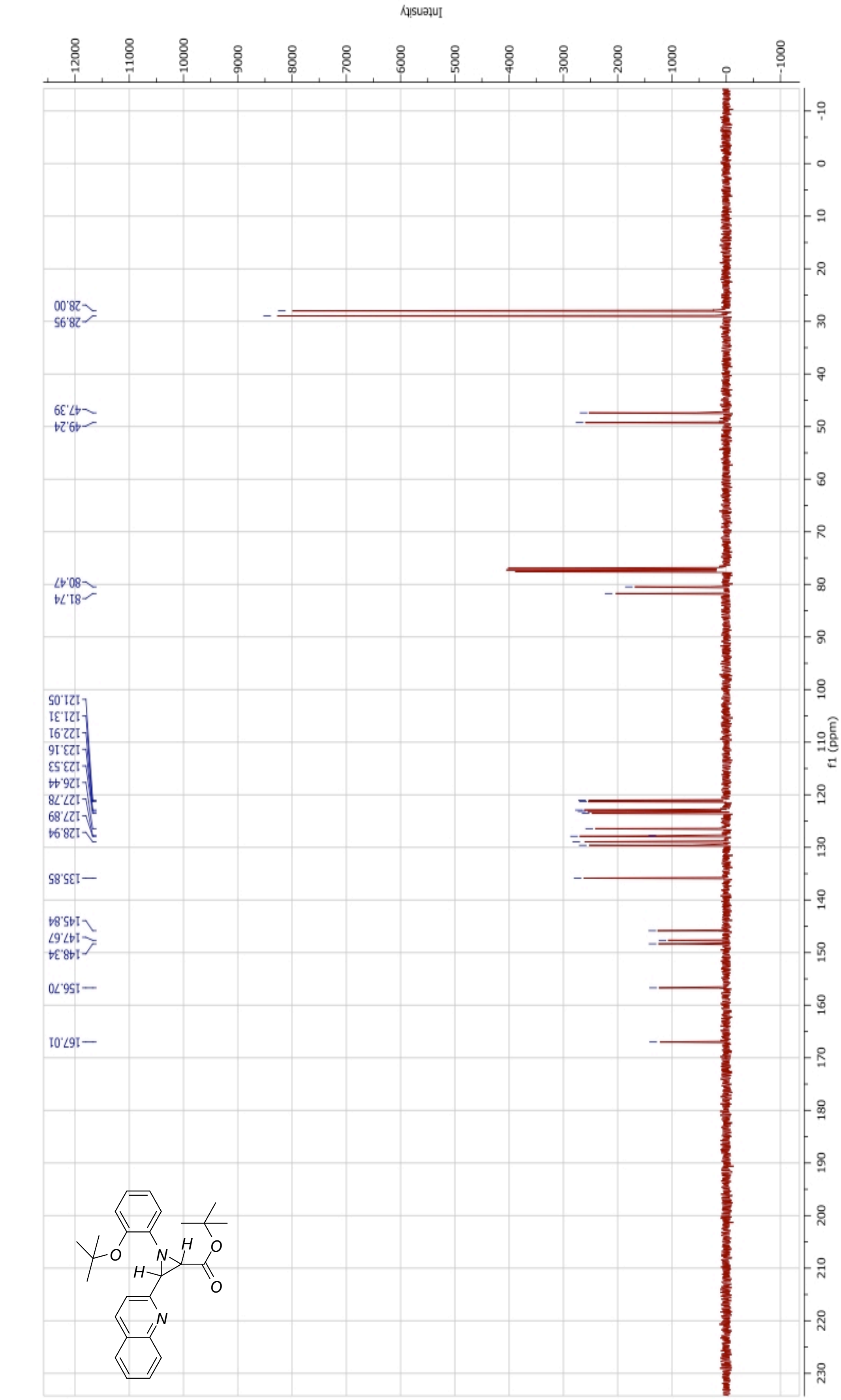
Intensity
f1 (ppm)
28.00
28.95
47.39
49.24
80.47
81.74
121.05
121.31
122.91
123.16
123.53
126.44
127.78
127.89
128.94
135.85
145.84
147.67
148.34
156.70
167.01

^{13}C-NMR of **52**

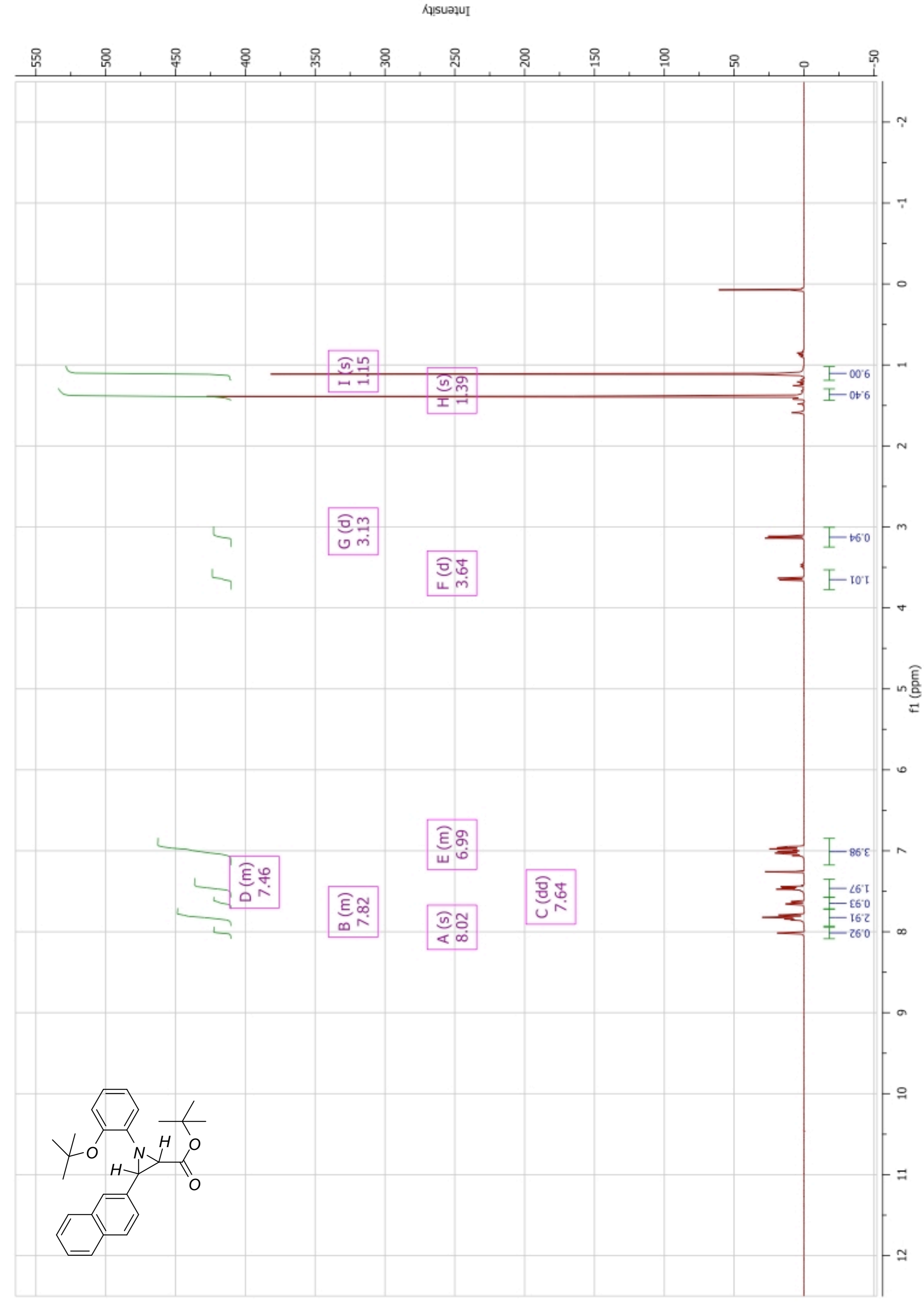
Intensity
f1 (ppm)
I (s) 1.15
H (s) 1.39
G (d) 3.13
F (d) 3.64
E (m) 6.99
D (m) 7.46
C (dd) 7.64
B (m) 7.82
A (s) 8.02
9.00
9.40
0.94
1.01
3.98
1.97
0.93
2.91
0.92

^{1}H-NMR of **53**

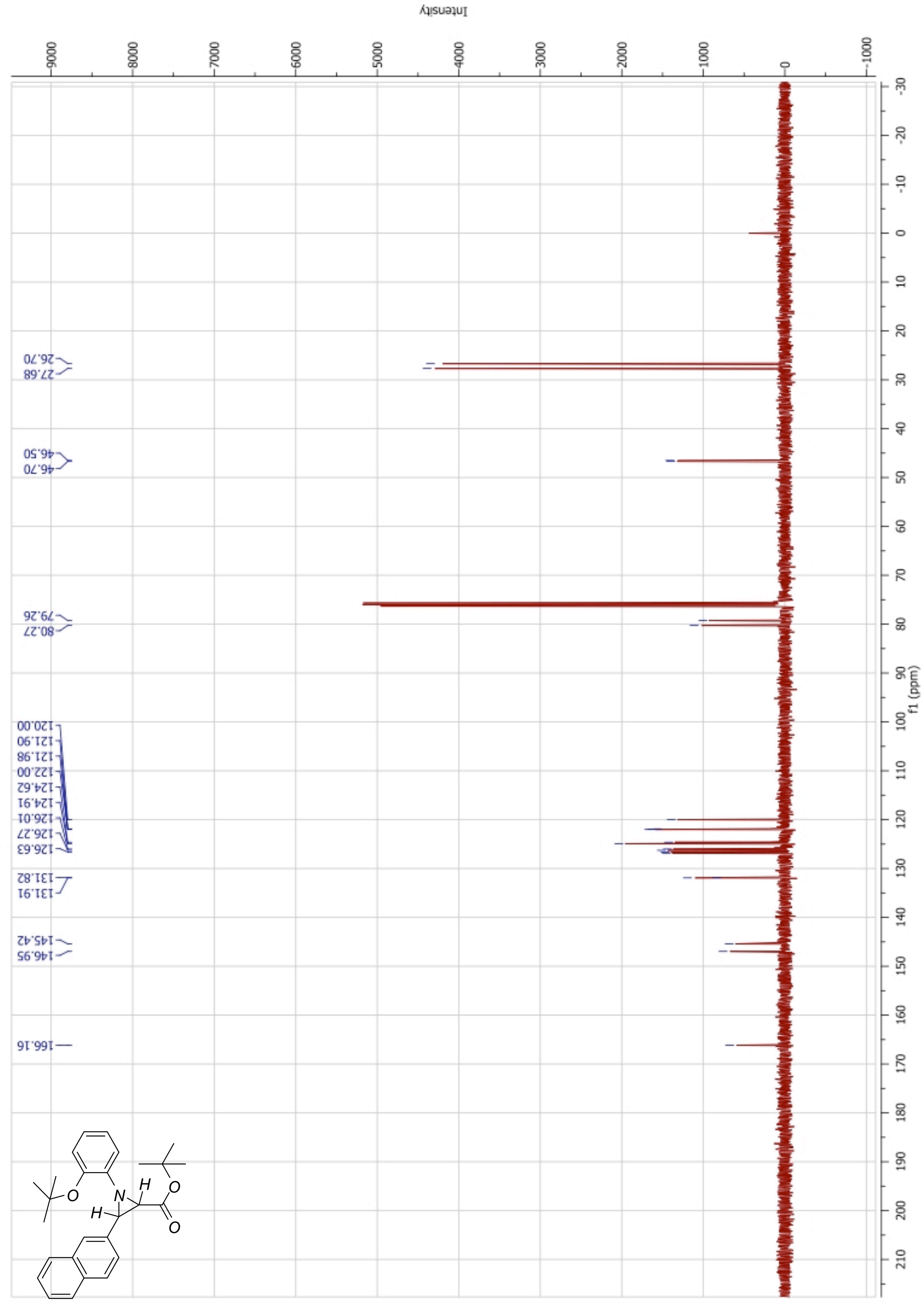
26.70
27.68
46.50
46.70
79.26
80.27
120.00
121.90
121.98
122.00
124.62
124.91
126.01
126.27
126.63
131.82
131.91
145.42
146.95
166.16
Intensity
f1 (ppm)

^{13}C-NMR of **53**

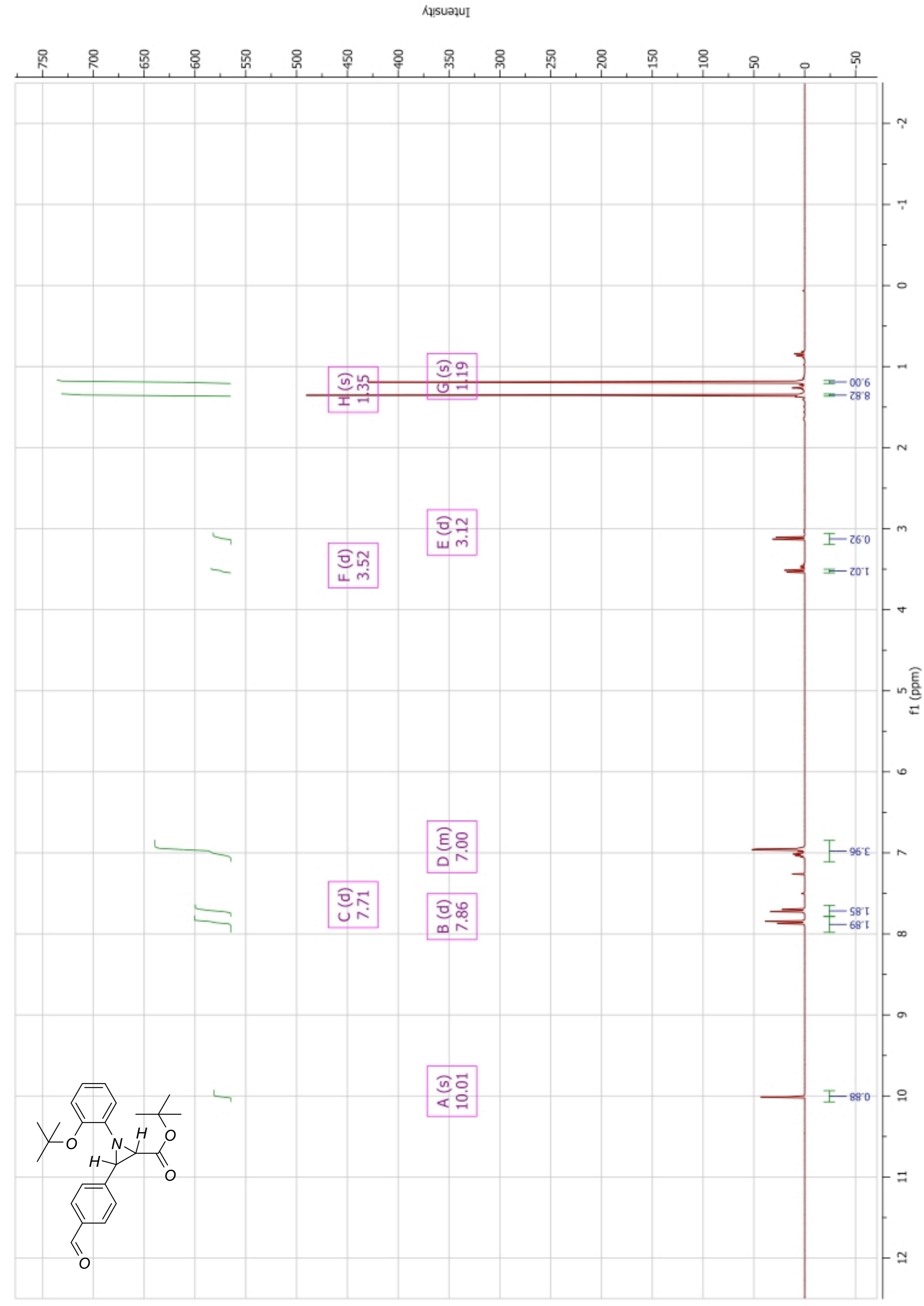
Intensity
f1 (ppm)
A (s) 10.01
B (d) 7.86
C (d) 7.71
D (m) 7.00
F (d) 3.52
E (d) 3.12
H (s) 1.35
G (s) 1.19
0.88
1.89
1.85
3.96
1.02
0.92
8.82
9.00

^{1}H-NMR of **54**

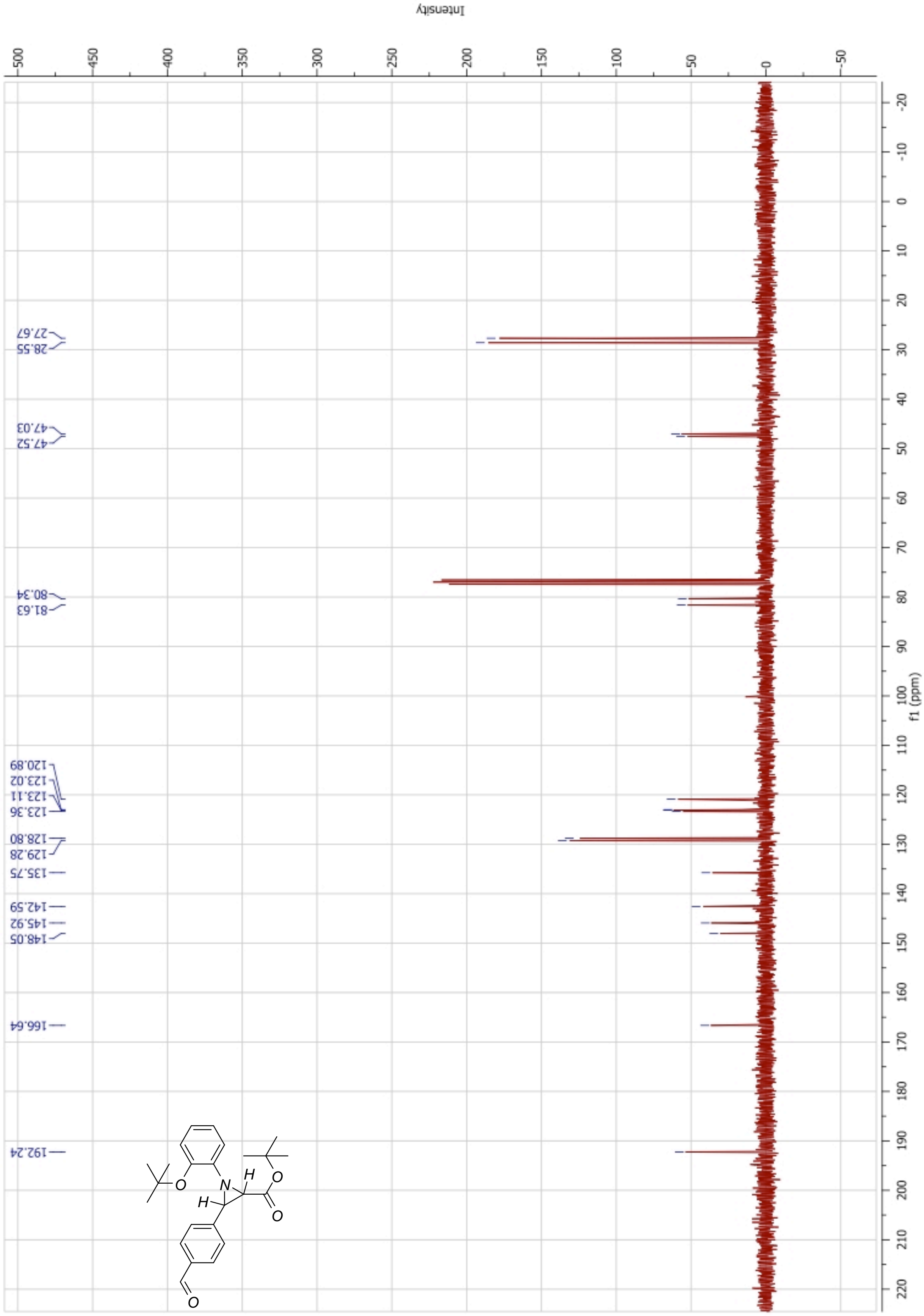
27.67
28.55
47.03
47.52
80.34
81.63
120.89
123.02
123.11
123.36
128.80
129.28
135.75
142.59
145.92
148.05
166.64
192.24
Intensity
f1 (ppm)

^{13}C-NMR of **54**

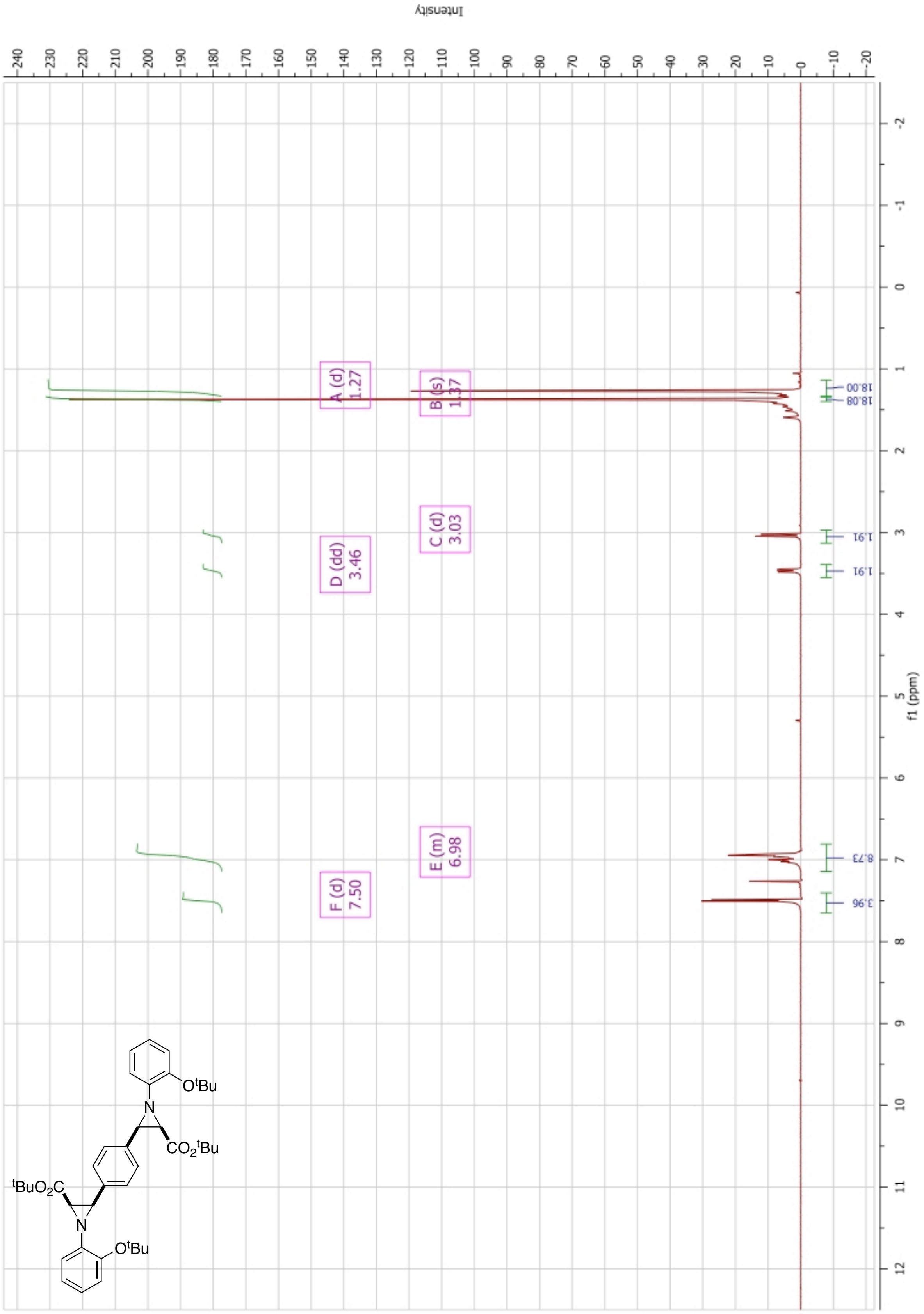
Intensity
f1 (ppm)
A (d)
1.27
B (s)
1.37
C (d)
3.03
D (dd)
3.46
E (m)
6.98
F (d)
7.50
18.00
18.08
1.91
1.91
8.73
3.96
O^{t}Bu
N
$CO_2{}^tBu$
$^tBuO_2C$
N
O^{t}Bu

^{1}H-NMR of **55**

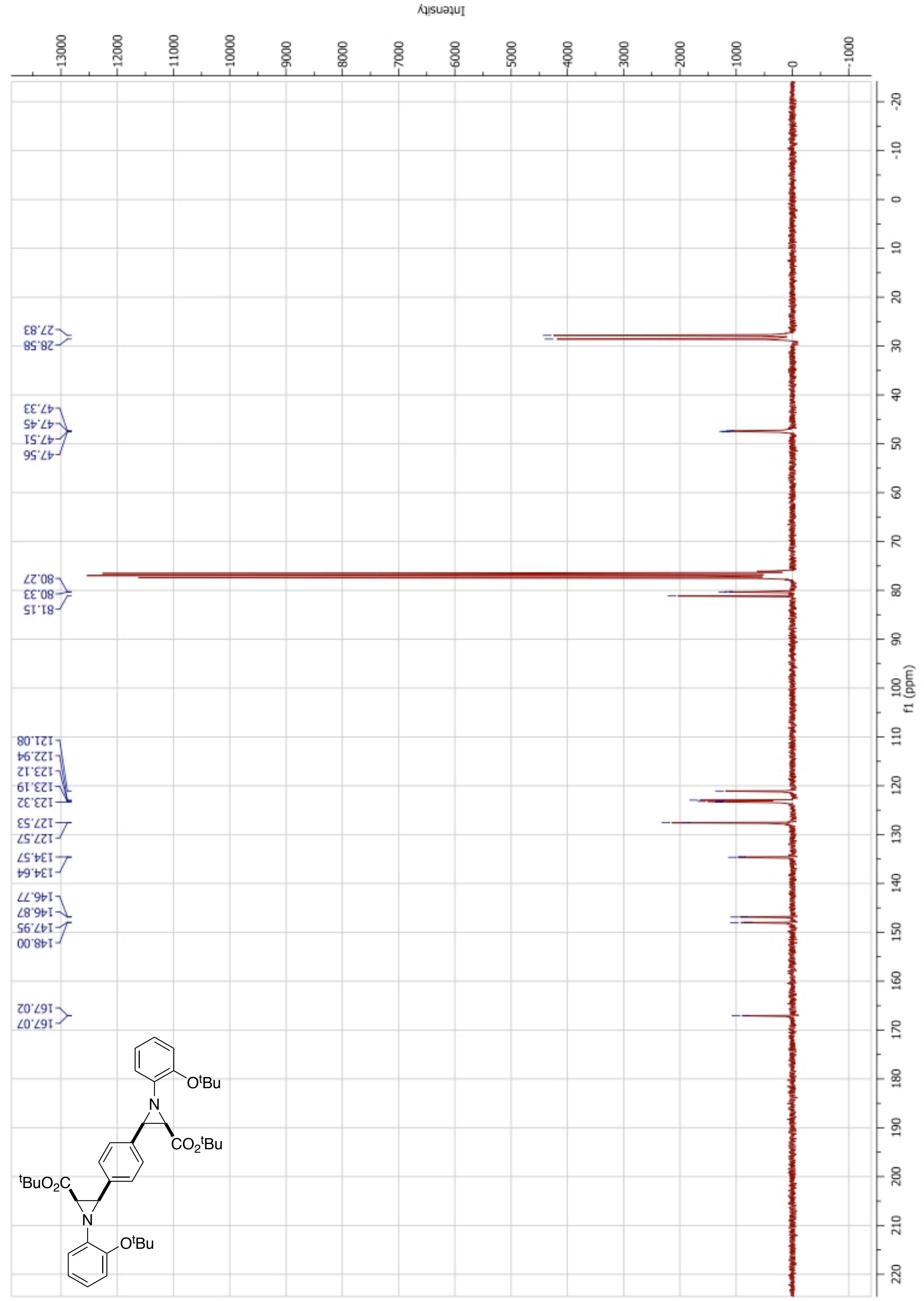
Intensity
f1 (ppm)
27.83
28.58
47.33
47.45
47.51
47.56
80.27
80.33
81.15
121.08
122.94
123.12
123.19
123.32
127.53
127.57
134.57
134.64
146.77
146.87
147.95
148.00
167.02
167.07
O^{t}Bu
N
$CO_2{}^tBu$
$^tBuO_2C$
N
O^{t}Bu

^{13}C-NMR of **55**

1

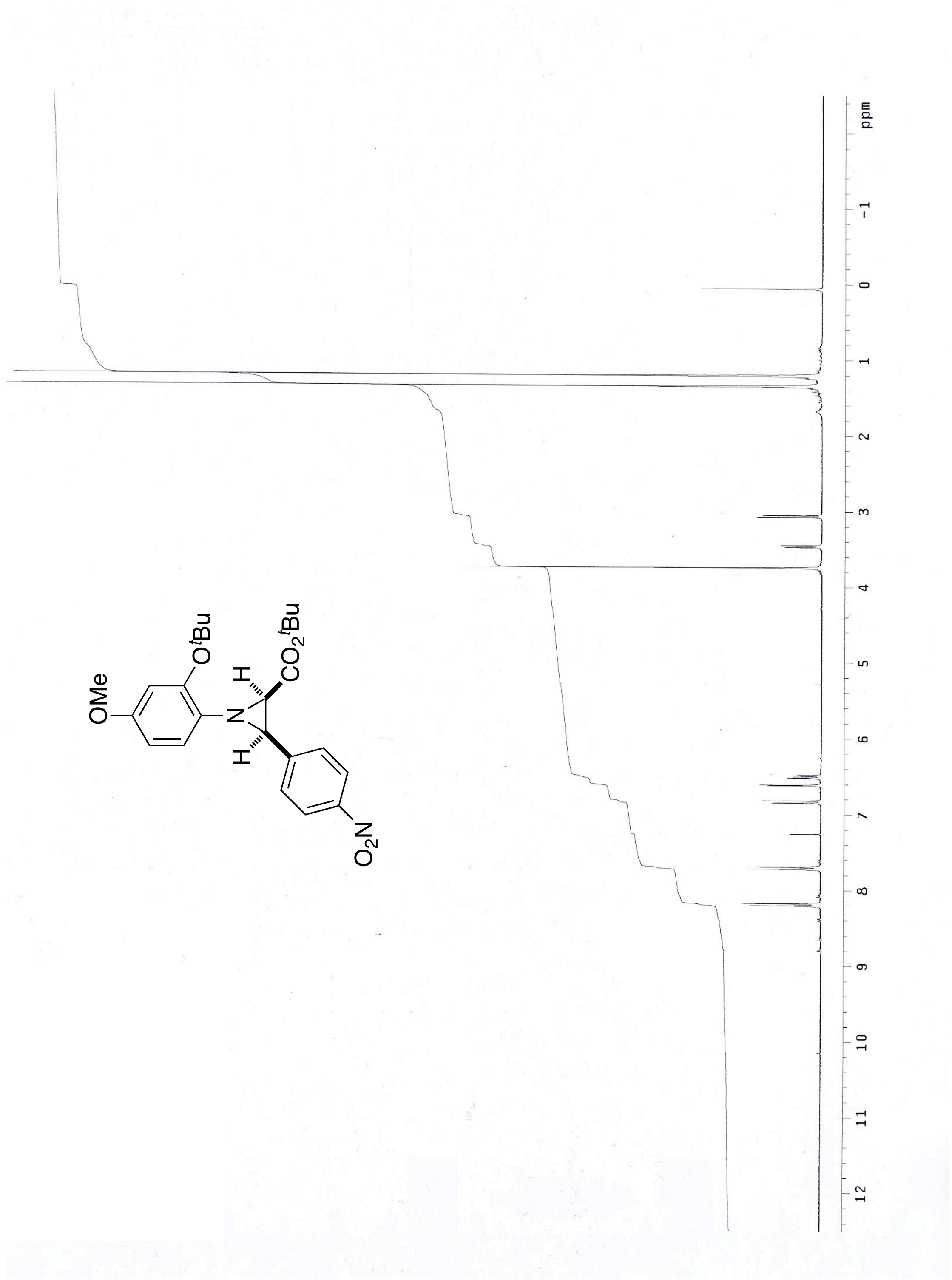

^{1}H-NMR of **57**

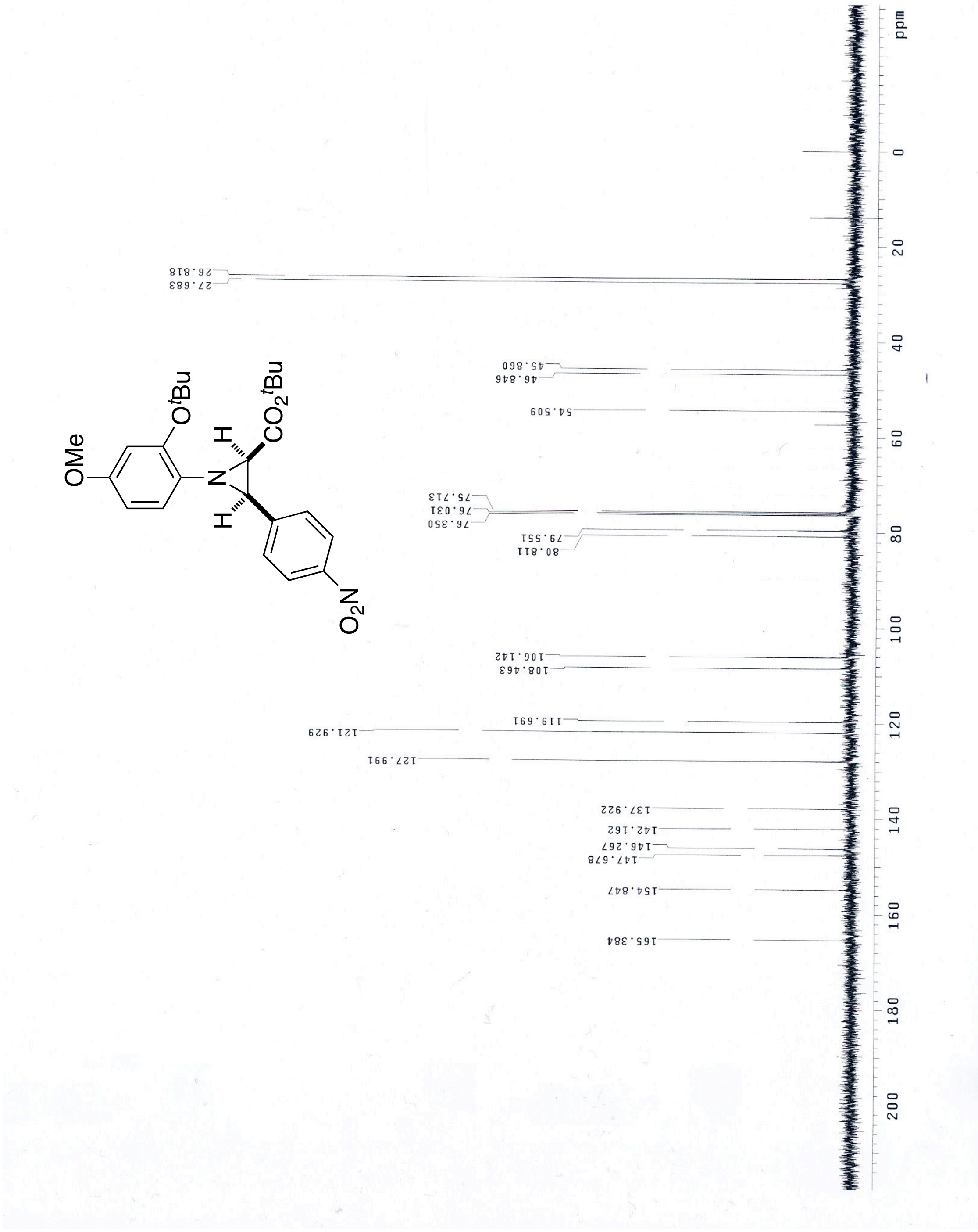

^{13}C-NMR of **57**

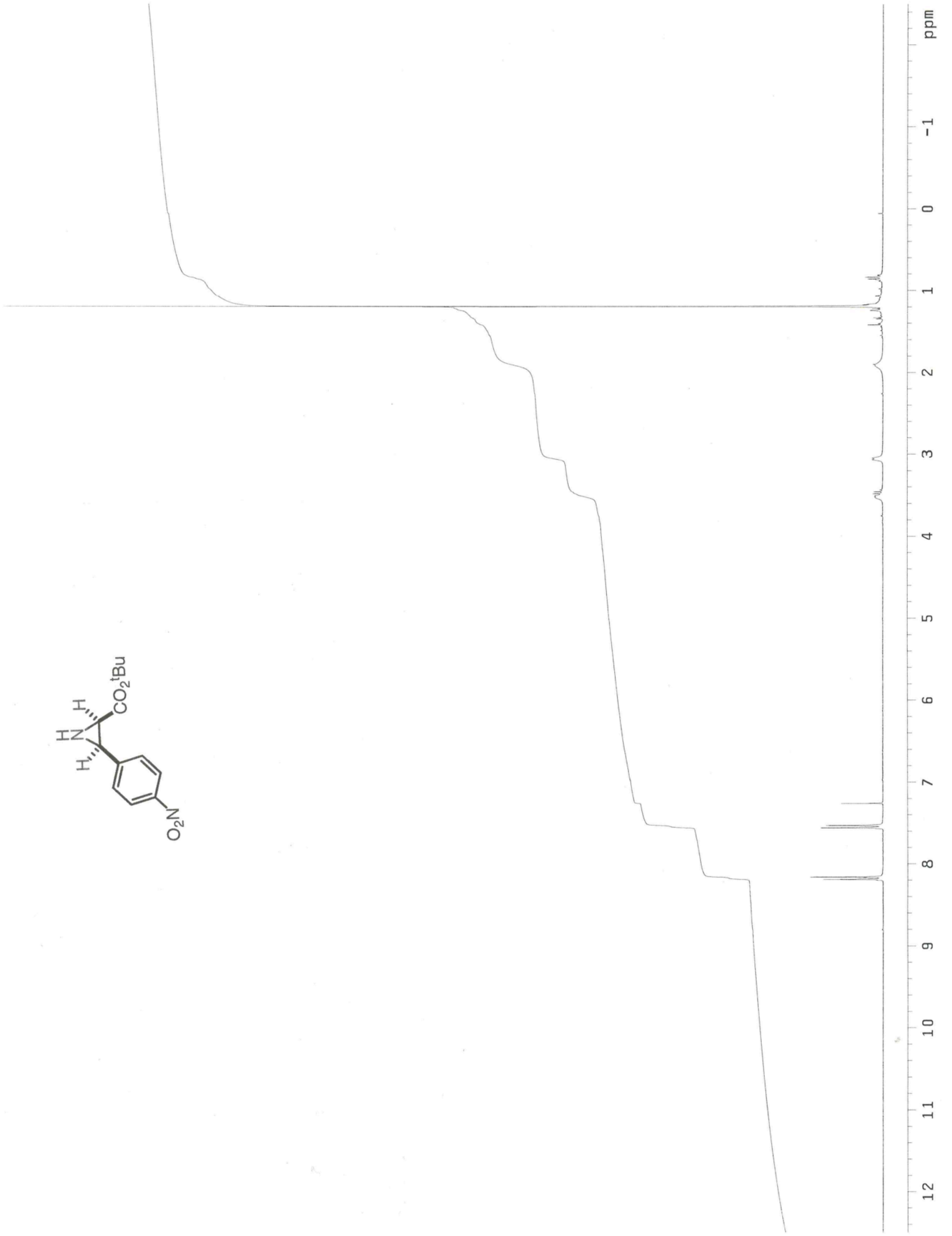

^{1}H-NMR of **58**

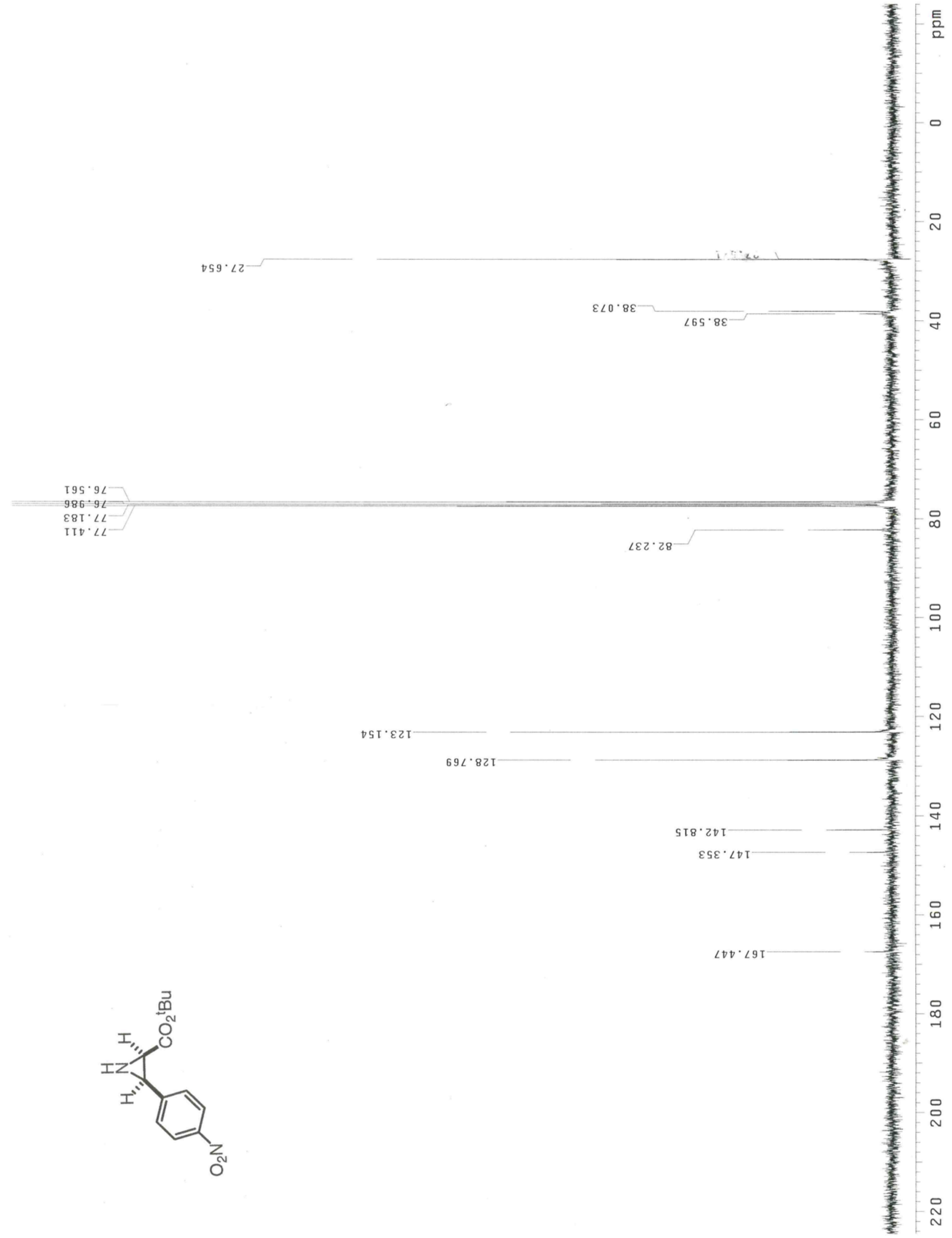

27.654
38.073
38.597
76.561
76.986
77.183
77.411
82.237
123.154
128.769
142.815
147.353
167.447
ppm

^{13}C-NMR of **58**

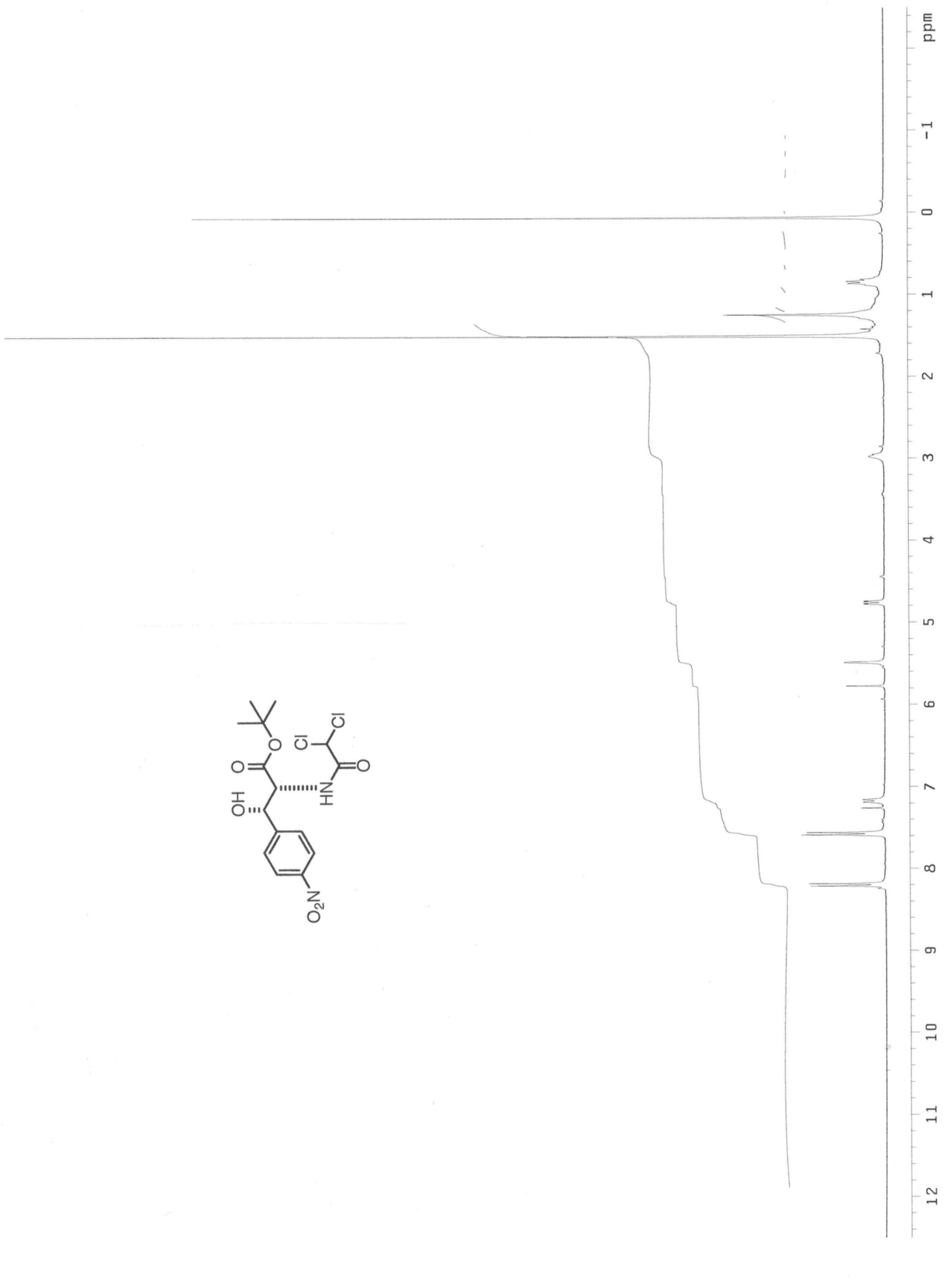

^{1}H-NMR of **59**

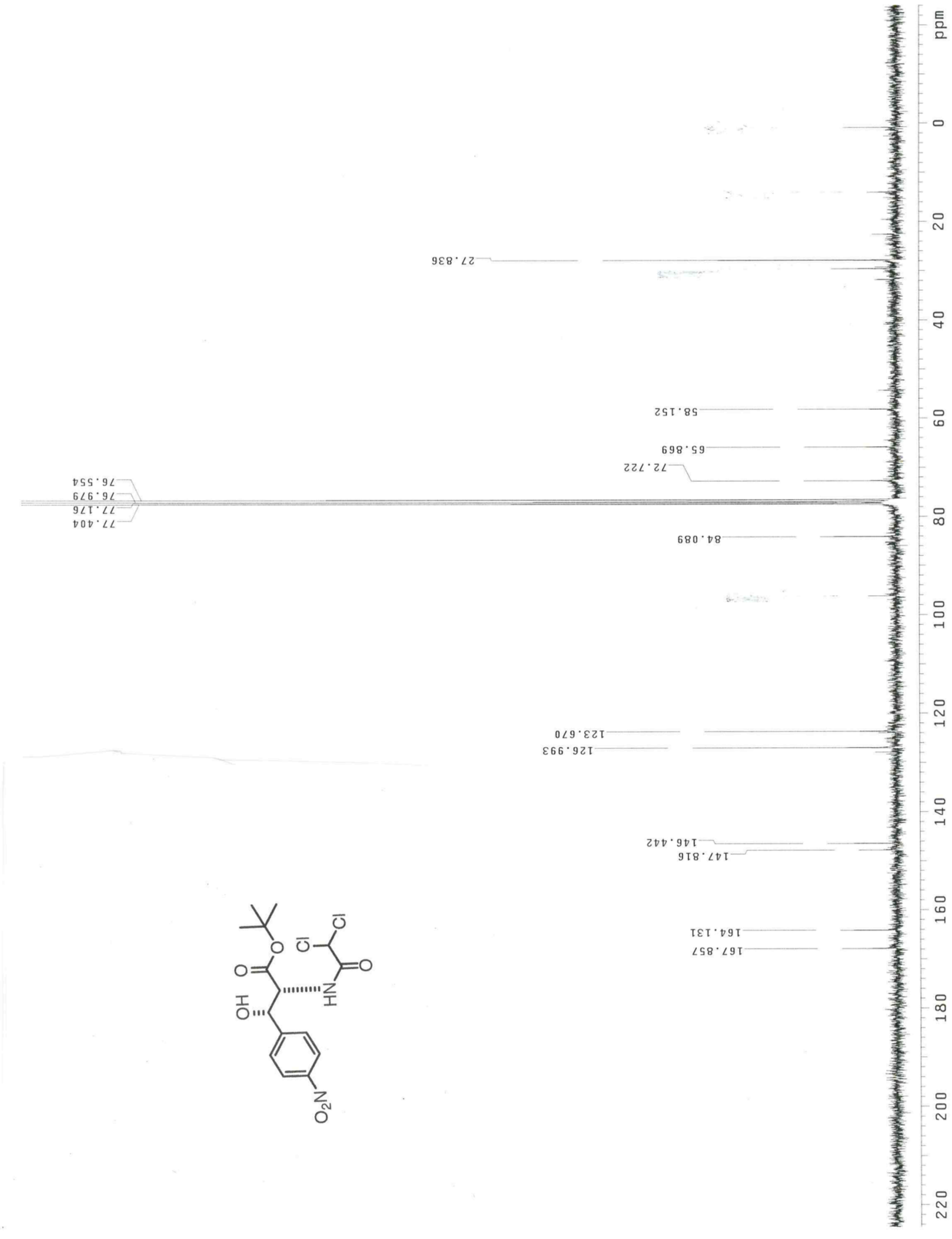
27.836
58.152
65.869
72.722
76.554
76.979
77.176
77.404
84.089
123.670
126.993
146.442
147.816
164.131
167.857
ppm

^{13}C-NMR of **59**

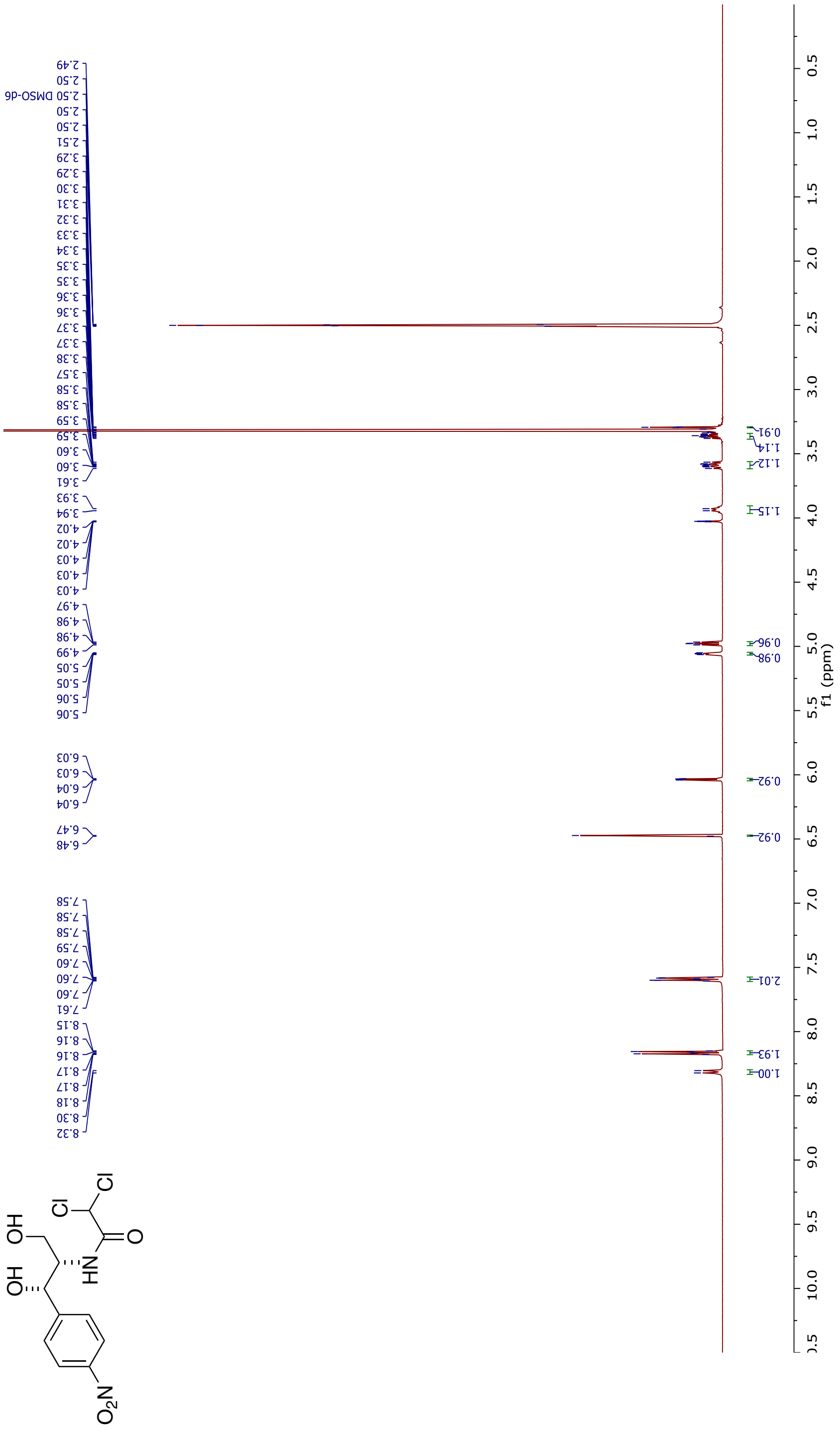

^{1}H-NMR of **60**

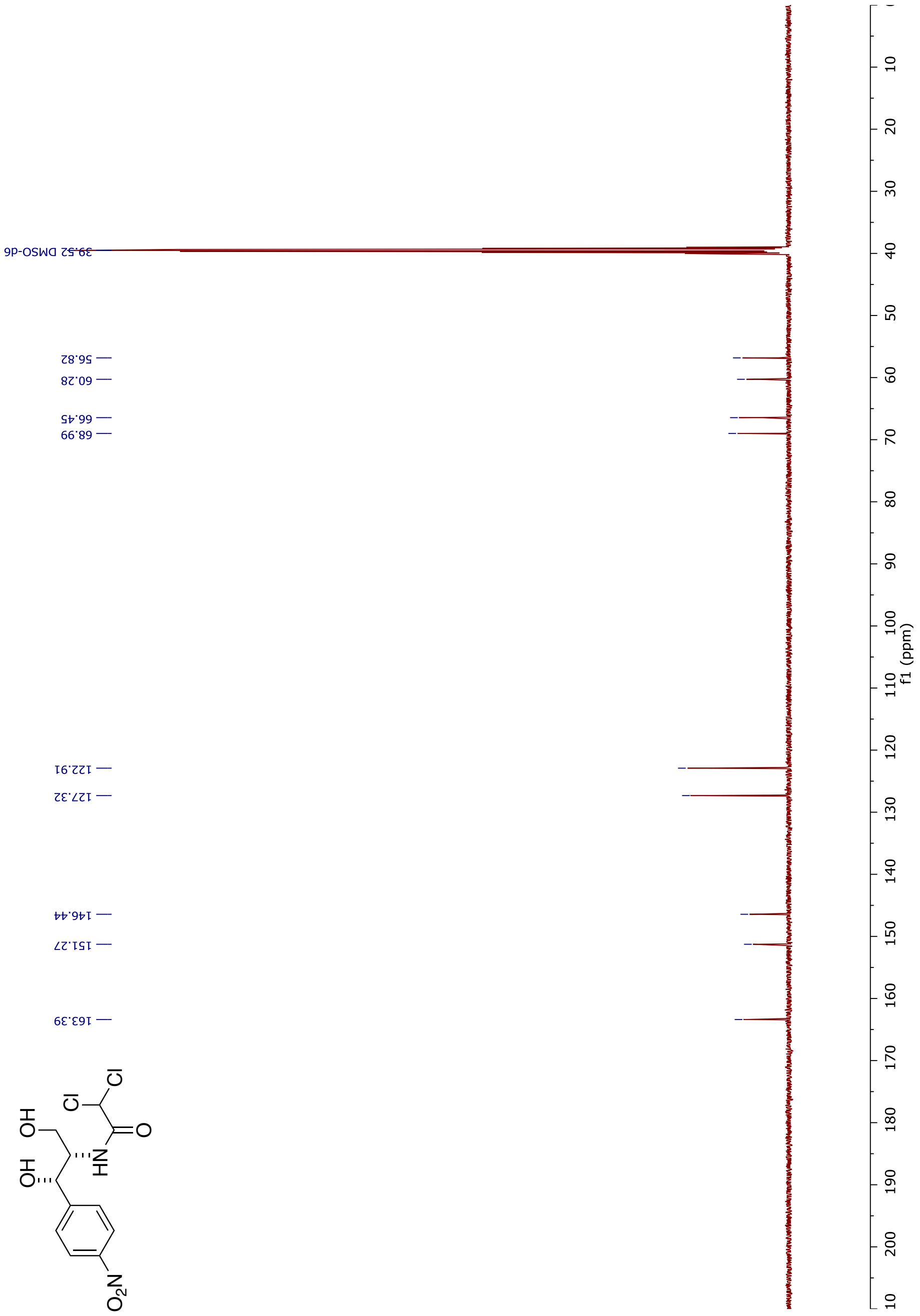
39.52 DMSO-d6
56.82
60.28
66.45
68.99
122.91
127.32
146.44
151.27
163.39
f1 (ppm)

^{13}C-NMR of **60**
